# B-cell peptide epitopes as diagnostic targets for Q fever in sheep

**DOI:** 10.3389/fmicb.2026.1751544

**Published:** 2026-02-17

**Authors:** Tamara Kozytska, Claudia Gerlach, Maksym Danchenko, Gabriela Flores-Ramirez, Hanka Brangsch, Heinrich Neubauer, Martin Pfeffer, Mathias W. Pletz, Ludovit Skultety, Katja Mertens-Scholz

**Affiliations:** 1 Friedrich-Loeffler-Institut, Institute of Bacterial Infections and Zoonoses, Jena, Germany; 2 Institute for Animal Hygiene and Veterinary Public Health, Faculty of Veterinary Medicine, University of Leipzig, Leipzig, Germany; 3 Institute of Virology, Biomedical Research Center, Slovak Academy of Sciences, Bratislava, Slovakia; 4 Institute for Infectious Diseases and Infection Control and Center for Sepsis Care and Control (CSCC), Jena University Hospital, Jena, Germany; 5 Institute of Microbiology, Czech Academy of Sciences, Prague, Czechia

**Keywords:** B-cell epitopes, *Coxiella burnetii*, ELISA, peptide array, proteomics, Q fever, serology

## Abstract

**Introduction:**

Q fever, caused by *Coxiella burnetii*, is a zoonotic disease of global relevance with domestic ruminants as the main reservoirs. Serological diagnosis, especially enzyme-linked immunosorbent assay (ELISA), often suffers from limited sensitivity and specificity due to antigenic variability and cross-reactivity.

**Scientific goal:**

In this study, a combined proteomic and literature research approach was used to identify immunoreactive proteins and predict linear B-cell epitopes as alternative diagnostic targets.

**Methods:**

Total protein extracts of a *C. burnetii* field isolate from sheep were separated by two-dimensional gel electrophoresis, and immunoreactive proteins were detected by Western blotting using pooled sheep sera obtained from various flocks with known Q fever status. Immunoreactive proteins were identified by LC–MS/MS and used for linear B-cell epitope prediction for peptide synthesis. Peptides (*n =* 30) were initially screened by fluorescent ELISA against nine field serum pools (90 individual sera), and the most promising peptides (*n =* 15) were individually tested with 79 single sera. Diagnostic performance was assessed by receiver operating characteristic (ROC) analysis and by a multi-peptide rule (“positive if ≥1 peptide reactive”).

**Results:**

A total of 156 seroreactive proteins, including 51 previously reported antigens, were detected, among others, Com1, CBU_0482, and Mip. Although the selected 15 peptides showed a specific reaction with pooled sera, they showed limited diagnostic performance with an area under the curve (AUC) of 0.5–0.7 when using single serum samples (*n =* 79). Multi-peptide combinations (6–8 peptides) increased sensitivity (Se) to 80% and specificity (Sp) to 75%.

**Discussion:**

Although single peptides lacked discriminatory power, multi-epitope combinations reached acceptable accuracy and may be used as a complementary tool for commercial ELISAs. However, larger bioinformatic approaches and validation studies are required to identify specific peptides of high diagnostic accuracy.

## 1 Introduction

*Coxiella burnetii* is a Gram-negative, obligate intracellular pathogen and the causative agent of Q fever, a zoonotic disease that affects both humans and animals. Although human infections are often asymptomatic, the acute form can cause fever and atypical pneumonia, while chronic Q fever may lead to endocarditis. The likelihood of chronification and chronic fatigue syndrome after acute infection remains a matter of debate (Manchal et al., 2020; Ankert et al., 2022). Domestic ruminants, particularly sheep, goats, and cattle, are the main reservoirs for human infections. The pathogen is shed in milk, feces, and especially in high numbers within birth fluids and tissues, making perinatal products a major source of environmental contamination and a source for transmission *via* inhalation of contaminated aerosols (Angelakis and Raoult, 2010).

*C. burnetii* has a unique intracellular life cycle, multiplying in acidified parasitophoric vacuoles of eukaryotic cells (Case and Samuel, 2016). The bacteria form spore-like small cell variants (SCVs), which are considered resistant to environmental stress to some extent and survive for long periods outside the host organism (von Sprockhoff, 1980; Coleman et al., 2004). With an infectious dose (ID₅₀) of 1–10 bacteria by inhalation, *C. burnetii* is considered one of the most infectious bacterial pathogens known today (Jones et al., 2006; Brooke et al., 2015). These properties have led to its classification as a Category B bioterrorism agent by the Centers for Disease Control and Prevention (CDC, USA) (Madariaga et al., 2003).

Q fever poses a diagnostic challenge in veterinary medicine because infected animals often exhibit no clinical signs, although abortions, weak offspring, and reduced fertility can occur (Arricau Bouvery et al., 2003; Rodolakis et al., 2007). Massive bacterial shedding occurs even in asymptomatic animals (Rousset et al., 2009; Bauer et al., 2020). This complicates outbreak investigations and hinders the identification of infection sources, especially in the presence of seronegative shedders (Maurin and Raoult, 1999; Niemczuk et al., 2014). The European Food Safety Authority (EFSA) has recently classified Q fever as a priority zoonosis within the EU due to its impact on animal and public health (European Food Safety et al., 2023). This was exemplified for the livestock and public health sector during the largest ever reported Q fever outbreak in the Netherlands during 2007–2010 (van Asseldonk et al., 2013).

Commercially available serological tests used in veterinary diagnostics, such as enzyme-linked immunosorbent assay (ELISA), often show variable sensitivity (58–86%) and specificity (52–96%) due to antigenic variance of the strains used. For example, the comparison of the diagnostic performance of three commercial ELISA test kits for ruminants using different *C. burnetii* strains as antigen revealed differences in sensitivity and specificity among tests, also in regard to the host species examined. Using a cattle-derived *C. burnetii* isolate as antigen, sensitivities ranged between 86.9 and 90.5% for all three ruminant species examined. On the contrary, when using a tick- or sheep-derived antigen, the sensitivities were the lowest for sheep with 39.9% or 53.8%, respectively (Lurier et al., 2021). These differences are adjustable by species-specific cutoffs (Riviere et al., 2025). When comparing proteomic studies, immunogenic proteins show limited overlap between species. Of 169 described immunoreactive proteins, only 41% overlap between humans and mice or 38% between humans and guinea pigs. It was speculated that differences in the immune system of the three hosts may be responsible for the heterogeneity of antigen detection (Gerlach et al., 2017).

Currently used ELISA tests do not support differentiation between an acute or past infection, nor vaccination. For example, in human medicine, phase I and phase II antibody responses are used to differentiate between acute and chronic Q fever infections (Anderson et al., 2013). However, this differentiation is not applied in veterinary diagnostics. Whole-cell antigens include plenty of conserved proteins that can impair specificity. Cross-reactivity with related pathogens such as *Chlamydia* spp., *Legionella* spp., and *Bartonella* spp. was observed in studies that used ELISA and/or immunofluorescence assays (IFA) to analyze rabbit-, mouse-, or human-derived serum samples (La Scola and Raoult, 1996; Musso and Raoult, 1997; Lukacova et al., 1999; Horigan et al., 2011; Paul et al., 2013).

In addition to the elaborate production of *C. burnetii* whole-cell extracts under biosafety level (BSL) 3 conditions, these whole-cell antigens lack well-defined and reproducible antigens. Furthermore, only a fraction of epitopes is possibly accessible to antibodies due to the complex nature of the whole-cell antigen. This may finally impair the uniformity, sensitivity, and specificity of diagnostic results.

Recent efforts have focused on the use of recombinant proteins to address this problem. These approaches allow for the production of clearly defined and reproducible antigen components, opening up possibilities for highly sensitive and specific diagnostics (Vranakis et al., 2019; Stellfeld et al., 2020). However, production of recombinant high-quality proteins requires cloning, expression, and several purification steps. These are major technical hurdles when producing arrays on a large scale. Moreover, recombinant proteins often exhibit a limited antigenic spectrum and restricted epitope representation, which can reduce diagnostic sensitivity and increase production costs. Additionally, selected recombinant antigens may not be immunodominant throughout all stages of infection. On the contrary, the production of linear peptides is well established and fully automated. Immunoreactive proteins contain a specific region that is recognized by a B-cell receptor or antibody. This region can include non-sequential or sequential amino acid residues and is designated as conformational or linear B-cell epitopes (Sanchez-Trincado et al., 2017). These epitopes are typically surface exposed, structurally flexible, intrinsically disordered, and exhibit high sequence polymorphism. There are several algorithms available to identify these protein regions, e.g., BepiPred-2.0, COBEpro, or IUPred3. The latter has been successfully applied for species-specific serodiagnosis of *Chlamydia* (Sweredoski and Baldi, 2009; Jespersen et al., 2017; Rahman et al., 2018).

Applying this principle to *C. burnetii*, *in silico*–predicted B-cell epitopes from known immunogenic proteins from the literature and newly identified proteins using a proteomic approach were used to select peptide candidates for serodiagnosis of Q fever in sheep (Gerlach et al., 2017).

## 2 Materials and methods

### 2.1 Bacterial isolates and growth conditions

*C. burnetii* field isolate 26QC00015 originated from sheep afterbirth material collected in Thuringia, Germany, in 2009 and was obtained from the Friedrich-Loeffler-Institut strain collection. This isolate was propagated in mouse fibroblasts (L-929) in DMEM (Dulbecco’s Modified Eagle Medium, Lonza Group AG, Basel, Switzerland) supplemented with 5% fetal calf serum (FCS, Th. Geyer Ingredients GmbH & Co. KG, Höxter, Germany) at 37 °C and 5% CO_2_ until approximately 80% infectivity was reached. Briefly, 1e+05 L-929 cells/mL were infected with a multiplicity of infection (MOI) of 100, and after 12–14 days, *C. burnetii* was harvested and quantified *via* quantitative real-time PCR with *icd* as target (*icd*-qPCR) as described previously (Klee et al., 2006). The isolate was subsequently propagated in acidified citrate cysteine medium (ACCM-2, Sunrise Science Products, San Diego, CA, USA) as described elsewhere (Omsland et al., 2011). After 7–10 days, bacteria were harvested by centrifugation (10,000 × g, 20 min, 4 °C) and stored in sucrose–glycerol buffer (270 mM sucrose, 10% glycerol) at −80 °C. Propagation, handling, processing, and storage of *C. burnetii* were carried out under BSL-3 conditions.

### 2.2 Reagents

Acetonitrile (ACN), ammonium bicarbonate (NH4CO3), amidosulfobetaine (ASB-14), 5-bromo-4-chloro-3-indolyl phosphate (BCIP), bromophenol blue, chloroform, glycerol, dimethyl sulfoxide (DMSO), iodoacetamide, nitro blue tetrazolium chloride (NBT), phosphoric acid, polyethylene glycol (PEG), sodium dodecyl sulfate (SDS), sucrose, trifluoroacetic acid (TFA), tris(hydroxymethyl)aminomethane (TRIS), and TRIS–HCl were purchased from Merck KGaA, Darmstadt, Germany. Ammonium sulfate [(NH4)_2_SO_4_], dithiothreitol (DTT), ethanol, glycine, magnesium chloride hexahydrate (MgCl2 × 6H_2_O), methanol, non-fat milk powder, sodium chloride (NaCl), thiourea, TRIS base, Triton X-100, Tween 20, and urea were obtained from Carl Roth, Karlsruhe, Germany. Coomassie Brilliant Blue G250 was purchased from SERVA Electrophoresis GmbH, Heidelberg, Germany.

### 2.3 Serum samples and serum pools of known Q fever status

Ruminant sera of Q fever–positive and –negative herds consisting of 2–30 single sera were retained from the serum collection of the National Reference Laboratory for Q fever (Friedrich-Loeffler-Institut, Jena, Germany) and used for immunoproteomic analyses and peptide-epitope screenings using fluorescent peptide antigen ELISA. Each serum pool arose from a single herd of known Q fever positivity or from apparently healthy and Q fever–negative herds. Single serum samples (Supplementary Table S1) and pools of sera (Table 1, Supplementary Table S2) were tested using Q fever (*C. burnetii*) Ab Test (IDEXX Laboratories, Inc., Westbrook, USA) or PrioCHECK Ruminant Q Fever Ab Plate Kit (Thermo Fisher Scientific) according to the guidelines from the supplier, with a sample-to-positive percentage (S/P %) of ≥40 recorded as positive, values <30 as negative, and values between ≥30 and <40 considered inconclusive.

**Table 1:** Q fever–positive and –negative field sera pools from sheep.

Pool no.	Collection area	Collection year	Sheep flock information	Sera/pool	ELISA results (S/P%)^a^
1	NRW	2014	Q fever–positive, epidemiologically linked human cases	31	Positive (179.11)
2	BW	2016	Outbreak, epidemiologically linked human cases	10	Positive (198.83)
3	BW	2016	Outbreak, epidemiologically linked human cases	17	Positive (138.13)
4	RKS	2016	Outbreak, epidemiologically linked human cases	10	Positive (103.57)
5	NI	2017	Apparently healthy	11	Negative (0.03)
6	TH	2016	Apparently healthy	4	Negative (−0.17)
7	TH	2017	Apparently healthy, vaccinated	5	Negative (1.89)
8	NZ	n.d.	n.d.	n.d.	Negative (1.18)
9	NRW	2014	ELISA-negative animals from pool 1	2	Positive (149.61)

^a^Serum pools were tested using Q fever (*C. burnetii*) Ab Test (IDEXX Laboratories), and results were calculated as recommended by the supplier. An S/P% value of ≥40 indicated a positive result, values <30 a negative result, and values between ≥30 and <40 an inconclusive result. BW, Baden-Württemberg; NI, Lower Saxony; NRW, North Rhine-Westphalia; RKS, Republic of Kosovo; TH, Thuringia; NZ, New Zealand; Gibco sheep serum (Thermo Fisher Scientific).

### 2.4 Total protein extraction

Total protein extracts from axenic propagated *C. burnetii* isolates were prepared as described previously (Flores-Ramirez et al., 2014). Briefly, bacterial pellets (10 mg wet weight) were resuspended in lysis buffer (28 mM TRIS–HCl, 22 mM TRIS base, 200 mM DTT, 2% SDS) with protease inhibitor (Halt Protease Inhibitor, Thermo Fisher Scientific Inc., Waltham, MA, USA) and heat-inactivated (110 °C, 15 min) before exiting the BSL-3. Nucleic acids were degraded by adding 12.5 U Benzonase (Merck KGaA) for 12 h at 4 °C. Samples were centrifuged (12,500 × *g*, 20 min) and proteins precipitated from the supernatant by methanol–chloroform precipitation as described elsewhere (Wessel and Flugge, 1984). Protein pellets were air dried and resuspended in 300–500 μL isoelectric focusing (IEF) buffer (7 M urea, 2 M thiourea, 29 mM TRIS base, 1% ASB-14, 1% Triton X-100). For better solubility, a freeze–thaw step at −80 °C was included. Proteins were quantified using a 660-nm assay (Thermo Fisher Scientific Inc.).

### 2.5 Protein separation by two-dimensional (2D) gel electrophoresis

Non-linear (NL) 24-cm Immobiline DryStrips (pH 3–10, Merck KGaA) were rehydrated overnight with 300 μg *C. burnetii* protein extracts for colloidal Coomassie staining or 200 μg for Western blotting in 450 μL IEF buffer containing 1.2% Destreak Reagent (Merck KGaA), 1.5% immobilized pH gradient (IPG) buffer 3–10 NL (Merck KGaA), 1% protease inhibitor, 1 μL Lightning Red stain (SERVA Electrophoresis GmbH), and 0.005% bromophenol blue. Isoelectric focusing was conducted on an Ettan IPGphor III unit (GE Healthcare, Chicago, IL, USA) with the following steps: (i) gradient, 200 V, 0:30 h, 50 Vh; (ii) 500 V, 7:00 h, 3,000 Vh; (iii) gradient 1,000 V, 1:00 h, 800 Vh; (iv) gradient 5,000 V, 1:30 h, 4,500 Vh; 5 (v) final step 5,000 V, 5:19 h, 42,000 Vh. Focused strips were equilibrated in equilibration buffer (6 M urea, 4% SDS, 30% glycerol, 50 mM TRIS–HCl, pH 8.8) containing 65 mM DTT, followed by equilibration buffer containing 203 mM iodoacetamide each for 15 min at room temperature. The second dimension was carried out using 12.5% TRIS–HCl polyacrylamide gels in Ettan DALTsix chambers (GE Healthcare). Gels for spot harvesting were stained overnight with colloidal Coomassie (20% ethanol, 1.6% phosphoric acid, 8% ammonium sulfate, 1% Coomassie Brilliant Blue G250) and destained in distilled water.

### 2.6 Western blotting

Parallel gels were blotted onto polyvinylidene fluoride (PVDF) membrane (0.45 μm, Merck KGaA) using TRIS–glycine buffer (25 mM TRIS, 192 mM glycine) with 20% methanol and blocked in TBS-T buffer (20 mM TRIS, 150 mM NaCl, 0.05% Tween 20, pH 7.6) containing 10% non-fat dry milk at 4 °C overnight. Blots were incubated with Q fever–positive serum pool 1 (Table 1; 1:500) or Q fever–negative immune sera from sheep in TBS-T buffer containing 2% non-fat dry milk for 1 h at room temperature. After washing, bound antibodies were traced by alkaline phosphatase–conjugated anti-sheep IgG or anti-goat IgG antibodies (Merck KGaA, 1:5,000) in TBS-T buffer containing 2% non-fat dry milk for 1 h at room temperature. Substrate solution containing NBT (0.33 mg/mL) and BCIP (1.65 mg/mL) in alkaline phosphatase buffer (100 mM NaCl, 100 mM TRIS, 5 mM MgCl_2_ × 6H_2_O, pH 9.5) was added, and color development was allowed to proceed until visible bands appeared.

### 2.7 Spot matching and mass spectrometry analyses

Protein spots from colloidal Coomassie–stained gels and –immunostained membranes were matched with Delta2D 4.7 (DECODON Software UG, BioTechnikum Greifswald, Greifswald, Germany) and manually harvested. In-gel trypsin digestion was performed as described elsewhere with adaptations (Shevchenko et al., 2006; Flores-Ramirez et al., 2017). Briefly, spots were excised and destained with 50 mM NH_4_CO_3_ in 50% (v/v) ACN under gentle agitation. Subsequently, gel spots were dehydrated in 100% ACN, reduced in 10 mM DTT for 30 min at 50 °C, then further dehydrated and alkylated in 50 mM iodoacetamide for 30 min at room temperature in the dark while agitating. After additional dehydration and destaining, 10 ng/μL trypsin (Promega, Madison, WI, USA) in 10 mM NH_4_CO_3_ containing 10% ACN was added and left for 30 min at 4 °C, followed by incubation at 37 °C for an additional 14–16 h. Peptides were repeatedly extracted with 70% ACN containing 1% TFA, combined, and concentrated.

Peptides were analyzed by liquid chromatography–mass spectrometry (LC–MS/MS), using nanoAcquity UHPLC and Q-TOF Premier controlled by MassLynx software 4.1 (Waters, GmbH, Eschborn, Germany). Analytes were loaded onto the Symmetry C18 trap column (20 mm length, 180 μm diameter, 5 μm particle size, Waters). After 3 min of desalting/concentration by 1% acetonitrile containing 0.1% formic acid at a flow rate of 10 μL·min^−1^, samples were passed through a BEH130 C18 analytical column (200 mm length, 75 μm diameter, 1.7 μm particle size, Waters). For quick separation, a 20-min gradient of 5–40% acetonitrile with 0.1% formic acid was used, at a flow rate of 300 nL·min^−1^. The column outlet was connected to a PicoTip emitter (360 μm outer diameter, 20 μm inner diameter, 10 μm tip diameter, New Objective), and samples were nanosprayed (3.4 kV capillary voltage). Next, the spectra were recorded in the data-independent acquisition MSE (mass spectrometry efficiency) mode using MassLynx software 4.1 (Waters, GmbH, Eschborn, Germany). Alternate scans at low (4 eV) and high (20–40 eV ramp) collision energies were employed to obtain precursors and fragment masses in a single chromatographic run. Ions with 50–1,950 m·z^−1^ were detected in both channels, although quadrupole mass profile settings allowed efficient deflection of masses <400 m·z^−1^ in the low energy mode, enabling filtering of contaminating ions. The spectra acquisition scan rate was 0.8 s, with a 0.05-s inter-scan delay. The external mass calibrant Glu1-fibrinopeptide B (500 fmol·mL^−1^, Sigma-Aldrich) was infused through the reference line at a flow rate of 500 nL·min^−1^. It was measured every 30 s at 22 eV collision energy and used for the mass correction.

Data were processed using the ProteinLynx Global Server v. 3.0 (Waters, GmbH, Eschborn, Germany). For peak detection, the following thresholds were used: low energy of 140 counts, high energy of 30 counts, and intensity of 1,000 counts. Precursors and fragment ions were coupled using correlations of chromatographic elution profiles in low/high energy traces. The results were searched against *Coxiella burnetii* RSA 493 full proteome (UniProt accession: UP000002671; 1,812 sequences) downloaded from UniProt. Database searches were performed with carbamidomethylation of cysteine as a fixed modification, oxidation of methionine and deamidation of asparagine and glutamine as variable modifications, a maximum of one missed tryptic cleavage site, and automatic estimation of precursor and fragment mass errors from calibrant peaks. A 4% false discovery rate (FDR) against a randomized database, which was applied at the individual peptide level, was allowed. The accepted peptide score threshold was set to ≥6.00 to ensure a 95% confidence level. Additional quality matching criteria were as follows: (i) a minimum of three consecutive product ions per peptide; (ii) a minimum of seven total product ions per protein; (iii) a minimum of two peptides fitting the protein sequence.

### 2.8 Identification of clusters of orthologous groups

Amino acid sequences of all detected proteins were retrieved from the UniProt database for the *C. burnetii* reference strain Nine Mile phase I (RSA 493; UniProt accession: UP000002674). Deepnog v1.2.3 (Feldbauer et al., 2021) was used for Clusters of Orthologous Groups (COGs) assignment.

### 2.9 Epitope prediction and peptide synthesis

Amino acid (aa) sequences from immunogenic proteins described in the literature (Gerlach et al., 2017) or identified by 2D gel electrophoresis and Western blotting were retrieved from UniProt^1^ for the *C. burnetii* reference strain (RSA 493). Protein–protein Basic Local Alignment Search Tool (BLASTp) was used to identify *C. burnetii* aa sequences. All sequences were downloaded and aligned using Geneious Prime software (Geneious Prime 2021.0.1, Biomatters Ltd., Auckland, New Zealand) to identify protein regions, with a sequence identity of >87% for *C. burnetii* isolates and <50% for non-*Coxiella* bacteria.

B-cell epitope prediction for each protein was performed using IUPred3 (Rahman et al., 2015). Peptides of 24–30 aa length or of 18–21 aa length were selected and confirmed using BLASTp to exclude peptides with more than 50–75% aa identity to other ruminant pathogens.

Peptides were synthesized as 2 μmol crude extract with N-terminal biotin SGSG linker and a C-terminal amide group (peptides and elephants, Henningsdorf, Germany). Upon arrival, lyophilized peptides were dissolved in ultrapure water at a concentration of 0.7 μmol/mL, or if not soluble, in 50% dimethylsulfoxide (DMSO) with distilled water (0.35 μmol/mL) or DMSO (0.7 μmol/mL) and stored at −80 °C.

### 2.10 Fluorescent peptide ELISA

ELISAs were performed as previously described, with modifications (Rahman et al., 2015). Briefly, white flat-bottom 96-well plates with covalently linked streptavidin (Nunc; Fisher Scientific, USA) were used and initially washed one time with 400 μL and two times with 300 μL wash buffer (0.3 M NaCl, 20 mM TRIS–HCl (pH 7.5), 0.1% Tween 20, 0.001% benzalkonium chloride, pH 7.5) for 15 min at room temperature. Peptides were diluted in assay diluent (0.2 M NaCl, 20 mM TRIS–HCl (pH 7.5), 10% chicken serum (Merck KGaA), 0.5% PEG, 0.1% Tween 20, 0.004% benzalkonium chloride, pH 7.5) to final concentrations of 0.75–25 pmol. The binding of peptides was carried out by adding 100 μL peptide solution to each well for 30 min at room temperature with agitation (60 rpm). Unbound peptides were removed by five washes with 300 μL wash buffer, and non-specific binding was blocked with 300 μL blocking buffer per well [0.2 M NaCl, 20 mM TRIS–HCl (pH 7.5), 10% chicken serum, 1% PEG, 0.004% benzalkonium chloride, pH 7.5] for 30 min at room temperature. Serum pools were diluted 1:100 in assay diluent; 100 μL per well was added and incubated for 1 h with agitation (60 rpm). The plate was washed five times with 300 μL of wash buffer, and 100 μL of peroxidase-conjugated anti-goat/sheep IgG monoclonal antibody (Merck KGaA), diluted 1:8,000 in assay diluent, was added for 30 min at room temperature. After five washes with 300 μL of wash buffer, the reaction was traced with QuantaBlu Kit (Thermo Fisher Scientific) as described by the supplier. The relative fluorescence units (RFUs) of each well were recorded, with excitation and emission wavelengths set at 325 nm and 420 nm, respectively.

All serum pools and single serum samples were tested with peptide-coated and -uncoated wells for background correction. A 150% correction factor relative to the signal intensity from the uncoated wells was subtracted from the peptide-coated wells for each serum. Signals above the background indicated peptide reactivity for the respective serum pool or serum (Rahman et al., 2015). Peptides that reacted with Q fever–positive sera but not with negative sera were considered specific peptide epitopes. Pairwise comparison was performed using GraphPad Prism 10, Version 10.6.1, using the two-way ANOVA tool. Reactivity with all 15 peptide epitopes from serum pools derived from true-positive sheep flocks (pools 1–4) or vaccinated animals (pool 9) was compared with serum pools derived from true-negative sheep flocks (pools 5–8) (Supplementary Table S3). *p*-value ≤ 0.05 were considered statistically significant.

### 2.11 Data analysis

Data were analyzed using Excel (Microsoft Office Professional Plus Version 1808) and GraphPad Prism 10. Receiver operating characteristic (ROC) curves were generated with MedCalc Version 14.8.1 (MedCalc Software Ltd., Ostend, Belgium). For a six-peptide combination, a composite score was calculated for each serum sample with the corresponding Youden Index set as the individual cutoff. This score, ranging from 0 to 6, was used for ROC analysis in comparison with the commercial ELISA. Individual serum samples were classified as positive based on ELISA results (S/P% > 40), qPCR confirmation of the infection, and/or herd status. Vaccinated animals and incomplete data sets were excluded. Sensitivity (Se) was calculated as TP/(TP + FN) and specificity (Sp) as TN/(TN + FP) with TP, true positive; TN, true negative; FP, false positive; and FN, false negative for selected peptide panels. Youden Index was calculated as follows: Se + Sp - 1 (Schisterman et al., 2008). A Youden Index of 1 describes a perfect test with no false-positive or false-negative results, a value above 0.5 describes an acceptable test, and 0 indicates a test with no discriminatory power.

## 3 Results

### 3.1 Identification of *C. burnetii* seroreactive proteins

**Figure 1:**
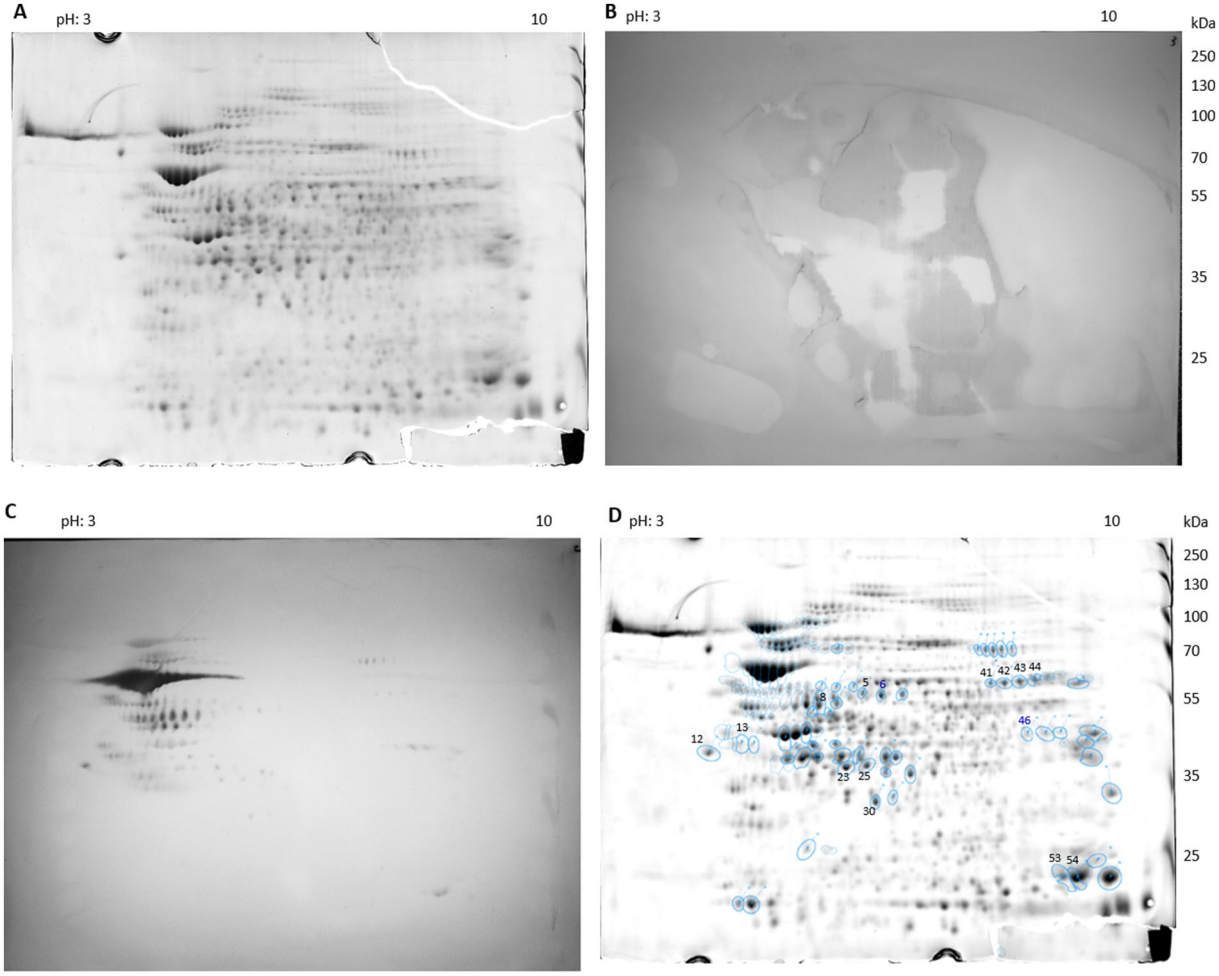
Representative proteomic map of *C. burnetii* isolate 26QC00015. **(A)** Total proteins separated by 2D electrophoresis and stained with colloidal Coomassie. **(B,C)** Corresponding immunoblots probed with **(B)** Q fever–negative serum from sheep (control) and **(C)** pool 1 (Table 1) of Q fever–positive serum from sheep. **(D)** Computational spot matching performed with Delta2D 4.7 (Decodon). Numbered and circled spots were selected for identification based on a normalized volume threshold (immunostained spots > 0.1 and Coomassie-stained spots > 0.2). Newly identified immunoreactive proteins are labeled in blue: CBU0462 (PdhC, spot 6), a dihydrolipoamide acetyltransferase component of the pyruvate dehydrogenase complex, and CBU0974 (spot 46), an acetyl-CoA acetyltransferase. Additional Western blots and overlays are provided in Supplementary Figures S1–S4.

A total of 156 seroreactive proteins were detected from *C. burnetii* 26QC00015 protein extracts using 2D gel electrophoresis and Western blotting with a pool of sheep sera collected during a Q fever outbreak (pool 1, Table 1). Representative images of the colloidal Coomassie–stained gel, Western blotting, and spot matching are displayed in Figure 1. Additional images of Coomassie-stained gels, Western blot spot matching, and identified immunoreactive proteins are provided in Supplementary Figures S1–S4 and Supplementary Table S4.

**Figure 2:**
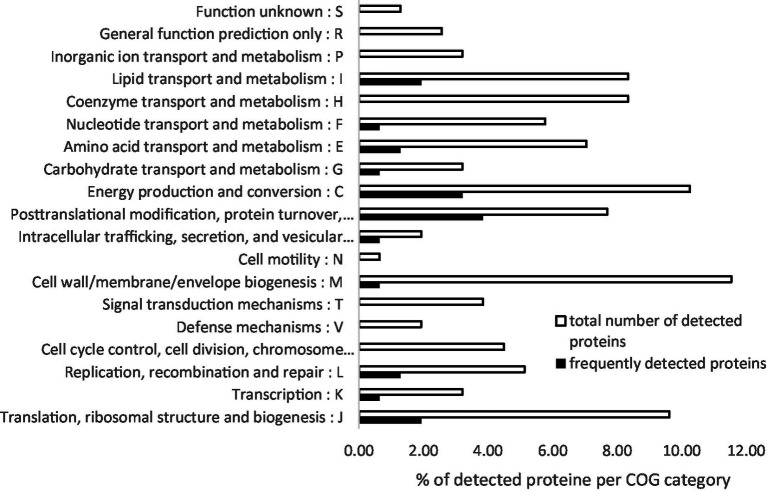
Functional categorization (COG) of *C. burnetii* seroreactive proteins. Proteins were identified from strain 26QC00015 using 2D gel electrophoresis and western blotting with Q fever–positive sera. The chart shows the distribution of all identified proteins and the most frequently detected proteins across COG functional categories. Frequently detected proteins were defined as those identified in ≥4 independent western blot analyses using different Q fever–positive sera.

Detected proteins were mostly involved in general metabolism (46.15%), falling into COG categories for energy production and conversion (COG C), coenzyme (COG H), lipid (COG I), and amino acid (COG E) transport and metabolism (Figure 2). Another 32.05% were involved in cellular processes and signaling, while 17.95% were associated with information storage and processing. Notably, several proteins belonged to the cell wall/membrane/envelope biogenesis (COG M, 11.54%), including the potential virulence factor enhanced entry protein EnhA.1. Of the most frequently detected and previously described seroreactive proteins, eight highly conserved cytosolic proteins involved in metabolism, cellular processing, and signaling, as well as information storage and processing, were detected. This includes the well-characterized membrane-bound or outer membrane–bound proteins Com1 and CBU_0482, as well as the secreted protein and potential virulence factor Mip.

The majority of the detected proteins (*n =* 130) were identified in only one or two experiments, while 26 proteins were reproducibly detected three or four times and are listed in Table 2. Most of these frequently detected proteins were involved in general metabolism (7.69%), primarily in energy production and conversion (COG C) and lipid transport and metabolism (COG I). Other frequently detected proteins were associated with information storage and processing (5.13%) and cellular processes and signaling (3.85%) (Figure 2). Only two membrane-bound or periplasmic proteins, CBU_1538 and TolB, were identified as seroreactive in this study.

**Table 2:** Seroreactive proteins identified from *C. burnetii* isolate 26QC00015 (GenBank assembly accession GCF_000007765.2, RSA 493 reference genome).

Locus tag	Protein name	Protein function^a^	Subcellular location^a^	Detection frequency	References
CBU_0482		Arginine-binding protein	Membrane	3	Zhang and Samuel (2003) and Deringer et al. (2011)
CBU_0495	FabG	3-Oxoacyl-[acyl-carrier protein] reductase	Cytosol	4	Deringer et al. (2011), Jiao et al. (2014), and Flores-Ramirez et al. (2017)
CBU_0497	FabF	3-Oxoacyl-[acyl-carrier-protein] synthase	Cytosol	3	Coleman et al. (2007), Vigil et al. (2010), and Xiong et al. (2012)
CBU_0572	pepB	Cytosol aminopeptidase	Cytosol	4	Sekeyova et al. (2009), Vigil et al. (2010), Papadioti et al. (2012), and Flores-Ramirez et al. (2017)
CBU_0630	Mip	Peptidyl-prolyl cis–trans isomerase Mip	Secreted	3	Zhang and Samuel (2003), Sekeyova et al. (2009), Sekeyova et al. (2010), Vigil et al. (2010), Chen et al. (2011), Deringer et al. (2011), Vigil et al. (2011), Papadioti et al. (2012), Wang et al. (2013), Jiao et al. (2014), Xiong et al. (2014), and Gerlach et al. (2017)
CBU_1054	RecA	DNA repair	Cytosol	4	Chao et al. (2005)
CBU_1200	Icd	Isocitrate dehydrogenase	n.d.	4	Coleman et al. (2007)
CBU_1398	SucB	Dihydrolipoyllysine-residue succinyltransferase component of 2-oxoglutarate dehydrogenase complex	Cytosol	4	Zhang and Samuel (2003), Beare et al. (2008), Deringer et al. (2011), Vigil et al. (2011), Xiong et al. (2012), and Flores-Ramirez et al. (2017)
CBU_1718	GroL	60 kDa chaperonin	Cytosol	4	Chao et al. (2005), Sekeyova et al. (2009), Vigil et al. (2010), Deringer et al. (2011), Vigil et al. (2011), Papadioti et al. (2012), Xiong et al. (2012), Wang et al. (2013), Jiao et al. (2014), Xiong et al. (2014), Flores-Ramirez et al. (2017), and Xiong et al. (2017)
CBU_1910	Com1	Outer membrane protein	Outer membrane	4	Zhang and Samuel (2003), Chao et al. (2005), Beare et al. (2008), Sekeyova et al. (2009), Sekeyova et al. (2010), Chen et al. (2011), Deringer et al. (2011), Vigil et al. (2011), Papadioti et al. (2012), Xiong et al. (2012), Wang et al. (2013), Jiao et al. (2014), Xiong et al. (2014), Flores-Ramirez et al. (2017), and Xiong et al. (2017)
CBU_2012	Hslu	ATP-dependent protease ATPase subunit	Cytosol	4	Flores-Ramirez et al. (2017)
CBU_2086	Rho	Transcription termination factor	Cytosol	4	Flores-Ramirez et al. (2017)
CBU_0039	PrlC	Oligopeptidase A	Cytosol	3	This study
CBU_0090	TolB	Protein TolB	Periplasm	3	This study
CBU_0221b	TufA	Elongation factor Tu	Cytosol	4	This study
CBU_0462	PdhC	Acetyltransferase component of the pyruvate dehydrogenase complex	Cytosol	3	This study
CBU_0463	LpdA	Dihydrolipoyl dehydrogenase	n.d.	4	This study
CBU_0521	MtaD	5-Methylthioadenosine/S-adenosylhomocysteine deaminase	n.d.	4	This study
CBU_0676	GalE	UDP-glucose 4-epimerase	n.d.	4	This study
CBU_0974		Acetyl-CoA acetyltransferase	n.d.	3	This study
CBU_0988	Ung	Uracil-DNA glycosylase	Cytosol	3	This study
CBU_1273	Pfp	Pyrophosphate-fructose 6-phosphate 1-phosphotransferase	Cytosol	3	This study
CBU_1326	ThrS	Threonine-tRNA ligase	Cytosol	3	This study
CBU_1397	SucC	Succinate-CoA ligase	Cytosol	3	This study
CBU_1475	GatB	Aspartyl/glutamyl-tRNA(Asn/Gln) amidotransferase subunit B	n.d.	3	This study
CBU_1538	ClpP	Carboxy-terminal processing protease	Outer membrane-bounded periplasmic space	3	This study

^a^UniProt (https://www.uniprot.org/); n.d., not determined. The table lists the identified proteins with their predicted function, subcellular location, detection frequency, and supporting references.

### 3.2 Prediction and validation of peptide epitopes

Based on a previously published literature review of *C. burnetii* immunoreactive proteins, nine proteins were selected for epitope prediction based on the highest publication frequency and the highest number of sera used in these studies (Gerlach et al., 2017). These included outer membrane proteins Com1 (CBU1910) and OmpH (CBU0612), periplasmic protein YbgF (CBU_0092), peripheral cell membrane protein DnaK (CBU_1290), cytosolic proteins GroEL (CBU_1718), Tuf-2 (CBU_0236), and SucB (CBU_1398), secreted protein Mip (CBU_0630), and one protein of unknown function and location (CBU_0937). Subsequently, based on detection frequency in 2D gel electrophoresis and Western blot analyses, four additional proteins were selected: the peripheral cell membrane protein Coq7 (CBU_1870), the cytosolic protein ScpB (CBU_1060), and two proteins of unknown location, CBU_0974 and CBU_0943 (Supplementary Table S4).

From these 13 proteins, 30 peptides were selected for synthesis (Supplementary Table S5). The selection criteria were a disordered score (IUpred3) > 0.5 and high sequence specificity: >87% identity to published *C. burnetii* isolates and ≤87% to *Coxiella*-like endosymbionts. To minimize cross-reactivity, we confirmed that these peptides shared <73% sequence identity with homologous proteins from other common bacterial pathogens, including *Staphylococcus* spp., *Nocardia* spp., *Mycobacterium* spp., *Escherichia* spp., *Leptospira* spp., *Salmonella* spp., *Enterococcus* spp., and *Brucella* spp.

### 3.3 Reactivity of peptides using Q fever–positive and –negative sheep serum pools

All 30 peptides were screened in triplicate against nine field serum pools, comprising 90 individual serum samples from sheep flocks positive or negative for Q fever, as well as from vaccinated animals. Notably, serum pool 9 consisted of two individual samples (14QC1941 and 14QC1945) that tested negative in a commercial ELISA when analyzed separately. However, when combined into this pool, they yielded a positive signal (Table 1, Supplementary Table S2). These samples were originally collected during an outbreak in North Rhine-Westphalia (Pool 1). A reaction was considered positive if it exceeded 150% of the background signal in at least two replicates.

Fifteen peptides were excluded from further analysis due to a lack of reactivity or inconsistent (i.e, non-reproducible reactivity across independent experiments) results (data not shown). The remaining 15 peptides reacted with at least one Q fever–positive serum pool and showed no reaction with Q fever–negative serum pools (Table 3; Supplementary Figure S5 and Supplementary Table S2). The majority of these peptides reacted with two (*n =* 3) or three (*n =* 7) positive serum pools. Only peptide no. 22 reacted with all five positive serum pools. No peptide reacted with the negative serum pools, except peptide 20, which showed a single positive reaction in one of three independent experiments. The serum pool from vaccinated animals reacted with five peptides, all of which were also recognized by Q fever–positive serum pools.

**Table 3:** Seroreactivity of peptides with Q fever–positive and –negative sheep serum pools.

Predicted *C. burnetii* peptide epitopes		Reactivity^a^ with sheep serum pools, number of positive reactions/number of repeated tests								
		Q fever–positive					Q fever–negative			Vaccinated
Peptide-epitope no.	Protein name	1	2	3	4	9	5	6	8	7
2	Com1	0/3	0/3	3/3	0/3	0/3	0/3	0/3	0/3	0/3
4	GroEL	3/3	3/3	0/3	0/3	0/3	0/3	0/3	0/3	3/3
5	Tuf-2	3/3	3/3	3/3	0/3	1/3	0/3	0/3	0/3	0/3
6	OmpH	3/3	3/3	0/3	0/3	3/3	0/3	0/3	0/3	0/3
7	OmpH	3/3	3/3	0/3	0/3	0/3	0/3	0/3	0/3	0/3
8	OmpH	2/3	3/3	0/3	2/3	0/3	0/3	0/3	0/3	0/3
11	YbgF	3/3	2/3	3/3	0/3	3/3	0/3	0/3	0/3	2/3
13	YbgF	3/3	3/3	3/3	0/3	1/3	0/3	0/3	0/3	1/3
16	DnaK	0/3	3/3	0/3	0/3	0/3	0/3	0/3	0/3	0/3
17	DnaK	3/3	1/3	0/3	3/3	3/3	0/3	0/3	0/3	0/3
19	SucB	0/3	0/3	3/3	0/3	0/3	0/3	0/3	0/3	0/3
20	SucB	1/3	3/3	0/3	0/3	0/3	1/3	0/3	0/3	0/3
21	SucB	3/3	3/3	3/3	0/3	0/3	0/3	0/3	0/3	2/3
22	Mip	3/3	2/3	3/3	3/3	3/3	0/3	0/3	0/3	3/3
25	Coq7	3/3	1/3	0/3	0/3	0/3	0/3	0/3	0/3	3/3

^a^Reactivity of 150% over the background was evaluated as positive. Values represent results from three independent experiments.

### 3.4 Evaluation of peptide epitopes with serum samples of known Q fever status

To further evaluate the diagnostic potential of selected peptides for applications requiring high sensitivity (e.g., prevalence studies) or high specificity (confirmatory testing of a suspected or clinical case), they were tested against individual serum samples from sheep of known Q fever status. Of the 90 sera used to create the initial pools, 79 were selected for ELISA analysis. The remaining 11 samples were unavailable due to their insufficient volume.

All serum samples were grouped as originating from “infected,” “uninfected,” or “vaccinated” animals.

Reactivity with peptides was detected in all groups. In the infected group, all serum samples (*n =* 60) showed reactivity to at least one peptide; in some cases, up to 10 peptides were recognized. Individual serum samples (11/13) from the vaccinated group, which tested negative in pool analysis, showed reactivity with up to seven peptides (see also Supplementary Table S1).

**Figure 3:**
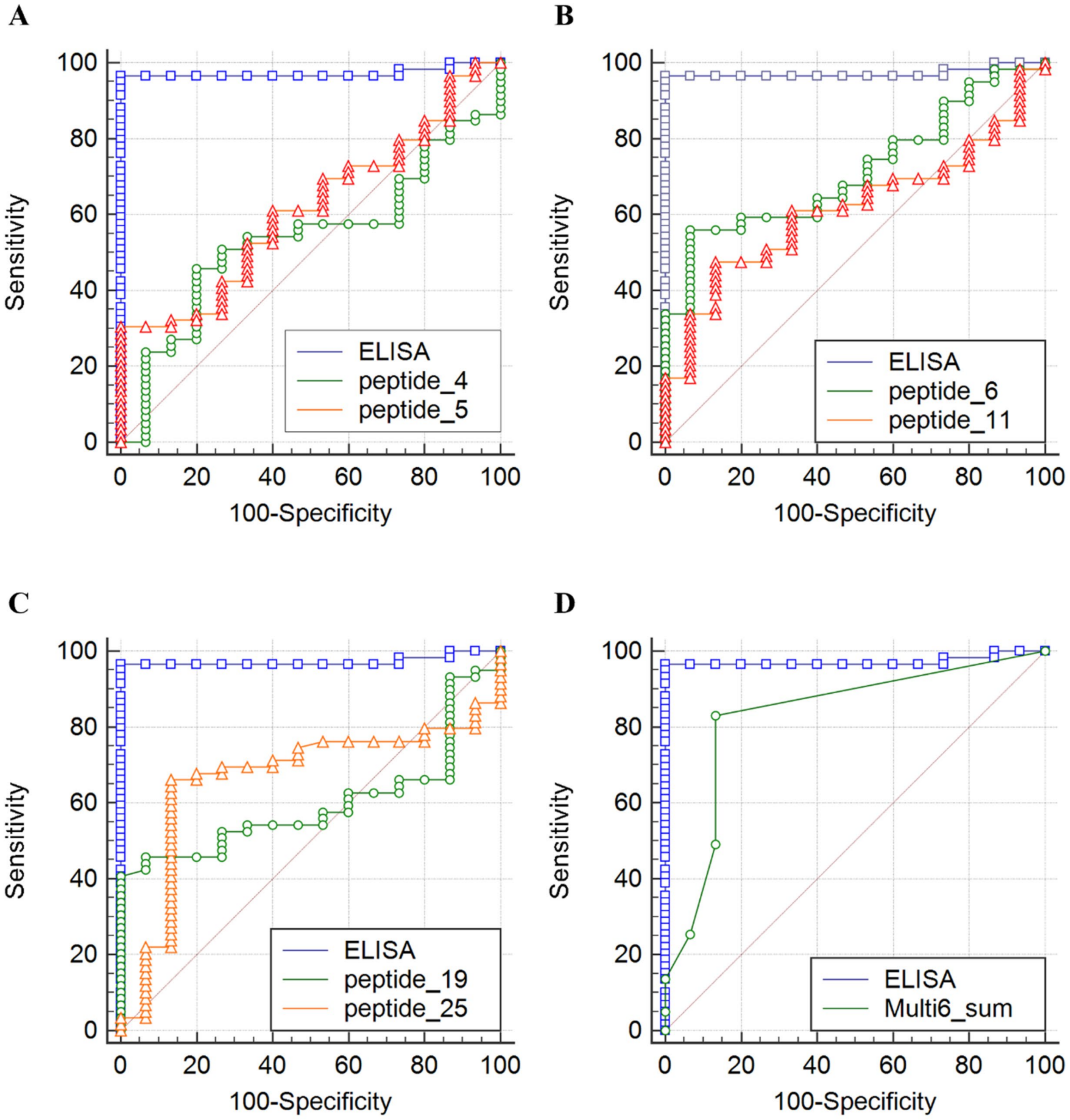
Diagnostic performance of individual peptide antigens **(A-C)** and a six-peptide combination (**D**; Multi6_sum) in comparison with a commercial ELISA (**D**; ELISA). Receiver operating characteristic (ROC) curves illustrating the diagnostic performance of six individual peptide antigens and their combination (Multi6_sum) using 74 individual sheep serum samples. Samples were classified as Q fever–positive based on commercial ELISA results (S/P% > 40), qPCR confirmation of the infection, and/or herd status. Score for the combination of six peptides (4, 5, 6, 11, 19, and 25) reflects the number of reactive peptides per serum sample (range 0–6). Vaccinated animals and samples with missing values were excluded from this analysis. Sensitivity (%) is plotted against 100 – specificity (%) to evaluate the ability of each peptide to discriminate between Q fever–positive and –negative samples.

To determine the diagnostic potential of each peptide, ROC curves were generated and compared with the commercial ELISA. In Figures 3A–C, the ROC curves are displayed for six individual peptides with the highest discriminatory power. The AUC values for individual peptides ranged between 0.5 and 0.7, indicating poor to fair discriminatory power (Supplementary Figure S6). In contrast, the commercial ELISA shows near-perfect discrimination, likely caused by the selection of true-positive and true-negative serum samples.

To evaluate whether peptide combinations improve the diagnostic performance, Se and Sp were calculated for all possible peptide combinations using the rule that a sample was considered “overall positive” if it reacted to at least one of the selected peptides (Supplementary Table S6). The highest Youden Index of 0.55, indicating an acceptable discriminatory power, was achieved for peptide combinations comprising six, seven, or eight peptides (Table 4). The generated ROC analysis (Figure 3D) reveals that by using a combination of peptides, the discriminatory power improved. However, the plateau of the curve indicates that using more than six peptides provides limited further benefit.

**Table 4:** Diagnostic accuracy of selected peptide combinations.

Number of peptides	Peptide combination	Se^1^	Sp^2^	J^3^
6	4, 5, 6, 11, 19, 25	0.8	0.75	0.55
6	4, 5, 6, 11, 20, 25	0.8	0.75	0.55
7	4, 5, 6, 11, 16, 19, 25	0.8	0.75	0.55
7	4, 5, 6, 11, 16, 20, 25	0.8	0.75	0.55
7	4, 5, 6, 11, 19, 20, 25	0.8	0.75	0.55
8	4, 5, 6, 11, 16, 19, 20, 25	0.8	0.75	0.55

^1^Sensitivity is the proportion of true positives (TP) among all samples derived from Q fever–positive animals. It reflects the ability of the test to correctly identify infected individuals. ^2^Specificity is the proportion of true negatives (TN) among all derived from Q fever–negative animals. It reflects the ability of the test to correctly identify non-infected individuals. ^3^Youden Index serves as a global measure of test accuracy. It ranges from 0 (no diagnostic value) to 1 (perfect test). A value above 0.5 is generally considered acceptable. Sensitivity, specificity, and Youden Index were calculated for different peptide combinations.

Taken together, these results show that the selected peptides allowed the detection of antibodies specific to *C. burnetii* in serum samples of both infected and vaccinated animals. Although reactivity was observed in serum samples from the uninfected group, the overall signal intensity and number of reactive peptides were lower than in the infected group. The best-performing combination reached a Se of 80% and Sp of 75%. This indicates that the approach attempted here to select and use peptides for Q fever diagnosis is promising; however, achieving robust diagnostic performance will require epitope prediction and validation on a much larger scale.

## 4 Discussion

The diagnosis of Q fever remains challenging due to limitations in both direct pathogen detection and serological assays. Although serodiagnostic tests like the widely applied ELISA or the indirect IFA for human Q fever diagnostics are standard, they rely heavily on antigens derived from inactivated whole *C. burnetii* cells. This necessitates biosafety level 3 containment for antigen production and results in a complex antigenic mixture that can lead to cross-reactions. This study was initiated to overcome these limitations by moving toward a defined antigen approach. The identification of specific, immunodominant peptide epitopes could form the basis of a safer, more standardized, and highly specific diagnostic assay, eliminating the need to culture the pathogenic agent.

As a basis for *in silico* prediction of *C. burnetii*-specific peptide epitopes, immunodominant proteins were selected from proteome analyses of a field isolate with sera derived from sheep. In addition, literature that used sera from human, mouse, and guinea pig was screened for such epitopes (Vigil et al., 2010; van Schaik et al., 2013). A large proportion (51/159) of the experimentally identified proteins had been previously reported (Vigil et al., 2010). Interestingly, 12 of the 26 most frequently detected proteins are known antigenic proteins such as Com1 and Mip, reported 17 or 6 times, respectively, from studies using human, mouse, and guinea pig serum samples (Gerlach et al., 2017). This could indicate that the majority of immune targets are common between different host species. However, the newly and frequently identified proteins expand the known immunoproteome of *C. burnetii* and may provide new insights into the host–pathogen interaction during Q fever.

Notably, not all protein spots detected by Coomassie staining were detectable in the corresponding Western blot analyses. This discrepancy likely reflects technical limitations as well as differences in protein abundance or epitope accessibility.

Many of the frequently detected proteins were associated with central metabolism, similar to previous proteomic studies of *C. burnetii* and other pathogenic bacteria, where housekeeping proteins often contribute to the serological response (Deringer et al., 2011; Xiong et al., 2012; Corona et al., 2013; Flores-Ramirez et al., 2017; Misra et al., 2018). Although they are not surface exposed, they may be released during bacterial turnover or lysis and subsequently elicit an antibody response (Dosztanyi et al., 2005; Papadioti et al., 2012). The repeated identification of these highly conserved cytosolic enzymes suggests that they may play a role as “immunodominant” antigens, despite lacking classical surface localization.

These highly conserved proteins may contribute to cross-reactivities in diagnostic tests based on protein extracts or whole-cell lysates, lacking a defined set of antigens. To overcome these hurdles and to increase the sensitivity and specificity of the currently available ELISA tests for Q fever diagnostics in veterinary medicine, peptides resembling B-cell epitopes were predicted from the here-identified proteins as well as from well-known published antigenic proteins of *C. burnetii*. Previous peptide-based studies, such as those for *Chlamydia* spp., showed high serological accuracy using a similar approach for selection of epitopes, reinforcing the potential of this strategy (Rahman et al., 2015; Sachse et al., 2018). Several proteins revealed nearly 100% sequence identity between available *C. burnetii* isolates and enabled easy selection of peptide epitopes, whereas other proteins showed extended conserved areas such as GroEL (CBU_1718), Tuf-2 (CBU_0236), or DnaK (CBU_1290). These conserved proteins hindered the selection of specific protein areas, and epitopes derived from these proteins had identities of more than 50% with non-*Coxiella* bacteria, which may result in cross-reactions (Rahman et al., 2015). However, 15 of the 30 synthesized peptide epitopes showed high specificity when screened with field sera pools from Q fever–positive and –negative ruminant herds as well as from vaccinated animals.

The strongest candidate was peptide 22 (Mip), which reacted with all Q fever–positive serum pools and 23 of 57 individual positive serum samples, consistent with its role as a virulence factor (Debowski et al., 2023), and advocates for its selection as a potential target for vaccine development. Several other peptides, including those derived from GroEL, OmpH, YbgF, and SucB, also displayed a broad reactivity. Unexpectedly, the sera from animals classified as “uninfected” showed a high reactivity with up to seven peptides, although pooled samples from this group tested negative. This might be caused by non-specific cross-reactivity due to conserved bacterial epitopes, e.g., with *Chlamydia* spp., *Legionella* spp., and *Bartonella* spp. or *Escherichia coli*, unknown exposure to *C. burnetii,* or technical variation of the assay (La Scola and Raoult, 1996; Musso and Raoult, 1997; Lukacova et al., 1999; Rahman Kh et al., 2016). The latter might be explained by the sequence similarity of metabolic proteins such as SucB between *C. burnetii* and *E. coli*. In addition, the presence of *E. coli* strains in the gut microbiome of ruminants could contribute to low-level antibody responses (La Scola and Raoult, 1996; Seshadri et al., 2003). Contrary to previous studies on peptide-based diagnostics of *Chlamydia* spp., individual predicted peptides showed limited diagnostic performance, with AUC values close to 0.5–0.7 (Sachse et al., 2018; Rahman and Kaltenboeck, 2019, 2021). However, when multiple peptides were combined, diagnostic performance improved slightly, reaching a sensitivity of 80% and a specificity of 75%. This supports the concept that multi-epitope approaches are necessary to address the heterogeneity of the host immune response during infection.

Although this study focused on sheep, several of the immunodominant proteins identified are highly conserved among *Coxiella burnetii* isolates from different ruminant species and humans. The peptide-based approach used here targets short, defined linear B-cell epitopes representing only a small and specific fraction of the full-length protein. In contrast, whole proteins may comprise multiple epitopes that can be differentially recognized depending on host species and immune status. By using selected peptide epitopes rather than entire proteins, this approach may reduce complexity and improve specificity, suggesting potential applicability of the identified peptides across different host species.

A drawback of the here-presented study is that a small-scale approach was used to identify immunoreactive proteins using the literature and wet-lab proteomic analysis of a single *C. burnetii* field isolate. More recent studies use bioinformatic tools or deep neural networks for epitope prediction on a much larger scale, reaching AUCs of 0.67 ± 0.7 (Collatz et al., 2021). High-throughput peptide microarrays can provide quantitative data on epitope immunodominance and help identify strain-specific immune responses. However, as shown for *Chlamydia* spp. (Rahman et al., 2015; Sachse et al., 2018), such studies need to be performed on a much broader scale to reliably define truly specific peptide epitopes. Regarding the epitope structure, all of the selected peptide candidates were linear, and although conformational epitopes may contribute to antibody recognition, Rahman Kh et al. (2016) demonstrated that linear epitopes are often sufficient for species-specific serology, reducing the need to include complex conformational targets (Rahman Kh et al., 2016).

Furthermore, peptide selection was initially performed using pooled sera from defined Q fever–positive and –negative sheep flocks and subsequently evaluated using individual serum samples of the same origin. Therefore, the here-reported diagnostic performance may be positively biased due to this partial overlap. However, testing individual sera represents a more stringent approach compared to pooled sera, as it captures individual and heterogeneous antibody responses. Validation using an unrelated panel of serum samples would further improve the assessment of peptide performance.

Although the serum collection comprised samples of different origins, it was restricted to nine pools for initial screening and 79 individual sera for validation. This limited sample size reduces statistical power and does not capture the full diversity of *C. burnetii* circulating in different regions and host species. Moreover, each serum sample was tested only once, which limits reproducibility. In addition, no panels from animals with confirmed infections with potentially cross-reactive pathogens (e.g., *Chlamydia*, *Brucella*, *Legionella*, *Bartonella*) were included to assess analytical specificity (La Scola and Raoult, 1996; Musso and Raoult, 1997; Lukacova et al., 1999; Shapira et al., 2021).

Overall, a systematic proteomic screening approach combined with *in silico* epitope prediction can successfully be applied to identify peptide epitopes for serological diagnostics of Q fever. Although single peptides showed limited diagnostic value, multi-peptide combinations performed better. Nevertheless, larger validation studies with extended serum collections are required to refine peptide selection and confirm diagnostic accuracy.

In conclusion, this study underscores the viability of a defined peptide-based serodiagnostic approach for Q fever, which offers a safer and more standardized alternative to assays requiring cultivation of the pathogenic agent. Although the diagnostic performance of individual peptides was limited, our findings provide crucial proof-of-concept that multi-epitope combinations can capture the heterogeneous antibody response to *C. burnetii* infection. The immunoproteome data and the initial panel of candidate peptides presented in this study serve as a valuable resource and a foundational step toward the development of a robust, next-generation diagnostic test. Future efforts, employing high-throughput epitope mapping and validation against larger, more diverse serum panels, including samples from animals infected with cross-reactive pathogens, are now warranted to translate this promising strategy into a clinically reliable tool.

## Data Availability

The data presented in this study are publicly available. The data can be found at: https://zenodo.org/records/17669534.

## Footnotes

1
https://www.uniprot.org/
