# Supplementary material for: B-cell peptide epitopes as diagnostic targets for Q fever in sheep
Source: Front Microbiol. 2026 Feb 17;17:1751544. doi: 10.3389/fmicb.2026.1751544 (PMC12953093; doi:10.3389/fmicb.2026.1751544)
Supplement: Supplementary file 1 [file Data_Sheet_1.pdf]

Supplementary Material

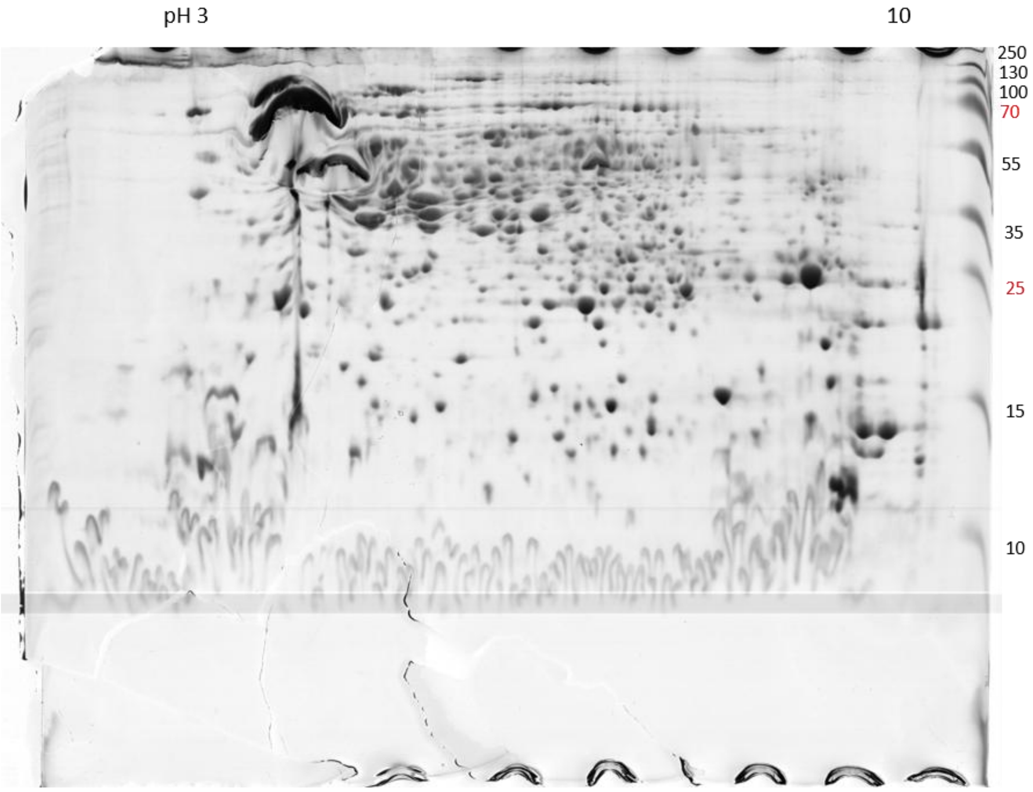

1A

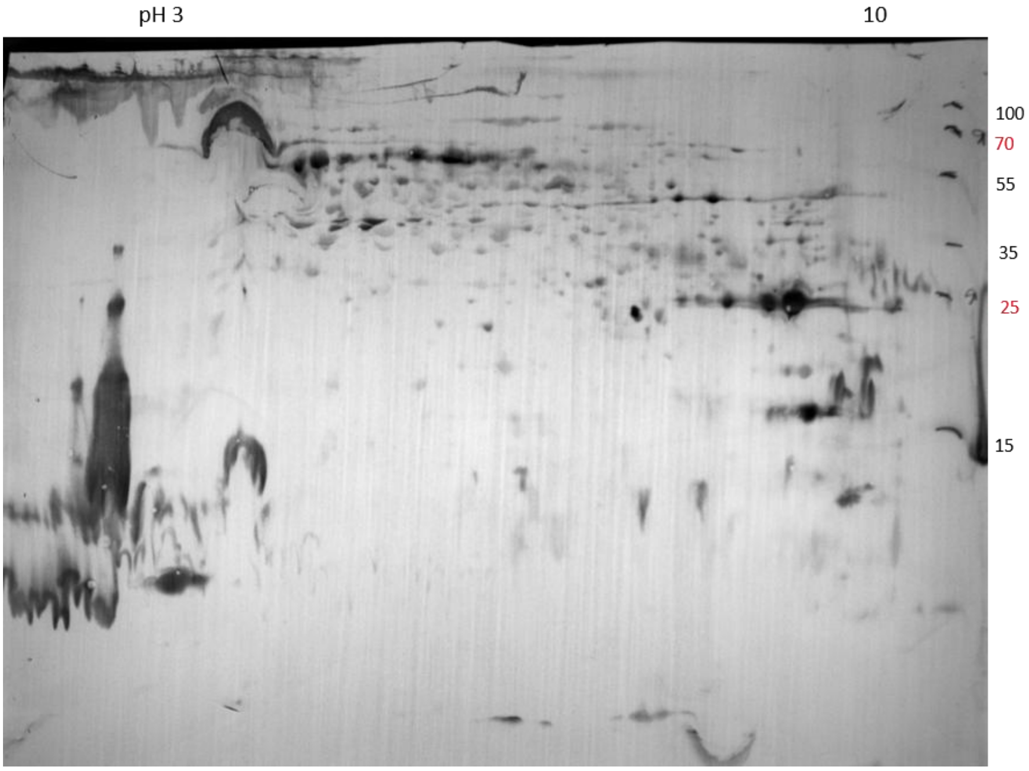

1B

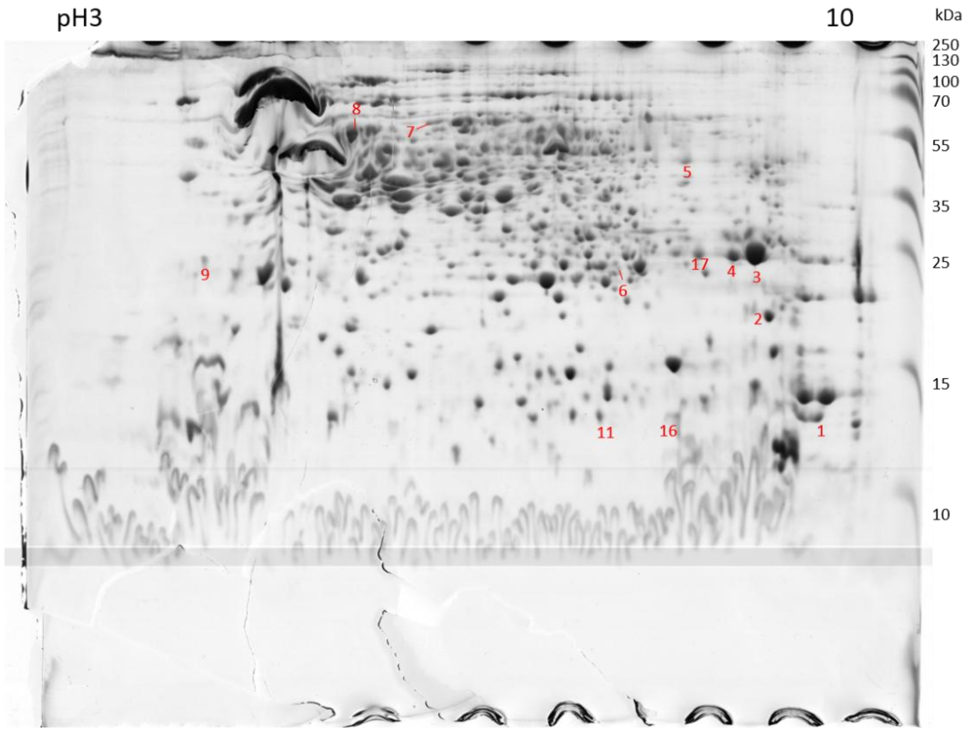
pH3
10
kDa
250
130
100
70
55
35
25
15
10
8
7
5
9
17
4
3
6
2
11
16
1

1C

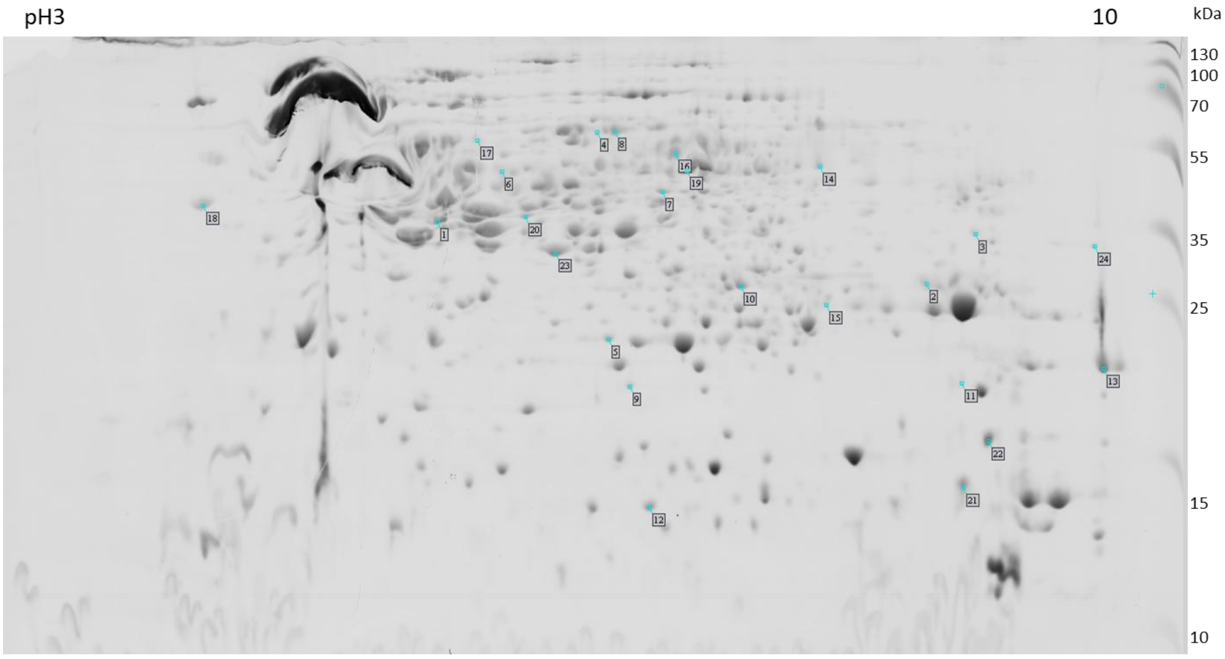
pH3
10
kDa
130
100
70
55
35
25
15
10

1D

Figure S1: Two-dimensional gel electrophoresis of protein extracts of *C. burneii* isolate 26QC00015 **(A)** stained with colloidal Coomassie and **(B)** Western blotting with pool of Q fever-positive serum from sheep pool 1 and **(C and D)** spot matching using Delta2D 4.7 (Decodon) and spots with distinct normalized volumes (immunostained spots > 0.1 and Coomassie stained spots > 0.2) were selected for protein identification (numbered, circled spots). Results of protein identification is displayed in supplemental table 2.

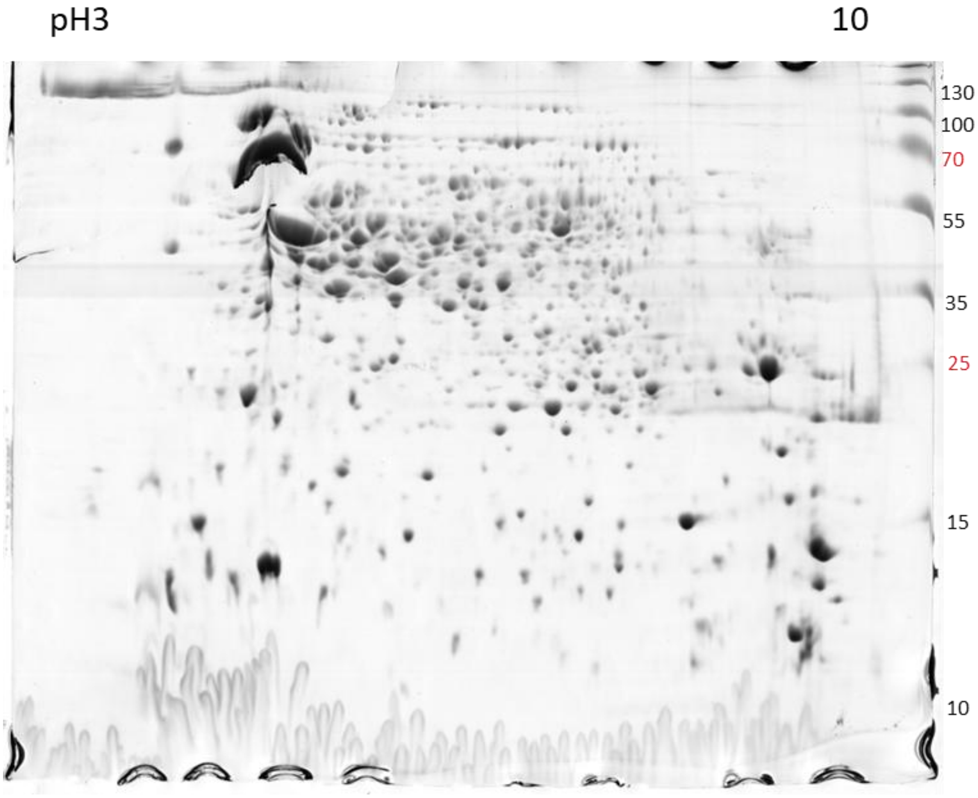

2A

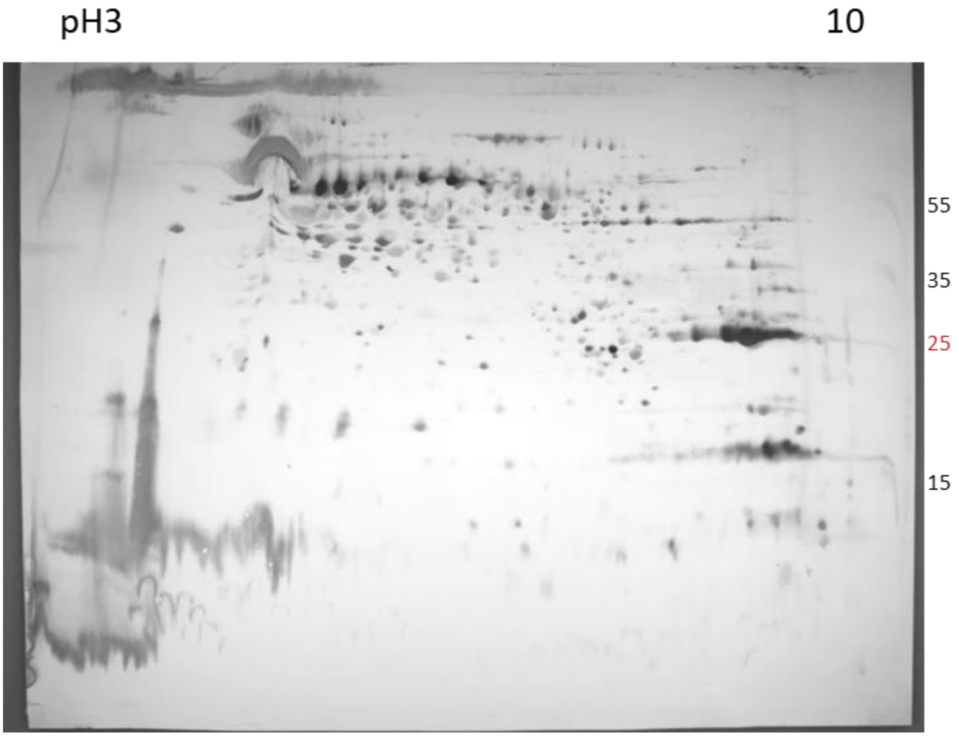

2B

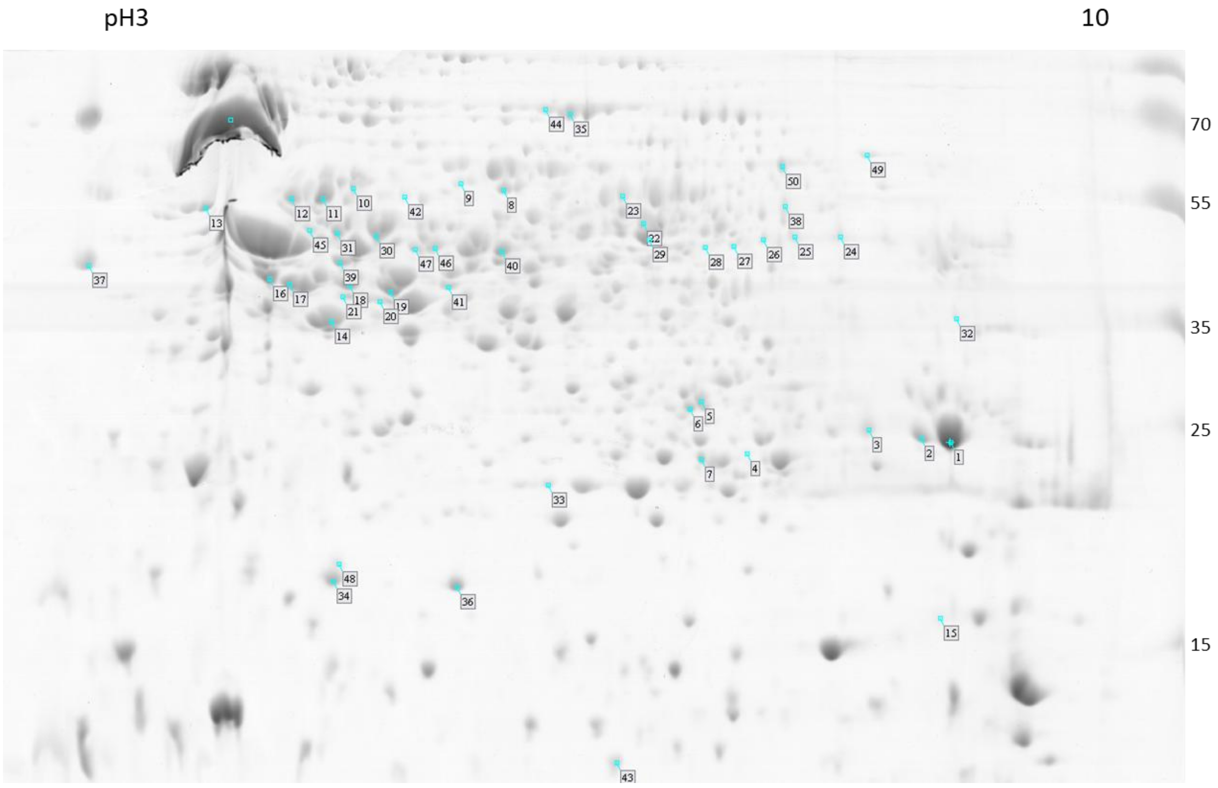

2C

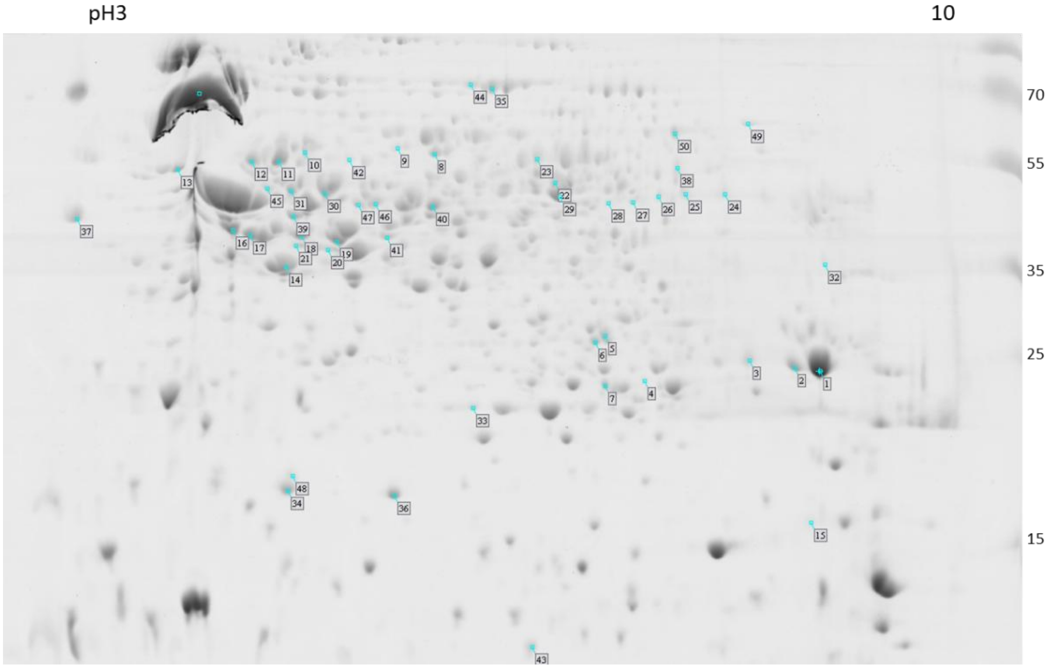

2D

Figure S2: Two-dimensional gel electrophoresis of protein extracts of *C. burneii* isolate 26QC00015 **(A)** stained with colloidal Coomassie and **(B)** Western blotting with pool of Q fever-positive serum from sheep pool 1 and **(C and D)** spot matching using Delta2D 4.7 (Decodon) and spots with distinct normalized volumes (immunostained spots > 0.1 and Coomassie stained spots > 0.2) were selected for protein identification (numbered, circled spots). Results of protein identification is displayed in supplemental table 2.

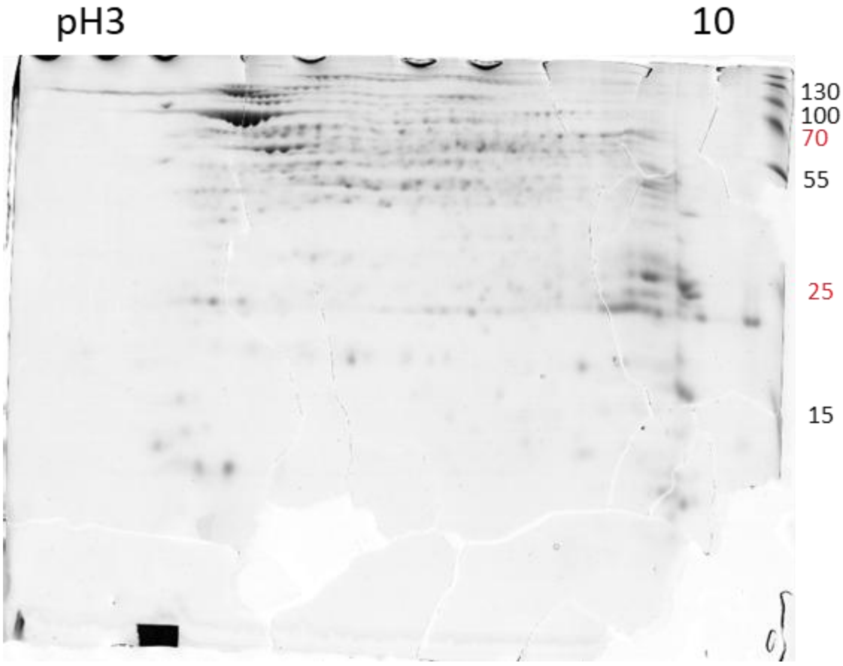

3A

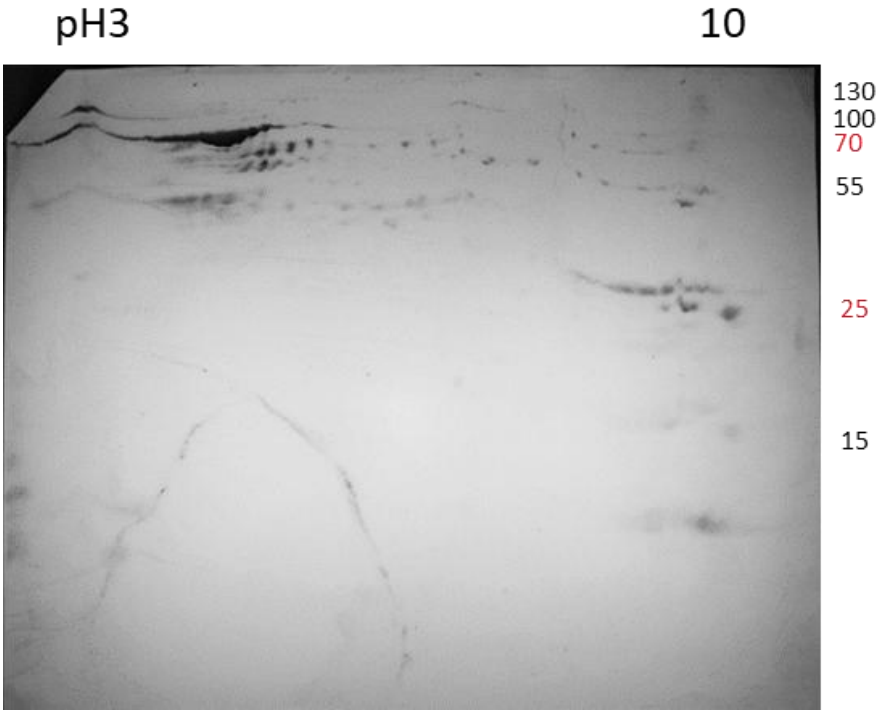

3B

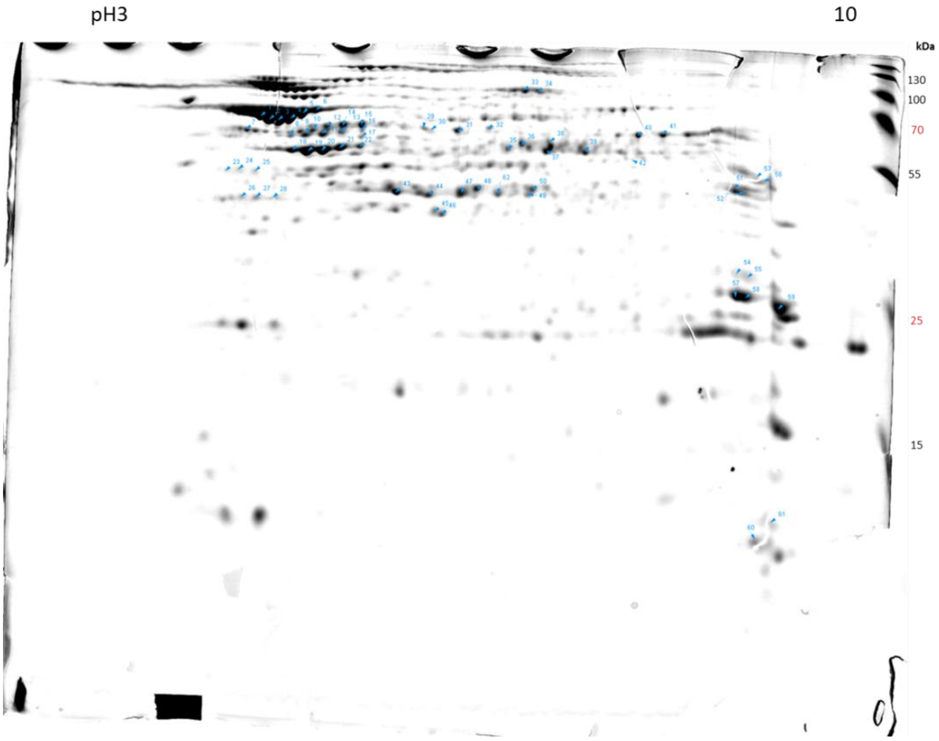

3C

Figure S3: Two-dimensional gel electrophoresis of protein extracts of *C. burneii* isolate 26QC00015 **(A)** stained with colloidal Coomassie and **(B)** Western blotting with pool of Q fever-positive serum from sheep pool 1 and **(C)** spot matching using Delta2D 4.7 (Decodon)

and spots with distinct normalized volumes (immunostained spots > 0.1 and Coomassie stained spots > 0.2) were selected for protein identification (numbered, circled spots). Results of protein identification is displayed in supplemental table 2.

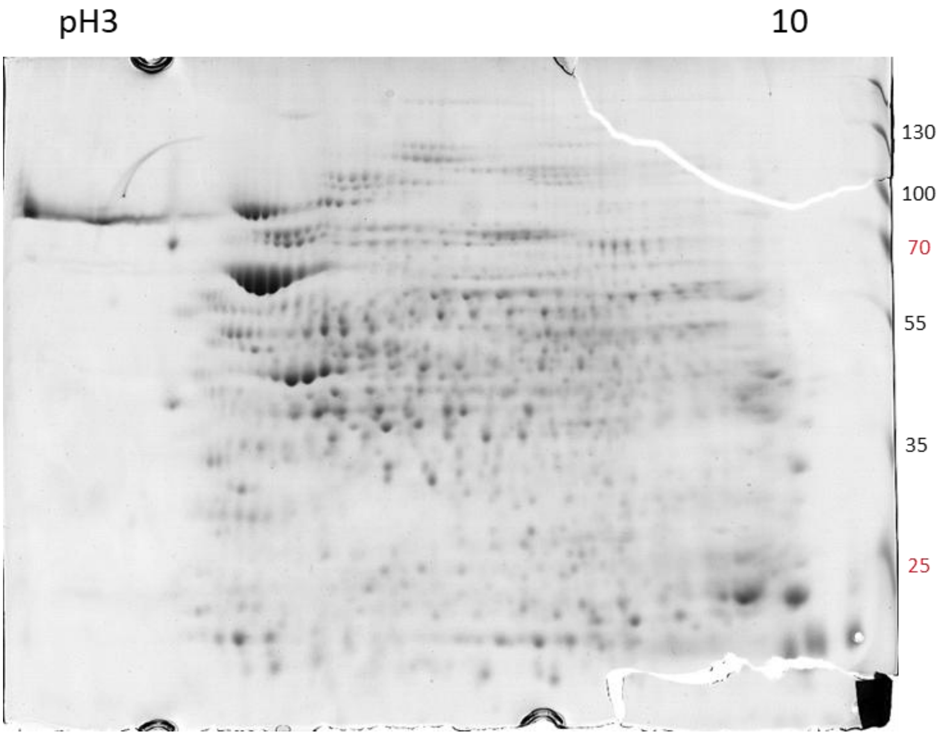

4A

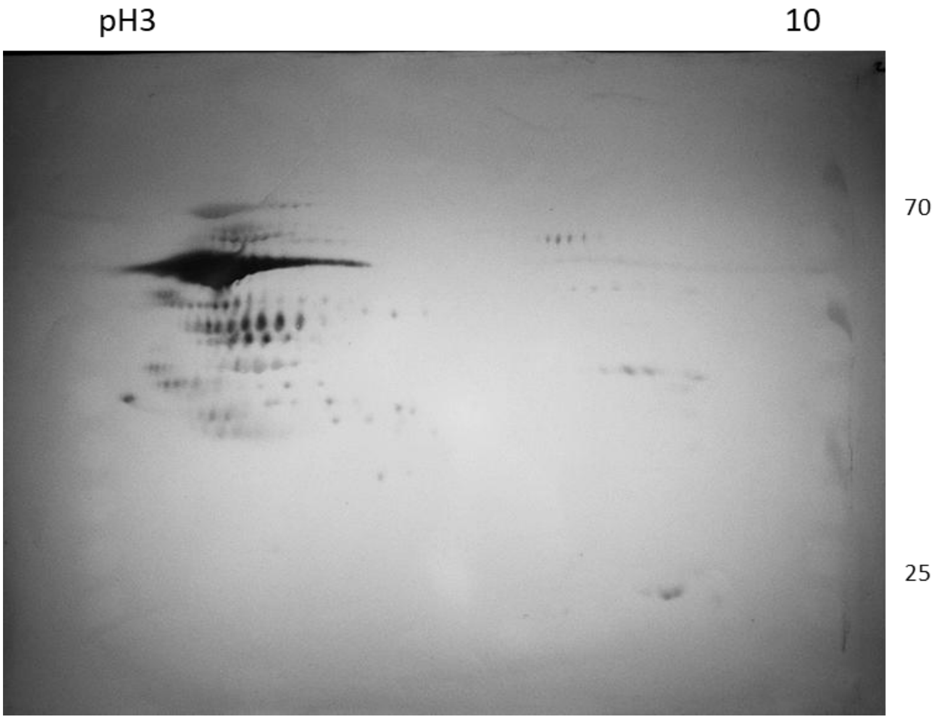

4B

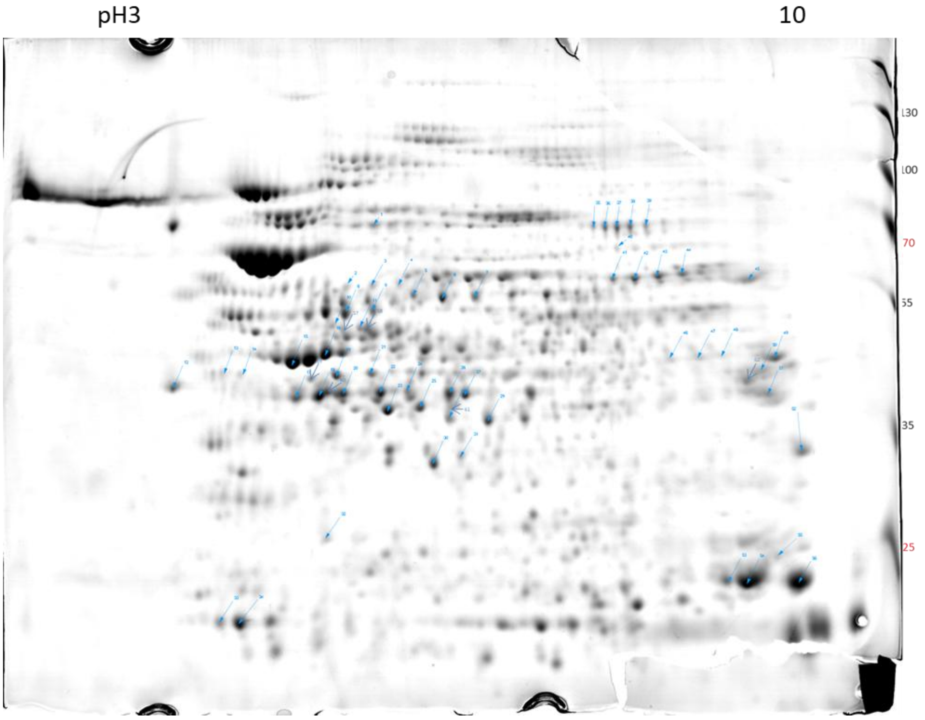

4C

Figure S4: Two-dimensional gel electrophoresis of protein extracts of *C. burneii* isolate 26QC00015 **(A)** stained with colloidal Coomassie and **(B)** Western blotting with pool of Q fever-positive serum from sheep pool 1 and **(C)** spot matching using Delta2D 4.7 (Decodon) and spots with distinct normalized volumes (immunostained spots > 0.1 and Coomassie stained spots > 0.2) were selected for protein identification (numbered, circled spots). Results of protein identification is displayed in supplemental table 2.

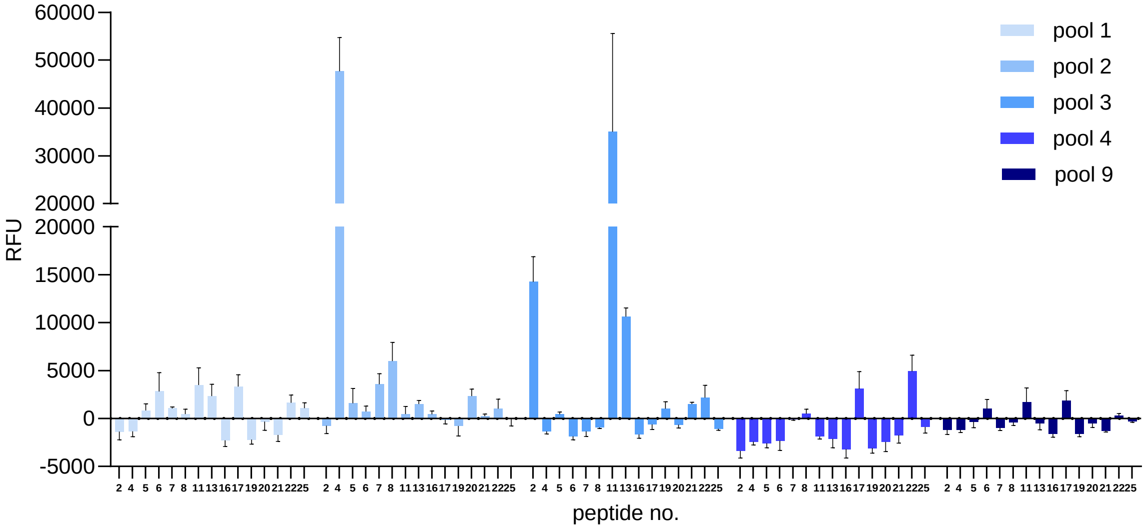

5A

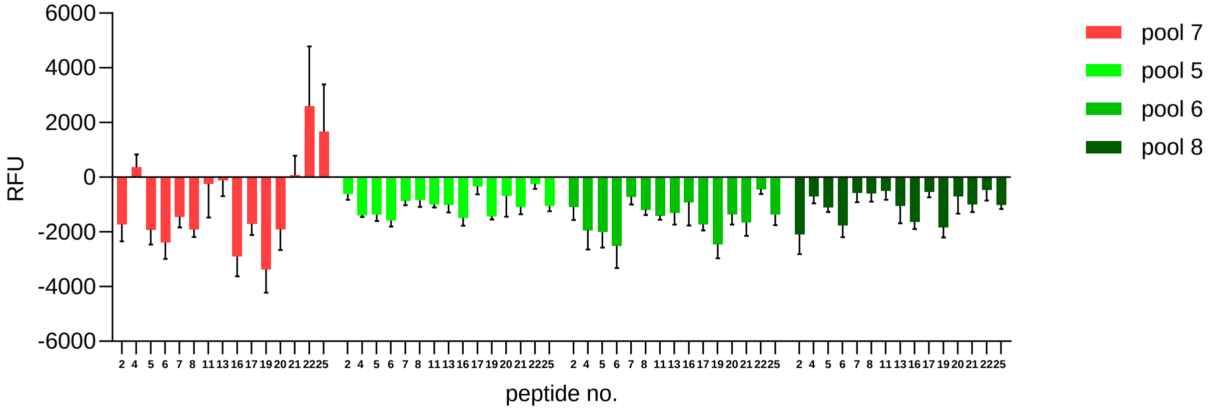

5B

Figure S5: Peptide reactivity with serum pools from Q fever-positive **(A)** or from vaccinated and Q fever-negative sheep flocks **(B)**. Reactivity of 15 peptides were measured using indirect ELISA method with serum pools from Q fever positive sheep flocks (pool 1 to 4; pool 9), from sheep vaccinated with COXEVAC (pool 7) or from Q fever-negative sheep flocks (pool 5; 6 and 8) 1:100 diluted. The reactivity was traced using a peroxidase conjugated anti-goat/sheep IgG monoclonal antibody (1:8000) and displayed as relative fluorescence units (RFU) after background reduction of 150% from uncoated wells. The data represent the results from three independent experiments. The diagrams were created using GraphPad Prism 10, Version 10.6.1.

ROC curve

Variable	Peptid_2
Classification variable	status

Sample size	75
Positive group [a]	58 (77,33%)
Negative group [b]	17 (22,67%)

[a] status = 1
[b] status = 0

Disease prevalence (%)	unknown

Area under the ROC curve (AUC)

Area under the ROC curve (AUC)	0.673
Standard Error [a]	0.0687
95% Confidence interval [b]	0,555 to 0,777
z statistic	2.518
Significance level P (Area=0.5)	0.0118

[a] DeLong et al., 1988
[b] Binomial exact

Youden index

Youden index J	0.3124
Associated criterion	≤-1068
Sensitivity	72.41
Specificity	58.82

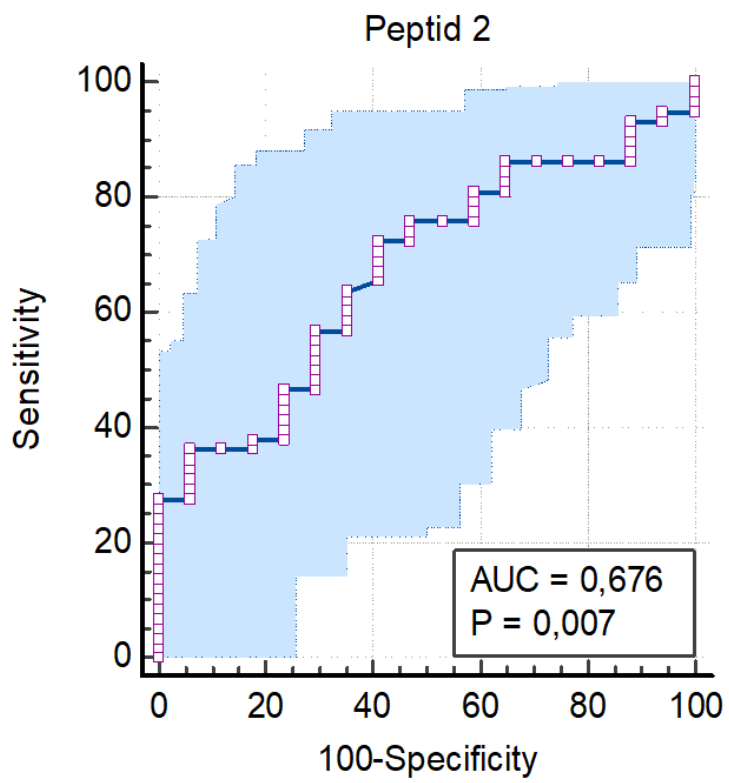

Criterion	Sensitivity	95% CI	Specificity	95% CI	+LR	95% CI	-LR	95% CI
<-6934	0	0,0 - 6,2	100	80,5 - 100,0			1	1,00 - 1,00
≤-6934	1.72	0,04 - 9,2	100	80,5 - 100,0			0.98	0,95 - 1,02
≤-4355	3.45	0,4 - 11,9	100	80,5 - 100,0			0.97	0,92 - 1,01
≤-3402	5.17	1,1 - 14,4	100	80,5 - 100,0			0.95	0,89 - 1,01
≤-3285	6.9	1,9 - 16,7	100	80,5 - 100,0			0.93	0,87 - 1,00
≤-3206	8.62	2,9 - 19,0	100	80,5 - 100,0			0.91	0,84 - 0,99
≤-2929	10.34	3,9 - 21,2	100	80,5 - 100,0			0.9	0,82 - 0,98
≤-2920	12.07	5,0 - 23,3	100	80,5 - 100,0			0.88	0,80 - 0,97
≤-2884	13.79	6,1 - 25,4	100	80,5 - 100,0			0.86	0,78 - 0,96
≤-2598	15.52	7,3 - 27,4	100	80,5 - 100,0			0.84	0,76 - 0,94
≤-2536	17.24	8,6 - 29,4	100	80,5 - 100,0			0.83	0,74 - 0,93
≤-2259	18.97	9,9 - 31,4	100	80,5 - 100,0			0.81	0,72 - 0,92
≤-2253	20.69	11,2 - 33,4	100	80,5 - 100,0			0.79	0,70 - 0,90
≤-2226	22.41	12,5 - 35,3	100	80,5 - 100,0			0.78	0,68 - 0,89
≤-2214	24.14	13,9 - 37,2	100	80,5 - 100,0			0.76	0,66 - 0,88
≤-2204	25.86	15,3 - 39,0	100	80,5 - 100,0			0.74	0,64 - 0,86
≤-2143	27.59	16,7 - 40,9	100	80,5 - 100,0			0.72	0,62 - 0,85
≤-2083	27.59	16,7 - 40,9	94.12	71,3 - 99,9	4.69	0,67 - 32,85	0.77	0,63 - 0,94
≤-1974	29.31	18,1 - 42,7	94.12	71,3 - 99,9	4.98	0,71 - 34,78	0.75	0,61 - 0,92
≤-1973	31.03	19,5 - 44,5	94.12	71,3 - 99,9	5.28	0,76 - 36,71	0.73	0,59 - 0,90
≤-1958	32.76	21,0 - 46,3	94.12	71,3 - 99,9	5.57	0,80 - 38,63	0.71	0,58 - 0,89
≤-1946	34.48	22,5 - 48,1	94.12	71,3 - 99,9	5.86	0,85 - 40,56	0.7	0,56 - 0,87
≤-1942	36.21	24,0 - 49,9	94.12	71,3 - 99,9	6.16	0,89 - 42,49	0.68	0,54 - 0,85
≤-1901	36.21	24,0 - 49,9	88.24	63,6 - 98,5	3.08	0,80 - 11,82	0.72	0,56 - 0,94

≤-1834	36.21	24,0 - 49,9	82.35	56,6 - 96,2	2.05	0,70 - 6,06	0.77	0,58 - 1,04
≤-1776	37.93	25,5 - 51,6	82.35	56,6 - 96,2	2.15	0,73 - 6,32	0.75	0,56 - 1,02
≤-1738	37.93	25,5 - 51,6	76.47	50,1 - 93,2	1.61	0,64 - 4,04	0.81	0,58 - 1,13
≤-1733	39.66	27,0 - 53,4	76.47	50,1 - 93,2	1.69	0,68 - 4,20	0.79	0,56 - 1,10
≤-1704	41.38	28,6 - 55,1	76.47	50,1 - 93,2	1.76	0,71 - 4,37	0.77	0,55 - 1,08
≤-1699	43.1	30,2 - 56,8	76.47	50,1 - 93,2	1.83	0,74 - 4,54	0.74	0,53 - 1,05
≤-1662	44.83	31,7 - 58,5	76.47	50,1 - 93,2	1.91	0,77 - 4,70	0.72	0,51 - 1,03
≤-1560	46.55	33,3 - 60,1	76.47	50,1 - 93,2	1.98	0,80 - 4,87	0.7	0,49 - 1,00
≤-1530	46.55	33,3 - 60,1	70.59	44,0 - 89,7	1.58	0,72 - 3,47	0.76	0,51 - 1,12
≤-1523	48.28	35,0 - 61,8	70.59	44,0 - 89,7	1.64	0,75 - 3,59	0.73	0,49 - 1,09
≤-1515	50	36,6 - 63,4	70.59	44,0 - 89,7	1.7	0,78 - 3,71	0.71	0,47 - 1,06
≤-1491	51.72	38,2 - 65,0	70.59	44,0 - 89,7	1.76	0,81 - 3,83	0.68	0,46 - 1,03
≤-1483	53.45	39,9 - 66,7	70.59	44,0 - 89,7	1.82	0,84 - 3,94	0.66	0,44 - 1,00
≤-1397	55.17	41,5 - 68,3	70.59	44,0 - 89,7	1.88	0,87 - 4,06	0.64	0,42 - 0,97
≤-1370	56.9	43,2 - 69,8	70.59	44,0 - 89,7	1.93	0,90 - 4,18	0.61	0,40 - 0,94
≤-1343	56.9	43,2 - 69,8	64.71	38,3 - 85,8	1.61	0,82 - 3,19	0.67	0,42 - 1,05
≤-1286	58.62	44,9 - 71,4	64.71	38,3 - 85,8	1.66	0,84 - 3,28	0.64	0,40 - 1,02
≤-1285	60.34	46,6 - 73,0	64.71	38,3 - 85,8	1.71	0,87 - 3,36	0.61	0,38 - 0,98
≤-1279	62.07	48,4 - 74,5	64.71	38,3 - 85,8	1.76	0,90 - 3,45	0.59	0,36 - 0,95
≤-1243	63.79	50,1 - 76,0	64.71	38,3 - 85,8	1.81	0,92 - 3,54	0.56	0,34 - 0,91
≤-1208	65.52	51,9 - 77,5	58.82	32,9 - 81,6	1.59	0,87 - 2,89	0.59	0,34 - 1,00
≤-1194	67.24	53,7 - 79,0	58.82	32,9 - 81,6	1.63	0,90 - 2,96	0.56	0,32 - 0,96
≤-1120	68.97	55,5 - 80,5	58.82	32,9 - 81,6	1.67	0,92 - 3,03	0.53	0,30 - 0,92
≤-1073	70.69	57,3 - 81,9	58.82	32,9 - 81,6	1.72	0,95 - 3,10	0.5	0,28 - 0,88
≤-1068	72.41	59,1 - 83,3	58.82	32,9 - 81,6	1.76	0,97 - 3,17	0.47	0,26 - 0,83
≤-1041	72.41	59,1 - 83,3	52.94	27,8 - 77,0	1.54	0,91 - 2,61	0.52	0,28 - 0,96

≤-1018	74.14	61,0 - 84,7	52.94	27,8 - 77,0	1.58	0,93 - 2,67	0.49	0,26 - 0,91
≤-978	75.86	62,8 - 86,1	52.94	27,8 - 77,0	1.61	0,95 - 2,72	0.46	0,24 - 0,86
≤-971	75.86	62,8 - 86,1	47.06	23,0 - 72,2	1.43	0,89 - 2,30	0.51	0,26 - 1,01
≤-756	75.86	62,8 - 86,1	41.18	18,4 - 67,1	1.29	0,84 - 1,97	0.59	0,28 - 1,21
≤-578	77.59	64,7 - 87,5	41.18	18,4 - 67,1	1.32	0,87 - 2,01	0.54	0,26 - 1,14
≤-555	79.31	66,6 - 88,8	41.18	18,4 - 67,1	1.35	0,89 - 2,05	0.5	0,24 - 1,07
≤-507	81.03	68,6 - 90,1	41.18	18,4 - 67,1	1.38	0,91 - 2,09	0.46	0,21 - 1,00
≤-389	81.03	68,6 - 90,1	35.29	14,2 - 61,7	1.25	0,86 - 1,82	0.54	0,23 - 1,24
≤-355	82.76	70,6 - 91,4	35.29	14,2 - 61,7	1.28	0,88 - 1,85	0.49	0,21 - 1,15
≤-249	84.48	72,6 - 92,7	35.29	14,2 - 61,7	1.31	0,90 - 1,89	0.44	0,18 - 1,06
≤-170	86.21	74,6 - 93,9	35.29	14,2 - 61,7	1.33	0,92 - 1,92	0.39	0,16 - 0,97
≤-152	86.21	74,6 - 93,9	29.41	10,3 - 56,0	1.22	0,88 - 1,69	0.47	0,18 - 1,25
≤-134	86.21	74,6 - 93,9	23.53	6,8 - 49,9	1.13	0,85 - 1,50	0.59	0,20 - 1,71
≤129	86.21	74,6 - 93,9	17.65	3,8 - 43,4	1.05	0,82 - 1,33	0.78	0,23 - 2,63
≤445	86.21	74,6 - 93,9	11.76	1,5 - 36,4	0.98	0,80 - 1,20	1.17	0,27 - 5,01
≤769	87.93	76,7 - 95,0	11.76	1,5 - 36,4	1	0,82 - 1,21	1.03	0,23 - 4,49
≤1879	89.66	78,8 - 96,1	11.76	1,5 - 36,4	1.02	0,84 - 1,23	0.88	0,19 - 3,97
≤1933	91.38	81,0 - 97,1	11.76	1,5 - 36,4	1.04	0,86 - 1,25	0.73	0,16 - 3,45
≤2057	93.1	83,3 - 98,1	11.76	1,5 - 36,4	1.06	0,88 - 1,27	0.59	0,12 - 2,93
≤2150	93.1	83,3 - 98,1	5.88	0,1 - 28,7	0.99	0,86 - 1,14	1.17	0,14 - 9,80
≤3478	94.83	85,6 - 98,9	5.88	0,1 - 28,7	1.01	0,88 - 1,15	0.88	0,098 - 7,92
≤4092	94.83	85,6 - 98,9	0	0,0 - 19,5	0.95	0,89 - 1,01		
≤5776	96.55	88,1 - 99,6	0	0,0 - 19,5	0.97	0,92 - 1,01		
≤17743	98.28	90,8 - 100,0	0	0,0 - 19,5	0.98	0,95 - 1,02		
≤21896	100	93,8 - 100,0	0	0,0 - 19,5	1	1,00 - 1,00		

Monday, September 22, 2025 16:55 - MedCalc® version 23.3.7

6A

frontiers

ROC curve

Variable	Peptid_4
Classification variable	status

Sample size	75
Positive group [a]	58 (77,33%)
Negative group [b]	17 (22,67%)

status = 1
[b] status = 0

Disease prevalence (%)	unknown

Area under the ROC curve (AUC)

Area under the ROC curve (AUC)	0.526
Standard Error [a]	0.0716
95% Confidence interval [b]	0,408 to 0,643
z statistic	0.368
Significance level P (Area=0.5)	0.7127

[a] DeLong et al., 1988
[b] Binomial exact

Youden index

Youden index J	0.2546
Associated criterion	≤-1744
Sensitivity	43.1
Specificity	82.35

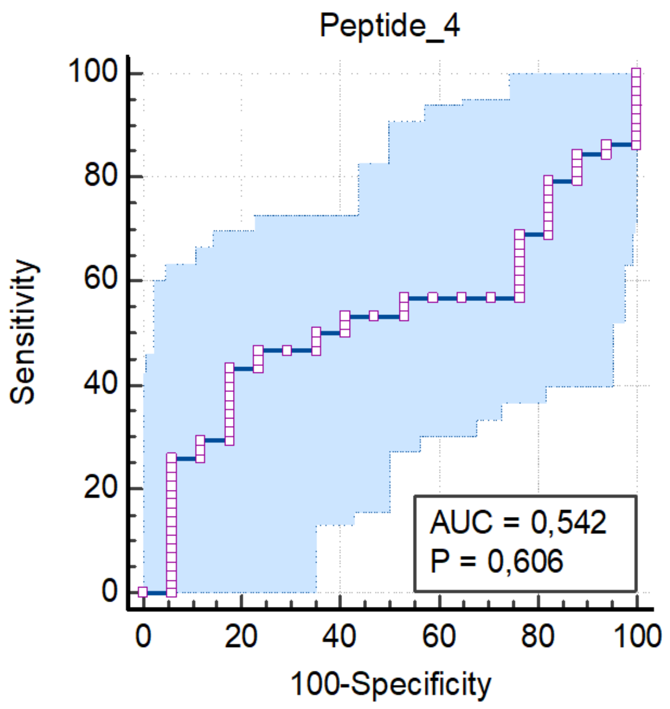

Criterion	Sensitivity	95% CI	Specificity	95% CI	+LR	95% CI	-LR	95% CI
<-9367	0	0,0 - 6,2	100	80,5 - 100,0			1	1,00 - 1,00
≤-9367	0	0,0 - 6,2	94.12	71,3 - 99,9	0		1.06	0,94 - 1,20
≤-6784	1.72	0,04 - 9,2	94.12	71,3 - 99,9	0.29	0,019 - 4,44	1.04	0,92 - 1,18
≤-6320	3.45	0,4 - 11,9	94.12	71,3 - 99,9	0.59	0,057 - 6,08	1.03	0,90 - 1,17
≤-4551	5.17	1,1 - 14,4	94.12	71,3 - 99,9	0.88	0,098 - 7,92	1.01	0,88 - 1,15
≤-4534	6.9	1,9 - 16,7	94.12	71,3 - 99,9	1.17	0,14 - 9,80	0.99	0,86 - 1,14
≤-4165	8.62	2,9 - 19,0	94.12	71,3 - 99,9	1.47	0,18 - 11,71	0.97	0,84 - 1,12
≤-3847	10.34	3,9 - 21,2	94.12	71,3 - 99,9	1.76	0,23 - 13,62	0.95	0,82 - 1,10
≤-3821	12.07	5,0 - 23,3	94.12	71,3 - 99,9	2.05	0,27 - 15,53	0.93	0,80 - 1,09
≤-3761	13.79	6,1 - 25,4	94.12	71,3 - 99,9	2.34	0,32 - 17,45	0.92	0,78 - 1,07
≤-3348	15.52	7,3 - 27,4	94.12	71,3 - 99,9	2.64	0,36 - 19,38	0.9	0,76 - 1,06
≤-3346	17.24	8,6 - 29,4	94.12	71,3 - 99,9	2.93	0,40 - 21,30	0.88	0,74 - 1,04
≤-2867	18.97	9,9 - 31,4	94.12	71,3 - 99,9	3.22	0,45 - 23,22	0.86	0,72 - 1,02
≤-2310	20.69	11,2 - 33,4	94.12	71,3 - 99,9	3.52	0,49 - 25,15	0.84	0,71 - 1,01
≤-2185	22.41	12,5 - 35,3	94.12	71,3 - 99,9	3.81	0,54 - 27,07	0.82	0,69 - 0,99
≤-2184	24.14	13,9 - 37,2	94.12	71,3 - 99,9	4.1	0,58 - 29,00	0.81	0,67 - 0,97
≤-2163	25.86	15,3 - 39,0	94.12	71,3 - 99,9	4.4	0,63 - 30,93	0.79	0,65 - 0,96
≤-2096	25.86	15,3 - 39,0	88.24	63,6 - 98,5	2.2	0,56 - 8,68	0.84	0,67 - 1,06
≤-1983	27.59	16,7 - 40,9	88.24	63,6 - 98,5	2.34	0,60 - 9,20	0.82	0,65 - 1,04
≤-1949	29.31	18,1 - 42,7	88.24	63,6 - 98,5	2.49	0,64 - 9,72	0.8	0,63 - 1,02
≤-1927	29.31	18,1 - 42,7	82.35	56,6 - 96,2	1.66	0,55 - 5,00	0.86	0,65 - 1,13
≤-1926	31.03	19,5 - 44,5	82.35	56,6 - 96,2	1.76	0,59 - 5,26	0.84	0,63 - 1,11
≤-1925	32.76	21,0 - 46,3	82.35	56,6 - 96,2	1.86	0,62 - 5,53	0.82	0,61 - 1,08
≤-1920	34.48	22,5 - 48,1	82.35	56,6 - 96,2	1.95	0,66 - 5,79	0.8	0,60 - 1,06
≤-1902	36.21	24,0 - 49,9	82.35	56,6 - 96,2	2.05	0,70 - 6,06	0.77	0,58 - 1,04

≤-1858	37.93	25,5 - 51,6	82.35	56,6 - 96,2	2.15	0,73 - 6,32	0.75	0,56 - 1,02
≤-1796	39.66	27,0 - 53,4	82.35	56,6 - 96,2	2.25	0,77 - 6,58	0.73	0,54 - 0,99
≤-1774	41.38	28,6 - 55,1	82.35	56,6 - 96,2	2.34	0,80 - 6,85	0.71	0,52 - 0,97
≤-1744	43.1	30,2 - 56,8	82.35	56,6 - 96,2	2.44	0,84 - 7,11	0.69	0,50 - 0,95
≤-1713	43.1	30,2 - 56,8	76.47	50,1 - 93,2	1.83	0,74 - 4,54	0.74	0,53 - 1,05
≤-1707	44.83	31,7 - 58,5	76.47	50,1 - 93,2	1.91	0,77 - 4,70	0.72	0,51 - 1,03
≤-1692	46.55	33,3 - 60,1	76.47	50,1 - 93,2	1.98	0,80 - 4,87	0.7	0,49 - 1,00
≤-1637	46.55	33,3 - 60,1	70.59	44,0 - 89,7	1.58	0,72 - 3,47	0.76	0,51 - 1,12
≤-1623	46.55	33,3 - 60,1	64.71	38,3 - 85,8	1.32	0,65 - 2,66	0.83	0,54 - 1,26
≤-1595	48.28	35,0 - 61,8	64.71	38,3 - 85,8	1.37	0,68 - 2,75	0.8	0,52 - 1,23
≤-1554	50	36,6 - 63,4	64.71	38,3 - 85,8	1.42	0,71 - 2,83	0.77	0,50 - 1,19
≤-1535	50	36,6 - 63,4	58.82	32,9 - 81,6	1.21	0,65 - 2,27	0.85	0,53 - 1,37
≤-1524	51.72	38,2 - 65,0	58.82	32,9 - 81,6	1.26	0,68 - 2,34	0.82	0,51 - 1,32
≤-1472	53.45	39,9 - 66,7	58.82	32,9 - 81,6	1.3	0,70 - 2,41	0.79	0,49 - 1,28
≤-1430	53.45	39,9 - 66,7	52.94	27,8 - 77,0	1.14	0,65 - 1,99	0.88	0,52 - 1,49
≤-1420	53.45	39,9 - 66,7	47.06	23,0 - 72,2	1.01	0,61 - 1,68	0.99	0,56 - 1,76
≤-1367	55.17	41,5 - 68,3	47.06	23,0 - 72,2	1.04	0,63 - 1,73	0.95	0,53 - 1,70
≤-1334	56.9	43,2 - 69,8	47.06	23,0 - 72,2	1.07	0,65 - 1,77	0.92	0,51 - 1,64
≤-1321	56.9	43,2 - 69,8	41.18	18,4 - 67,1	0.97	0,61 - 1,53	1.05	0,55 - 1,99
≤-1250	56.9	43,2 - 69,8	35.29	14,2 - 61,7	0.88	0,58 - 1,33	1.22	0,60 - 2,48
≤-1223	56.9	43,2 - 69,8	29.41	10,3 - 56,0	0.81	0,55 - 1,18	1.47	0,66 - 3,24
≤-1189	56.9	43,2 - 69,8	23.53	6,8 - 49,9	0.74	0,53 - 1,05	1.83	0,74 - 4,54
≤-1179	58.62	44,9 - 71,4	23.53	6,8 - 49,9	0.77	0,55 - 1,08	1.76	0,71 - 4,37
≤-1127	60.34	46,6 - 73,0	23.53	6,8 - 49,9	0.79	0,56 - 1,10	1.69	0,68 - 4,20
≤-1077	62.07	48,4 - 74,5	23.53	6,8 - 49,9	0.81	0,58 - 1,13	1.61	0,64 - 4,04
≤-1057	63.79	50,1 - 76,0	23.53	6,8 - 49,9	0.83	0,60 - 1,16	1.54	0,61 - 3,87

≤-1038	65.52	51,9 - 77,5	23.53	6,8 - 49,9	0.86	0,62 - 1,18	1.47	0,58 - 3,71
≤-1012	67.24	53,7 - 79,0	23.53	6,8 - 49,9	0.88	0,64 - 1,21	1.39	0,55 - 3,54
≤-949	68.97	55,5 - 80,5	23.53	6,8 - 49,9	0.9	0,66 - 1,24	1.32	0,52 - 3,37
≤-936	68.97	55,5 - 80,5	17.65	3,8 - 43,4	0.84	0,63 - 1,11	1.76	0,59 - 5,26
≤-865	70.69	57,3 - 81,9	17.65	3,8 - 43,4	0.86	0,65 - 1,13	1.66	0,55 - 5,00
≤-853	72.41	59,1 - 83,3	17.65	3,8 - 43,4	0.88	0,67 - 1,15	1.56	0,52 - 4,74
≤-833	74.14	61,0 - 84,7	17.65	3,8 - 43,4	0.9	0,69 - 1,18	1.47	0,48 - 4,47
≤-782	75.86	62,8 - 86,1	17.65	3,8 - 43,4	0.92	0,71 - 1,20	1.37	0,44 - 4,21
≤-731	77.59	64,7 - 87,5	17.65	3,8 - 43,4	0.94	0,73 - 1,22	1.27	0,41 - 3,94
≤-638	79.31	66,6 - 88,8	17.65	3,8 - 43,4	0.96	0,75 - 1,24	1.17	0,37 - 3,68
≤-572	79.31	66,6 - 88,8	11.76	1,5 - 36,4	0.9	0,72 - 1,12	1.76	0,44 - 7,10
≤-476	81.03	68,6 - 90,1	11.76	1,5 - 36,4	0.92	0,74 - 1,14	1.61	0,40 - 6,58
≤-398	82.76	70,6 - 91,4	11.76	1,5 - 36,4	0.94	0,76 - 1,16	1.47	0,35 - 6,06
≤-98	84.48	72,6 - 92,7	11.76	1,5 - 36,4	0.96	0,78 - 1,18	1.32	0,31 - 5,53
≤-97	84.48	72,6 - 92,7	5.88	0,1 - 28,7	0.9	0,76 - 1,06	2.64	0,36 - 19,38
≤309	86.21	74,6 - 93,9	5.88	0,1 - 28,7	0.92	0,78 - 1,07	2.34	0,32 - 17,45
≤878	86.21	74,6 - 93,9	0	0,0 - 19,5	0.86	0,78 - 0,96		
≤1556	87.93	76,7 - 95,0	0	0,0 - 19,5	0.88	0,80 - 0,97		
≤2034	89.66	78,8 - 96,1	0	0,0 - 19,5	0.9	0,82 - 0,98		
≤3650	91.38	81,0 - 97,1	0	0,0 - 19,5	0.91	0,84 - 0,99		
≤4659	93.1	83,3 - 98,1	0	0,0 - 19,5	0.93	0,87 - 1,00		
≤19881	94.83	85,6 - 98,9	0	0,0 - 19,5	0.95	0,89 - 1,01		
≤47439	96.55	88,1 - 99,6	0	0,0 - 19,5	0.97	0,92 - 1,01		
≤49118	98.28	90,8 - 100,0	0	0,0 - 19,5	0.98	0,95 - 1,02		
≤50133	100	93,8 - 100,0	0	0,0 - 19,5	1	1,00 - 1,00		

Monday, September 22, 2025 16:54 - MedCalc® version 23.3.7

6B

ROC curve

Variable	Peptid_5
Classification variable	status

Sample size	75
Positive group [a]	58 (77,33%)
Negative group [b]	17 (22,67%)

[a] status = 1
[b] status = 0

Disease prevalence (%)	unknown

Area under the ROC curve (AUC)

Area under the ROC curve (AUC)	0.559
Standard Error [a]	0.0743
95% Confidence interval [b]	0,439 to 0,673
z statistic	0.792
Significance level P (Area=0.5)	0.4283

[a] DeLong et al., 1988
[b] Binomial exact

Youden index

Youden index J	0.2343
Associated criterion	>77
Sensitivity	29.31
Specificity	94.12

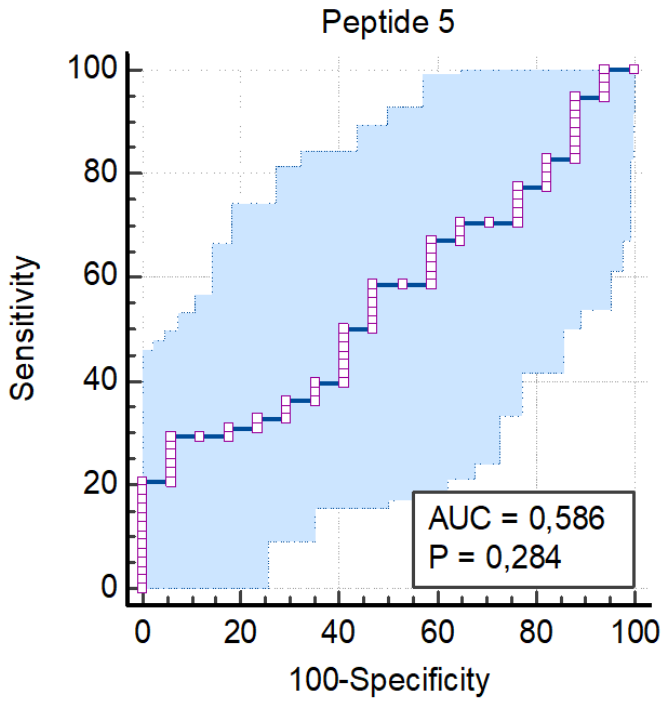

Criterion	Sensitivity	95% CI	Specificity	95% CI	+LR	95% CI	-LR	95% CI
≥-10661	100	93,8 - 100,0	0	0,0 - 19,5	1	1,00 - 1,00		
>-10661	100	93,8 - 100,0	5.88	0,1 - 28,7	1.06	0,94 - 1,20	0	
>-6473	98.28	90,8 - 100,0	5.88	0,1 - 28,7	1.04	0,92 - 1,18	0.29	0,019 - 4,44
>-3109	96.55	88,1 - 99,6	5.88	0,1 - 28,7	1.03	0,90 - 1,17	0.59	0,057 - 6,08
>-2811	94.83	85,6 - 98,9	5.88	0,1 - 28,7	1.01	0,88 - 1,15	0.88	0,098 - 7,92
>-2720	94.83	85,6 - 98,9	11.76	1,5 - 36,4	1.07	0,89 - 1,29	0.44	0,080 - 2,42
>-2281	93.1	83,3 - 98,1	11.76	1,5 - 36,4	1.06	0,88 - 1,27	0.59	0,12 - 2,93
>-2279	91.38	81,0 - 97,1	11.76	1,5 - 36,4	1.04	0,86 - 1,25	0.73	0,16 - 3,45
>-2188	89.66	78,8 - 96,1	11.76	1,5 - 36,4	1.02	0,84 - 1,23	0.88	0,19 - 3,97
>-2082	87.93	76,7 - 95,0	11.76	1,5 - 36,4	1	0,82 - 1,21	1.03	0,23 - 4,49
>-2049	86.21	74,6 - 93,9	11.76	1,5 - 36,4	0.98	0,80 - 1,20	1.17	0,27 - 5,01
>-1882	84.48	72,6 - 92,7	11.76	1,5 - 36,4	0.96	0,78 - 1,18	1.32	0,31 - 5,53
>-1657	82.76	70,6 - 91,4	11.76	1,5 - 36,4	0.94	0,76 - 1,16	1.47	0,35 - 6,06
>-1602	82.76	70,6 - 91,4	17.65	3,8 - 43,4	1	0,78 - 1,29	0.98	0,30 - 3,15
>-1489	81.03	68,6 - 90,1	17.65	3,8 - 43,4	0.98	0,76 - 1,27	1.07	0,34 - 3,42
>-1480	79.31	66,6 - 88,8	17.65	3,8 - 43,4	0.96	0,75 - 1,24	1.17	0,37 - 3,68
>-1479	77.59	64,7 - 87,5	17.65	3,8 - 43,4	0.94	0,73 - 1,22	1.27	0,41 - 3,94
>-1471	77.59	64,7 - 87,5	23.53	6,8 - 49,9	1.01	0,75 - 1,37	0.95	0,36 - 2,54
>-1448	75.86	62,8 - 86,1	23.53	6,8 - 49,9	0.99	0,73 - 1,34	1.03	0,39 - 2,71
>-1389	74.14	61,0 - 84,7	23.53	6,8 - 49,9	0.97	0,72 - 1,31	1.1	0,42 - 2,87
>-1379	72.41	59,1 - 83,3	23.53	6,8 - 49,9	0.95	0,70 - 1,29	1.17	0,45 - 3,04
>-1354	70.69	57,3 - 81,9	23.53	6,8 - 49,9	0.92	0,68 - 1,26	1.25	0,48 - 3,21
>-1318	70.69	57,3 - 81,9	29.41	10,3 - 56,0	1	0,71 - 1,42	1	0,43 - 2,30
>-1304	70.69	57,3 - 81,9	35.29	14,2 - 61,7	1.09	0,74 - 1,61	0.83	0,39 - 1,77

>-1263	68.97	55,5 - 80,5	35.29	14,2 - 61,7	1.07	0,72 - 1,58	0.88	0,42 - 1,86
>-1262	67.24	53,7 - 79,0	35.29	14,2 - 61,7	1.04	0,70 - 1,54	0.93	0,44 - 1,95
>-1247	67.24	53,7 - 79,0	41.18	18,4 - 67,1	1.14	0,74 - 1,77	0.8	0,40 - 1,57
>-1246	65.52	51,9 - 77,5	41.18	18,4 - 67,1	1.11	0,72 - 1,73	0.84	0,43 - 1,64
>-1229	63.79	50,1 - 76,0	41.18	18,4 - 67,1	1.08	0,70 - 1,69	0.88	0,45 - 1,71
>-1214	62.07	48,4 - 74,5	41.18	18,4 - 67,1	1.06	0,68 - 1,65	0.92	0,48 - 1,78
>-1209	60.34	46,6 - 73,0	41.18	18,4 - 67,1	1.03	0,65 - 1,61	0.96	0,50 - 1,85
>-1181	58.62	44,9 - 71,4	41.18	18,4 - 67,1	1	0,63 - 1,57	1	0,53 - 1,92
>-1120	58.62	44,9 - 71,4	47.06	23,0 - 72,2	1.11	0,67 - 1,82	0.88	0,49 - 1,59
>-1034	58.62	44,9 - 71,4	52.94	27,8 - 77,0	1.25	0,72 - 2,16	0.78	0,45 - 1,35
>-1017	56.9	43,2 - 69,8	52.94	27,8 - 77,0	1.21	0,70 - 2,10	0.81	0,48 - 1,39
>-945	55.17	41,5 - 68,3	52.94	27,8 - 77,0	1.17	0,67 - 2,04	0.85	0,50 - 1,44
>-943	53.45	39,9 - 66,7	52.94	27,8 - 77,0	1.14	0,65 - 1,99	0.88	0,52 - 1,49
>-880	51.72	38,2 - 65,0	52.94	27,8 - 77,0	1.1	0,63 - 1,93	0.91	0,54 - 1,54
>-871	50	36,6 - 63,4	52.94	27,8 - 77,0	1.06	0,60 - 1,87	0.94	0,56 - 1,58
>-842	50	36,6 - 63,4	58.82	32,9 - 81,6	1.21	0,65 - 2,27	0.85	0,53 - 1,37
>-813	48.28	35,0 - 61,8	58.82	32,9 - 81,6	1.17	0,63 - 2,20	0.88	0,55 - 1,41
>-777	46.55	33,3 - 60,1	58.82	32,9 - 81,6	1.13	0,60 - 2,13	0.91	0,57 - 1,45
>-755	44.83	31,7 - 58,5	58.82	32,9 - 81,6	1.09	0,58 - 2,06	0.94	0,59 - 1,49
>-695	43.1	30,2 - 56,8	58.82	32,9 - 81,6	1.05	0,55 - 1,99	0.97	0,61 - 1,53
>-667	41.38	28,6 - 55,1	58.82	32,9 - 81,6	1	0,53 - 1,92	1	0,63 - 1,57
>-592	39.66	27,0 - 53,4	58.82	32,9 - 81,6	0.96	0,50 - 1,85	1.03	0,65 - 1,61
>-558	39.66	27,0 - 53,4	64.71	38,3 - 85,8	1.12	0,55 - 2,30	0.93	0,62 - 1,40
>-544	37.93	25,5 - 51,6	64.71	38,3 - 85,8	1.07	0,52 - 2,21	0.96	0,64 - 1,44
>-412	36.21	24,0 - 49,9	64.71	38,3 - 85,8	1.03	0,50 - 2,13	0.99	0,66 - 1,47
>-357	36.21	24,0 - 49,9	70.59	44,0 - 89,7	1.23	0,55 - 2,77	0.9	0,63 - 1,30

>-216	34.48	22,5 - 48,1	70.59	44,0 - 89,7	1.17	0,52 - 2,66	0.93	0,65 - 1,33
>-205	32.76	21,0 - 46,3	70.59	44,0 - 89,7	1.11	0,49 - 2,54	0.95	0,67 - 1,36
>-161	32.76	21,0 - 46,3	76.47	50,1 - 93,2	1.39	0,55 - 3,54	0.88	0,64 - 1,21
>-109	31.03	19,5 - 44,5	76.47	50,1 - 93,2	1.32	0,52 - 3,37	0.9	0,66 - 1,24
>-40	31.03	19,5 - 44,5	82.35	56,6 - 96,2	1.76	0,59 - 5,26	0.84	0,63 - 1,11
>39	29.31	18,1 - 42,7	82.35	56,6 - 96,2	1.66	0,55 - 5,00	0.86	0,65 - 1,13
>63	29.31	18,1 - 42,7	88.24	63,6 - 98,5	2.49	0,64 - 9,72	0.8	0,63 - 1,02
>77	29.31	18,1 - 42,7	94.12	71,3 - 99,9	4.98	0,71 - 34,78	0.75	0,61 - 0,92
>114	27.59	16,7 - 40,9	94.12	71,3 - 99,9	4.69	0,67 - 32,85	0.77	0,63 - 0,94
>360	25.86	15,3 - 39,0	94.12	71,3 - 99,9	4.4	0,63 - 30,93	0.79	0,65 - 0,96
>629	24.14	13,9 - 37,2	94.12	71,3 - 99,9	4.1	0,58 - 29,00	0.81	0,67 - 0,97
>899	22.41	12,5 - 35,3	94.12	71,3 - 99,9	3.81	0,54 - 27,07	0.82	0,69 - 0,99
>1088	20.69	11,2 - 33,4	94.12	71,3 - 99,9	3.52	0,49 - 25,15	0.84	0,71 - 1,01
>1175	20.69	11,2 - 33,4	100	80,5 - 100,0			0.79	0,70 - 0,90
>1534	18.97	9,9 - 31,4	100	80,5 - 100,0			0.81	0,72 - 0,92
>1976	17.24	8,6 - 29,4	100	80,5 - 100,0			0.83	0,74 - 0,93
>2452	15.52	7,3 - 27,4	100	80,5 - 100,0			0.84	0,76 - 0,94
>3585	13.79	6,1 - 25,4	100	80,5 - 100,0			0.86	0,78 - 0,96
>7011	12.07	5,0 - 23,3	100	80,5 - 100,0			0.88	0,80 - 0,97
>10057	10.34	3,9 - 21,2	100	80,5 - 100,0			0.9	0,82 - 0,98
>10106	8.62	2,9 - 19,0	100	80,5 - 100,0			0.91	0,84 - 0,99
>13685	6.9	1,9 - 16,7	100	80,5 - 100,0			0.93	0,87 - 1,00
>13954	5.17	1,1 - 14,4	100	80,5 - 100,0			0.95	0,89 - 1,01
>18296	3.45	0,4 - 11,9	100	80,5 - 100,0			0.97	0,92 - 1,01
>22899	1.72	0,04 - 9,2	100	80,5 - 100,0			0.98	0,95 - 1,02
>38709	0	0,0 - 6,2	100	80,5 - 100,0			1	1,00 - 1,00

Monday, September 22, 2025 17:03 - MedCalc® version 23.3.7

6C

ROC curve

Variable	Peptide_6
Classification variable	status

Sample size	75
Positive group [a]	58 (77,33%)
Negative group [b]	17 (22,67%)

[a] status = 1

[b] status = 0

Disease prevalence (%)	unknown

Area under the ROC curve (AUC)

Area under the ROC curve (AUC)	0.685
Standard Error [a]	0.0716
95% Confidence interval [b]	0,567 to 0,787
95% Bootstrap CI [c]	0,514 to 0,807
z statistic	2.579
Significance level P (Area=0.5)	0.0099

[a] DeLong et al., 1988

[b] Binomial exact

[c] BC_a bootstrap confidence interval (1000 iterations; random number seed: 928).

Youden index

Youden index J	0.4341
95% Confidence interval [a]	0,2031 to 0,5862
Associated criterion	>-1025
95% Confidence interval [a]	>-1031 to >7761
Sensitivity	55.17
Specificity	88.24

Summary Table

Estimated specificity at fixed sensitivity			
Sensitivity	Specificity	95% CI [a]	Criterion
80	29.41	0,00 to 52,94	>-2565,4
90	23.53	0,00 to 47,06	>-4242,4
95	17.65	0,00 to 41,18	>-5353,5
97.5	5.88	0,00 to 17,65	>-9734,15
99	0	5,88 to 5,88	>-19598
**Estimated sensitivity at fixed specificity**			
Specificity	Sensitivity	95% CI [a]	Criterion
80	55.17	19,79 to 69,60	>-1039,8
90	32.76	0,00 to 60,34	>-141,7
95	5.17	0,00 to 34,48	>7913,25
97.5	5.17	0,00 to 0,00	>8344,625
99	5.17	0,00 to 0,00	>8603,45

[a] BC_a bootstrap confidence interval (1000 iterations; random number seed: 928).

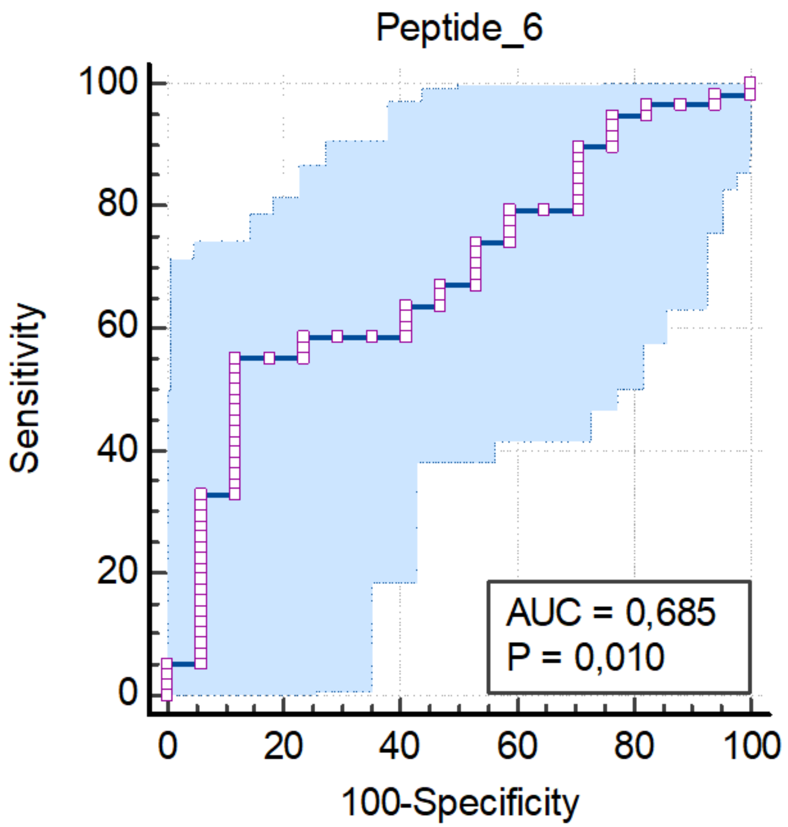

Criterion	Sensitivity	95% CI	Specificity	95% CI	+LR	95% CI	-LR	95% CI
≥-19598	100	93,8 - 100,0	0	0,0 - 19,5	1	1,00 - 1,00		
>-19598	98.28	90,8 - 100,0	0	0,0 - 19,5	0.98	0,95 - 1,02		
>-10028	98.28	90,8 - 100,0	5.88	0,1 - 28,7	1.04	0,92 - 1,18	0.29	0,019 - 4,44
>-9375	96.55	88,1 - 99,6	5.88	0,1 - 28,7	1.03	0,90 - 1,17	0.59	0,057 - 6,08
>-7182	96.55	88,1 - 99,6	11.76	1,5 - 36,4	1.09	0,91 - 1,31	0.29	0,045 - 1,93
>-5556	96.55	88,1 - 99,6	17.65	3,8 - 43,4	1.17	0,94 - 1,47	0.2	0,035 - 1,08
>-5331	94.83	85,6 - 98,9	17.65	3,8 - 43,4	1.15	0,92 - 1,45	0.29	0,065 - 1,32
>-5179	94.83	85,6 - 98,9	23.53	6,8 - 49,9	1.24	0,95 - 1,63	0.22	0,054 - 0,89
>-4477	93.1	83,3 - 98,1	23.53	6,8 - 49,9	1.22	0,93 - 1,60	0.29	0,082 - 1,05
>-4264	91.38	81,0 - 97,1	23.53	6,8 - 49,9	1.19	0,91 - 1,57	0.37	0,11 - 1,21
>-4237	89.66	78,8 - 96,1	23.53	6,8 - 49,9	1.17	0,89 - 1,55	0.44	0,14 - 1,38
>-4032	89.66	78,8 - 96,1	29.41	10,3 - 56,0	1.27	0,92 - 1,75	0.35	0,12 - 1,01
>-3467	87.93	76,7 - 95,0	29.41	10,3 - 56,0	1.25	0,90 - 1,72	0.41	0,15 - 1,13
>-3113	86.21	74,6 - 93,9	29.41	10,3 - 56,0	1.22	0,88 - 1,69	0.47	0,18 - 1,25
>-3023	84.48	72,6 - 92,7	29.41	10,3 - 56,0	1.2	0,86 - 1,66	0.53	0,20 - 1,36
>-2844	82.76	70,6 - 91,4	29.41	10,3 - 56,0	1.17	0,84 - 1,63	0.59	0,23 - 1,48
>-2569	81.03	68,6 - 90,1	29.41	10,3 - 56,0	1.15	0,82 - 1,60	0.64	0,26 - 1,60
>-2563	79.31	66,6 - 88,8	29.41	10,3 - 56,0	1.12	0,80 - 1,57	0.7	0,29 - 1,72
>-2526	79.31	66,6 - 88,8	35.29	14,2 - 61,7	1.23	0,84 - 1,78	0.59	0,26 - 1,33
>-2368	79.31	66,6 - 88,8	41.18	18,4 - 67,1	1.35	0,89 - 2,05	0.5	0,24 - 1,07
>-2279	77.59	64,7 - 87,5	41.18	18,4 - 67,1	1.32	0,87 - 2,01	0.54	0,26 - 1,14
>-2174	75.86	62,8 - 86,1	41.18	18,4 - 67,1	1.29	0,84 - 1,97	0.59	0,28 - 1,21
>-2077	74.14	61,0 - 84,7	41.18	18,4 - 67,1	1.26	0,82 - 1,93	0.63	0,31 - 1,29

>-2021	74.14	61,0 - 84,7	47.06	23,0 - 72,2	1.4	0,87 - 2,25	0.55	0,28 - 1,07
>-2009	72.41	59,1 - 83,3	47.06	23,0 - 72,2	1.37	0,85 - 2,20	0.59	0,30 - 1,13
>-1800	70.69	57,3 - 81,9	47.06	23,0 - 72,2	1.34	0,83 - 2,15	0.62	0,33 - 1,19
>-1642	68.97	55,5 - 80,5	47.06	23,0 - 72,2	1.3	0,81 - 2,11	0.66	0,35 - 1,24
>-1633	67.24	53,7 - 79,0	47.06	23,0 - 72,2	1.27	0,78 - 2,06	0.7	0,37 - 1,30
>-1618	67.24	53,7 - 79,0	52.94	27,8 - 77,0	1.43	0,84 - 2,44	0.62	0,35 - 1,11
>-1510	65.52	51,9 - 77,5	52.94	27,8 - 77,0	1.39	0,81 - 2,38	0.65	0,37 - 1,15
>-1469	63.79	50,1 - 76,0	52.94	27,8 - 77,0	1.36	0,79 - 2,33	0.68	0,39 - 1,20
>-1383	63.79	50,1 - 76,0	58.82	32,9 - 81,6	1.55	0,85 - 2,82	0.62	0,36 - 1,04
>-1230	62.07	48,4 - 74,5	58.82	32,9 - 81,6	1.51	0,83 - 2,75	0.64	0,38 - 1,08
>-1226	60.34	46,6 - 73,0	58.82	32,9 - 81,6	1.47	0,80 - 2,68	0.67	0,41 - 1,12
>-1213	58.62	44,9 - 71,4	58.82	32,9 - 81,6	1.42	0,78 - 2,61	0.7	0,43 - 1,16
>-1152	58.62	44,9 - 71,4	64.71	38,3 - 85,8	1.66	0,84 - 3,28	0.64	0,40 - 1,02
>-1142	58.62	44,9 - 71,4	70.59	44,0 - 89,7	1.99	0,93 - 4,29	0.59	0,38 - 0,90
>-1114	58.62	44,9 - 71,4	76.47	50,1 - 93,2	2.49	1,03 - 6,03	0.54	0,36 - 0,81
>-1090	56.9	43,2 - 69,8	76.47	50,1 - 93,2	2.42	1,00 - 5,86	0.56	0,38 - 0,84
>-1053	55.17	41,5 - 68,3	76.47	50,1 - 93,2	2.34	0,97 - 5,70	0.59	0,40 - 0,86
>-1031	55.17	41,5 - 68,3	82.35	56,6 - 96,2	3.13	1,09 - 8,96	0.54	0,38 - 0,78
>-1025	55.17	41,5 - 68,3	88.24	63,6 - 98,5	4.69	1,25 - 17,60	0.51	0,36 - 0,71
>-956	53.45	39,9 - 66,7	88.24	63,6 - 98,5	4.54	1,21 - 17,07	0.53	0,38 - 0,73
>-902	51.72	38,2 - 65,0	88.24	63,6 - 98,5	4.4	1,17 - 16,55	0.55	0,40 - 0,75
>-898	50	36,6 - 63,4	88.24	63,6 - 98,5	4.25	1,13 - 16,02	0.57	0,42 - 0,77
>-800	48.28	35,0 - 61,8	88.24	63,6 - 98,5	4.1	1,09 - 15,50	0.59	0,43 - 0,79
>-682	46.55	33,3 - 60,1	88.24	63,6 - 98,5	3.96	1,05 - 14,97	0.61	0,45 - 0,81
>-542	44.83	31,7 - 58,5	88.24	63,6 - 98,5	3.81	1,00 - 14,45	0.63	0,47 - 0,84

>-495	43.1	30,2 - 56,8	88.24	63,6 - 98,5	3.66	0,96 - 13,92	0.64	0,49 - 0,86
>-467	41.38	28,6 - 55,1	88.24	63,6 - 98,5	3.52	0,92 - 13,40	0.66	0,50 - 0,88
>-371	39.66	27,0 - 53,4	88.24	63,6 - 98,5	3.37	0,88 - 12,87	0.68	0,52 - 0,90
>-321	37.93	25,5 - 51,6	88.24	63,6 - 98,5	3.22	0,84 - 12,35	0.7	0,54 - 0,92
>-272	36.21	24,0 - 49,9	88.24	63,6 - 98,5	3.08	0,80 - 11,82	0.72	0,56 - 0,94
>-176	34.48	22,5 - 48,1	88.24	63,6 - 98,5	2.93	0,76 - 11,30	0.74	0,58 - 0,96
>-175	32.76	21,0 - 46,3	88.24	63,6 - 98,5	2.78	0,72 - 10,77	0.76	0,59 - 0,98
>-64	32.76	21,0 - 46,3	94.12	71,3 - 99,9	5.57	0,80 - 38,63	0.71	0,58 - 0,89
>332	31.03	19,5 - 44,5	94.12	71,3 - 99,9	5.28	0,76 - 36,71	0.73	0,59 - 0,90
>399	29.31	18,1 - 42,7	94.12	71,3 - 99,9	4.98	0,71 - 34,78	0.75	0,61 - 0,92
>406	27.59	16,7 - 40,9	94.12	71,3 - 99,9	4.69	0,67 - 32,85	0.77	0,63 - 0,94
>418	25.86	15,3 - 39,0	94.12	71,3 - 99,9	4.4	0,63 - 30,93	0.79	0,65 - 0,96
>758	24.14	13,9 - 37,2	94.12	71,3 - 99,9	4.1	0,58 - 29,00	0.81	0,67 - 0,97
>909	22.41	12,5 - 35,3	94.12	71,3 - 99,9	3.81	0,54 - 27,07	0.82	0,69 - 0,99
>947	20.69	11,2 - 33,4	94.12	71,3 - 99,9	3.52	0,49 - 25,15	0.84	0,71 - 1,01
>1011	18.97	9,9 - 31,4	94.12	71,3 - 99,9	3.22	0,45 - 23,22	0.86	0,72 - 1,02
>1622	17.24	8,6 - 29,4	94.12	71,3 - 99,9	2.93	0,40 - 21,30	0.88	0,74 - 1,04
>1988	15.52	7,3 - 27,4	94.12	71,3 - 99,9	2.64	0,36 - 19,38	0.9	0,76 - 1,06
>2325	13.79	6,1 - 25,4	94.12	71,3 - 99,9	2.34	0,32 - 17,45	0.92	0,78 - 1,07
>2535	12.07	5,0 - 23,3	94.12	71,3 - 99,9	2.05	0,27 - 15,53	0.93	0,80 - 1,09
>3284	10.34	3,9 - 21,2	94.12	71,3 - 99,9	1.76	0,23 - 13,62	0.95	0,82 - 1,10
>6148	8.62	2,9 - 19,0	94.12	71,3 - 99,9	1.47	0,18 - 11,71	0.97	0,84 - 1,12
>6978	6.9	1,9 - 16,7	94.12	71,3 - 99,9	1.17	0,14 - 9,80	0.99	0,86 - 1,14
>7761	5.17	1,1 - 14,4	94.12	71,3 - 99,9	0.88	0,098 - 7,92	1.01	0,88 - 1,15
>8776	5.17	1,1 - 14,4	100	80,5 - 100,0			0.95	0,89 - 1,01
>12673	3.45	0,4 - 11,9	100	80,5 - 100,0			0.97	0,92 - 1,01
>19547	1.72	0,04 - 9,2	100	80,5 - 100,0			0.98	0,95 - 1,02
>33791	0	0,0 - 6,2	100	80,5 - 100,0			1	1,00 - 1,00

Monday, September 22, 2025 16:56 - MedCalc® version 23.3.7

6D

frontiers

ROC curve

Variable	Peptid_7
Classification variable	status

Sample size	75
Positive group [a]	58 (77,33%)
Negative group [b]	17 (22,67%)

status = 1
[b] status = 0

Disease prevalence (%)	unknown

Area under the ROC curve (AUC)

Area under the ROC curve (AUC)	0.597
Standard Error [a]	0.0831
95% Confidence interval [b]	0,478 to 0,709
z statistic	1.172
Significance level P (Area=0.5)	0.2412

[a] DeLong et al., 1988
[b] Binomial exact

Youden index

Youden index J	0.2677
Associated criterion	≤-1256
Sensitivity	62.07
Specificity	64.71

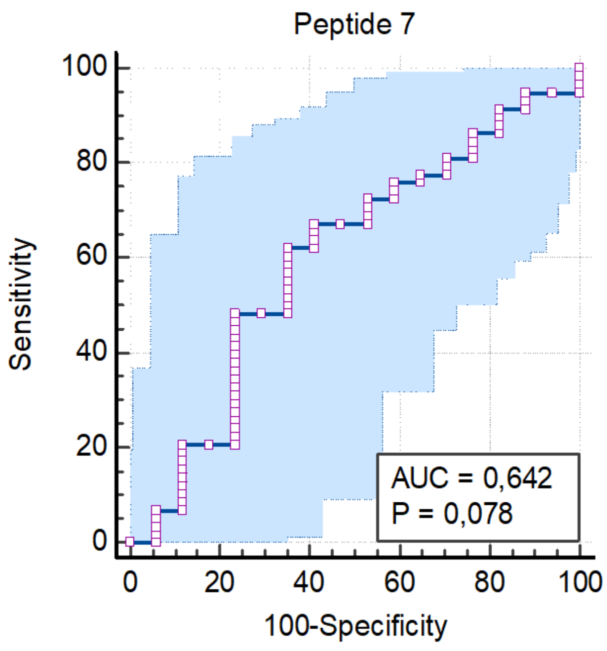

Criterion	Sensitivity	95% CI	Specificity	95% CI	+LR	95% CI	-LR	95% CI
<-56022	0	0,0 - 6,2	100	80,5 - 100,0			1	1,00 - 1,00
≤-56022	0	0,0 - 6,2	94.12	71,3 - 99,9	0		1.06	0,94 - 1,20
≤-15323	1.72	0,04 - 9,2	94.12	71,3 - 99,9	0.29	0,019 - 4,44	1.04	0,92 - 1,18
≤-9303	3.45	0,4 - 11,9	94.12	71,3 - 99,9	0.59	0,057 - 6,08	1.03	0,90 - 1,17
≤-8149	5.17	1,1 - 14,4	94.12	71,3 - 99,9	0.88	0,098 - 7,92	1.01	0,88 - 1,15
≤-7864	6.9	1,9 - 16,7	94.12	71,3 - 99,9	1.17	0,14 - 9,80	0.99	0,86 - 1,14
≤-7241	6.9	1,9 - 16,7	88.24	63,6 - 98,5	0.59	0,12 - 2,93	1.06	0,88 - 1,27
≤-6801	8.62	2,9 - 19,0	88.24	63,6 - 98,5	0.73	0,16 - 3,45	1.04	0,86 - 1,25
≤-6070	10.34	3,9 - 21,2	88.24	63,6 - 98,5	0.88	0,19 - 3,97	1.02	0,84 - 1,23
≤-5351	12.07	5,0 - 23,3	88.24	63,6 - 98,5	1.03	0,23 - 4,49	1	0,82 - 1,21
≤-5270	13.79	6,1 - 25,4	88.24	63,6 - 98,5	1.17	0,27 - 5,01	0.98	0,80 - 1,20
≤-4857	15.52	7,3 - 27,4	88.24	63,6 - 98,5	1.32	0,31 - 5,53	0.96	0,78 - 1,18
≤-4300	17.24	8,6 - 29,4	88.24	63,6 - 98,5	1.47	0,35 - 6,06	0.94	0,76 - 1,16
≤-4009	18.97	9,9 - 31,4	88.24	63,6 - 98,5	1.61	0,40 - 6,58	0.92	0,74 - 1,14
≤-3784	20.69	11,2 - 33,4	88.24	63,6 - 98,5	1.76	0,44 - 7,10	0.9	0,72 - 1,12
≤-3671	20.69	11,2 - 33,4	82.35	56,6 - 96,2	1.17	0,37 - 3,68	0.96	0,75 - 1,24
≤-3665	20.69	11,2 - 33,4	76.47	50,1 - 93,2	0.88	0,33 - 2,38	1.04	0,77 - 1,39
≤-3575	22.41	12,5 - 35,3	76.47	50,1 - 93,2	0.95	0,36 - 2,54	1.01	0,75 - 1,37
≤-3092	24.14	13,9 - 37,2	76.47	50,1 - 93,2	1.03	0,39 - 2,71	0.99	0,73 - 1,34
≤-2963	25.86	15,3 - 39,0	76.47	50,1 - 93,2	1.1	0,42 - 2,87	0.97	0,72 - 1,31
≤-2859	27.59	16,7 - 40,9	76.47	50,1 - 93,2	1.17	0,45 - 3,04	0.95	0,70 - 1,29
≤-2732	29.31	18,1 - 42,7	76.47	50,1 - 93,2	1.25	0,48 - 3,21	0.92	0,68 - 1,26
≤-2731	31.03	19,5 - 44,5	76.47	50,1 - 93,2	1.32	0,52 - 3,37	0.9	0,66 - 1,24
≤-2493	32.76	21,0 - 46,3	76.47	50,1 - 93,2	1.39	0,55 - 3,54	0.88	0,64 - 1,21
≤-2483	34.48	22,5 - 48,1	76.47	50,1 - 93,2	1.47	0,58 - 3,71	0.86	0,62 - 1,18

frontiers

≤-2305	36.21	24,0 - 49,9	76.47	50,1 - 93,2	1.54	0,61 - 3,87	0.83	0,60 - 1,16
≤-2174	37.93	25,5 - 51,6	76.47	50,1 - 93,2	1.61	0,64 - 4,04	0.81	0,58 - 1,13
≤-2117	39.66	27,0 - 53,4	76.47	50,1 - 93,2	1.69	0,68 - 4,20	0.79	0,56 - 1,10
≤-2113	41.38	28,6 - 55,1	76.47	50,1 - 93,2	1.76	0,71 - 4,37	0.77	0,55 - 1,08
≤-2076	43.1	30,2 - 56,8	76.47	50,1 - 93,2	1.83	0,74 - 4,54	0.74	0,53 - 1,05
≤-2029	44.83	31,7 - 58,5	76.47	50,1 - 93,2	1.91	0,77 - 4,70	0.72	0,51 - 1,03
≤-1990	46.55	33,3 - 60,1	76.47	50,1 - 93,2	1.98	0,80 - 4,87	0.7	0,49 - 1,00
≤-1947	48.28	35,0 - 61,8	76.47	50,1 - 93,2	2.05	0,84 - 5,03	0.68	0,47 - 0,97
≤-1882	48.28	35,0 - 61,8	70.59	44,0 - 89,7	1.64	0,75 - 3,59	0.73	0,49 - 1,09
≤-1507	48.28	35,0 - 61,8	64.71	38,3 - 85,8	1.37	0,68 - 2,75	0.8	0,52 - 1,23
≤-1492	50	36,6 - 63,4	64.71	38,3 - 85,8	1.42	0,71 - 2,83	0.77	0,50 - 1,19
≤-1458	51.72	38,2 - 65,0	64.71	38,3 - 85,8	1.47	0,74 - 2,92	0.75	0,48 - 1,16
≤-1445	53.45	39,9 - 66,7	64.71	38,3 - 85,8	1.51	0,76 - 3,01	0.72	0,46 - 1,12
≤-1417	55.17	41,5 - 68,3	64.71	38,3 - 85,8	1.56	0,79 - 3,10	0.69	0,44 - 1,09
≤-1351	56.9	43,2 - 69,8	64.71	38,3 - 85,8	1.61	0,82 - 3,19	0.67	0,42 - 1,05
≤-1339	58.62	44,9 - 71,4	64.71	38,3 - 85,8	1.66	0,84 - 3,28	0.64	0,40 - 1,02
≤-1277	60.34	46,6 - 73,0	64.71	38,3 - 85,8	1.71	0,87 - 3,36	0.61	0,38 - 0,98
≤-1256	62.07	48,4 - 74,5	64.71	38,3 - 85,8	1.76	0,90 - 3,45	0.59	0,36 - 0,95
≤-1156	62.07	48,4 - 74,5	58.82	32,9 - 81,6	1.51	0,83 - 2,75	0.64	0,38 - 1,08
≤-1067	63.79	50,1 - 76,0	58.82	32,9 - 81,6	1.55	0,85 - 2,82	0.62	0,36 - 1,04
≤-1009	65.52	51,9 - 77,5	58.82	32,9 - 81,6	1.59	0,87 - 2,89	0.59	0,34 - 1,00
≤-991	67.24	53,7 - 79,0	58.82	32,9 - 81,6	1.63	0,90 - 2,96	0.56	0,32 - 0,96
≤-894	67.24	53,7 - 79,0	52.94	27,8 - 77,0	1.43	0,84 - 2,44	0.62	0,35 - 1,11
≤-827	67.24	53,7 - 79,0	47.06	23,0 - 72,2	1.27	0,78 - 2,06	0.7	0,37 - 1,30
≤-796	68.97	55,5 - 80,5	47.06	23,0 - 72,2	1.3	0,81 - 2,11	0.66	0,35 - 1,24
≤-733	70.69	57,3 - 81,9	47.06	23,0 - 72,2	1.34	0,83 - 2,15	0.62	0,33 - 1,19

≤-692	72.41	59,1 - 83,3	47.06	23,0 - 72,2	1.37	0,85 - 2,20	0.59	0,30 - 1,13
≤-625	72.41	59,1 - 83,3	41.18	18,4 - 67,1	1.23	0,80 - 1,89	0.67	0,33 - 1,36
≤-557	74.14	61,0 - 84,7	41.18	18,4 - 67,1	1.26	0,82 - 1,93	0.63	0,31 - 1,29
≤-468	75.86	62,8 - 86,1	41.18	18,4 - 67,1	1.29	0,84 - 1,97	0.59	0,28 - 1,21
≤-298	75.86	62,8 - 86,1	35.29	14,2 - 61,7	1.17	0,80 - 1,71	0.68	0,31 - 1,51
≤-240	77.59	64,7 - 87,5	35.29	14,2 - 61,7	1.2	0,82 - 1,75	0.64	0,28 - 1,42
≤-62	77.59	64,7 - 87,5	29.41	10,3 - 56,0	1.1	0,79 - 1,54	0.76	0,32 - 1,83
≤-29	79.31	66,6 - 88,8	29.41	10,3 - 56,0	1.12	0,80 - 1,57	0.7	0,29 - 1,72
≤-16	81.03	68,6 - 90,1	29.41	10,3 - 56,0	1.15	0,82 - 1,60	0.64	0,26 - 1,60
≤178	81.03	68,6 - 90,1	23.53	6,8 - 49,9	1.06	0,79 - 1,42	0.81	0,29 - 2,21
≤372	82.76	70,6 - 91,4	23.53	6,8 - 49,9	1.08	0,81 - 1,44	0.73	0,26 - 2,04
≤414	84.48	72,6 - 92,7	23.53	6,8 - 49,9	1.1	0,83 - 1,47	0.66	0,23 - 1,88
≤429	86.21	74,6 - 93,9	23.53	6,8 - 49,9	1.13	0,85 - 1,50	0.59	0,20 - 1,71
≤567	86.21	74,6 - 93,9	17.65	3,8 - 43,4	1.05	0,82 - 1,33	0.78	0,23 - 2,63
≤672	87.93	76,7 - 95,0	17.65	3,8 - 43,4	1.07	0,84 - 1,36	0.68	0,20 - 2,36
≤753	89.66	78,8 - 96,1	17.65	3,8 - 43,4	1.09	0,86 - 1,38	0.59	0,16 - 2,10
≤1417	91.38	81,0 - 97,1	17.65	3,8 - 43,4	1.11	0,88 - 1,40	0.49	0,13 - 1,84
≤1849	91.38	81,0 - 97,1	11.76	1,5 - 36,4	1.04	0,86 - 1,25	0.73	0,16 - 3,45
≤2025	93.1	83,3 - 98,1	11.76	1,5 - 36,4	1.06	0,88 - 1,27	0.59	0,12 - 2,93
≤2754	94.83	85,6 - 98,9	11.76	1,5 - 36,4	1.07	0,89 - 1,29	0.44	0,080 - 2,42
≤3691	94.83	85,6 - 98,9	5.88	0,1 - 28,7	1.01	0,88 - 1,15	0.88	0,098 - 7,92
≤4046	94.83	85,6 - 98,9	0	0,0 - 19,5	0.95	0,89 - 1,01		
≤17889	96.55	88,1 - 99,6	0	0,0 - 19,5	0.97	0,92 - 1,01		
≤21859	98.28	90,8 - 100,0	0	0,0 - 19,5	0.98	0,95 - 1,02		
≤23694	100	93,8 - 100,0	0	0,0 - 19,5	1	1,00 - 1,00		

Monday, September 22, 2025 16:56 - MedCalc® version 23.3.7

6E

ROC curve

Variable	Peptid_8
Classification variable	status

Sample size	75
Positive group [a]	58 (77,33%)
Negative group [b]	17 (22,67%)

status = 1

[b] status = 0

Disease prevalence (%)	unknown

Area under the ROC curve (AUC)

Area under the ROC curve (AUC)	0.712
Standard Error [a]	0.0679
95% Confidence interval [b]	0,596 to 0,811
z statistic	3.121
Significance level P (Area=0.5)	0.0018

[a] DeLong et al., 1988

[b] Binomial exact

Youden index

Youden index J	0.4513
Associated criterion	≤-1731
Sensitivity	56.9
Specificity	88.24

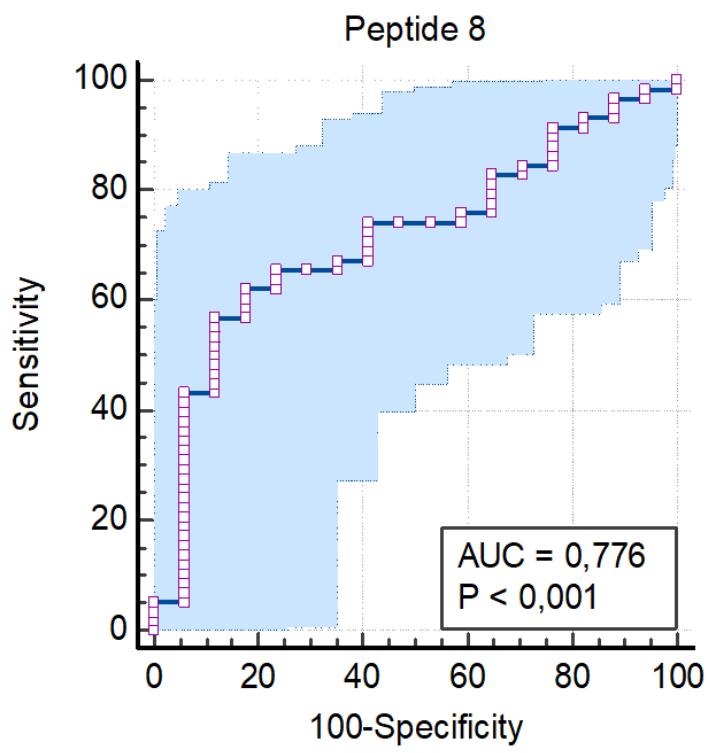

Criterion	Sensitivity	95% CI	Specificity	95% CI	+LR	95% CI	-LR	95% CI
<-14407	0	0,0 - 6,2	100	80,5 - 100,0			1	1,00 - 1,00
≤-14407	1.72	0,04 - 9,2	100	80,5 - 100,0			0.98	0,95 - 1,02
≤-8515	3.45	0,4 - 11,9	100	80,5 - 100,0			0.97	0,92 - 1,01
≤-7140	5.17	1,1 - 14,4	100	80,5 - 100,0			0.95	0,89 - 1,01
≤-6248	5.17	1,1 - 14,4	94.12	71,3 - 99,9	0.88	0,098 - 7,92	1.01	0,88 - 1,15
≤-6057	6.9	1,9 - 16,7	94.12	71,3 - 99,9	1.17	0,14 - 9,80	0.99	0,86 - 1,14
≤-5814	8.62	2,9 - 19,0	94.12	71,3 - 99,9	1.47	0,18 - 11,71	0.97	0,84 - 1,12
≤-5797	10.34	3,9 - 21,2	94.12	71,3 - 99,9	1.76	0,23 - 13,62	0.95	0,82 - 1,10
≤-4541	12.07	5,0 - 23,3	94.12	71,3 - 99,9	2.05	0,27 - 15,53	0.93	0,80 - 1,09
≤-4349	13.79	6,1 - 25,4	94.12	71,3 - 99,9	2.34	0,32 - 17,45	0.92	0,78 - 1,07
≤-4128	15.52	7,3 - 27,4	94.12	71,3 - 99,9	2.64	0,36 - 19,38	0.9	0,76 - 1,06
≤-3830	17.24	8,6 - 29,4	94.12	71,3 - 99,9	2.93	0,40 - 21,30	0.88	0,74 - 1,04
≤-3496	18.97	9,9 - 31,4	94.12	71,3 - 99,9	3.22	0,45 - 23,22	0.86	0,72 - 1,02
≤-3139	20.69	11,2 - 33,4	94.12	71,3 - 99,9	3.52	0,49 - 25,15	0.84	0,71 - 1,01
≤-2841	22.41	12,5 - 35,3	94.12	71,3 - 99,9	3.81	0,54 - 27,07	0.82	0,69 - 0,99
≤-2792	24.14	13,9 - 37,2	94.12	71,3 - 99,9	4.1	0,58 - 29,00	0.81	0,67 - 0,97
≤-2631	25.86	15,3 - 39,0	94.12	71,3 - 99,9	4.4	0,63 - 30,93	0.79	0,65 - 0,96
≤-2464	27.59	16,7 - 40,9	94.12	71,3 - 99,9	4.69	0,67 - 32,85	0.77	0,63 - 0,94
≤-2423	29.31	18,1 - 42,7	94.12	71,3 - 99,9	4.98	0,71 - 34,78	0.75	0,61 - 0,92
≤-2420	31.03	19,5 - 44,5	94.12	71,3 - 99,9	5.28	0,76 - 36,71	0.73	0,59 - 0,90
≤-2374	32.76	21,0 - 46,3	94.12	71,3 - 99,9	5.57	0,80 - 38,63	0.71	0,58 - 0,89
≤-2357	34.48	22,5 - 48,1	94.12	71,3 - 99,9	5.86	0,85 - 40,56	0.7	0,56 - 0,87
≤-2294	36.21	24,0 - 49,9	94.12	71,3 - 99,9	6.16	0,89 - 42,49	0.68	0,54 - 0,85
≤-2260	37.93	25,5 - 51,6	94.12	71,3 - 99,9	6.45	0,94 - 44,42	0.66	0,52 - 0,83

≤-2220	39.66	27,0 - 53,4	94.12	71,3 - 99,9	6.74	0,98 - 46,34	0.64	0,50 - 0,82
≤-2179	41.38	28,6 - 55,1	94.12	71,3 - 99,9	7.03	1,03 - 48,27	0.62	0,49 - 0,80
≤-2095	43.1	30,2 - 56,8	94.12	71,3 - 99,9	7.33	1,07 - 50,20	0.6	0,47 - 0,78
≤-1923	43.1	30,2 - 56,8	88.24	63,6 - 98,5	3.66	0,96 - 13,92	0.64	0,49 - 0,86
≤-1914	44.83	31,7 - 58,5	88.24	63,6 - 98,5	3.81	1,00 - 14,45	0.63	0,47 - 0,84
≤-1883	46.55	33,3 - 60,1	88.24	63,6 - 98,5	3.96	1,05 - 14,97	0.61	0,45 - 0,81
≤-1868	48.28	35,0 - 61,8	88.24	63,6 - 98,5	4.1	1,09 - 15,50	0.59	0,43 - 0,79
≤-1855	50	36,6 - 63,4	88.24	63,6 - 98,5	4.25	1,13 - 16,02	0.57	0,42 - 0,77
≤-1833	51.72	38,2 - 65,0	88.24	63,6 - 98,5	4.4	1,17 - 16,55	0.55	0,40 - 0,75
≤-1830	53.45	39,9 - 66,7	88.24	63,6 - 98,5	4.54	1,21 - 17,07	0.53	0,38 - 0,73
≤-1810	55.17	41,5 - 68,3	88.24	63,6 - 98,5	4.69	1,25 - 17,60	0.51	0,36 - 0,71
≤-1731	56.9	43,2 - 69,8	88.24	63,6 - 98,5	4.84	1,29 - 18,12	0.49	0,35 - 0,69
≤-1730	56.9	43,2 - 69,8	82.35	56,6 - 96,2	3.22	1,13 - 9,22	0.52	0,36 - 0,76
≤-1719	58.62	44,9 - 71,4	82.35	56,6 - 96,2	3.32	1,16 - 9,49	0.5	0,34 - 0,73
≤-1711	60.34	46,6 - 73,0	82.35	56,6 - 96,2	3.42	1,20 - 9,75	0.48	0,33 - 0,71
≤-1677	62.07	48,4 - 74,5	82.35	56,6 - 96,2	3.52	1,24 - 10,02	0.46	0,31 - 0,68
≤-1670	62.07	48,4 - 74,5	76.47	50,1 - 93,2	2.64	1,09 - 6,36	0.5	0,33 - 0,76
≤-1655	63.79	50,1 - 76,0	76.47	50,1 - 93,2	2.71	1,13 - 6,53	0.47	0,31 - 0,73
≤-1652	65.52	51,9 - 77,5	76.47	50,1 - 93,2	2.78	1,16 - 6,69	0.45	0,29 - 0,70
≤-1641	65.52	51,9 - 77,5	70.59	44,0 - 89,7	2.23	1,04 - 4,76	0.49	0,31 - 0,78
≤-1620	65.52	51,9 - 77,5	64.71	38,3 - 85,8	1.86	0,95 - 3,63	0.53	0,32 - 0,88
≤-1610	67.24	53,7 - 79,0	64.71	38,3 - 85,8	1.91	0,98 - 3,72	0.51	0,30 - 0,84
≤-1588	67.24	53,7 - 79,0	58.82	32,9 - 81,6	1.63	0,90 - 2,96	0.56	0,32 - 0,96
≤-1569	68.97	55,5 - 80,5	58.82	32,9 - 81,6	1.67	0,92 - 3,03	0.53	0,30 - 0,92
≤-1547	70.69	57,3 - 81,9	58.82	32,9 - 81,6	1.72	0,95 - 3,10	0.5	0,28 - 0,88

≤-1514	72.41	59,1 - 83,3	58.82	32,9 - 81,6	1.76	0,97 - 3,17	0.47	0,26 - 0,83
≤-1492	74.14	61,0 - 84,7	58.82	32,9 - 81,6	1.8	1,00 - 3,24	0.44	0,24 - 0,79
≤-1435	74.14	61,0 - 84,7	52.94	27,8 - 77,0	1.58	0,93 - 2,67	0.49	0,26 - 0,91
≤-1403	74.14	61,0 - 84,7	47.06	23,0 - 72,2	1.4	0,87 - 2,25	0.55	0,28 - 1,07
≤-1375	74.14	61,0 - 84,7	41.18	18,4 - 67,1	1.26	0,82 - 1,93	0.63	0,31 - 1,29
≤-1248	75.86	62,8 - 86,1	41.18	18,4 - 67,1	1.29	0,84 - 1,97	0.59	0,28 - 1,21
≤-1198	75.86	62,8 - 86,1	35.29	14,2 - 61,7	1.17	0,80 - 1,71	0.68	0,31 - 1,51
≤-1188	77.59	64,7 - 87,5	35.29	14,2 - 61,7	1.2	0,82 - 1,75	0.64	0,28 - 1,42
≤-1168	79.31	66,6 - 88,8	35.29	14,2 - 61,7	1.23	0,84 - 1,78	0.59	0,26 - 1,33
≤-1112	81.03	68,6 - 90,1	35.29	14,2 - 61,7	1.25	0,86 - 1,82	0.54	0,23 - 1,24
≤-1074	82.76	70,6 - 91,4	35.29	14,2 - 61,7	1.28	0,88 - 1,85	0.49	0,21 - 1,15
≤-1071	82.76	70,6 - 91,4	29.41	10,3 - 56,0	1.17	0,84 - 1,63	0.59	0,23 - 1,48
≤-966	84.48	72,6 - 92,7	29.41	10,3 - 56,0	1.2	0,86 - 1,66	0.53	0,20 - 1,36
≤-714	84.48	72,6 - 92,7	23.53	6,8 - 49,9	1.1	0,83 - 1,47	0.66	0,23 - 1,88
≤-556	86.21	74,6 - 93,9	23.53	6,8 - 49,9	1.13	0,85 - 1,50	0.59	0,20 - 1,71
≤-371	87.93	76,7 - 95,0	23.53	6,8 - 49,9	1.15	0,87 - 1,52	0.51	0,17 - 1,55
≤112	89.66	78,8 - 96,1	23.53	6,8 - 49,9	1.17	0,89 - 1,55	0.44	0,14 - 1,38
≤664	91.38	81,0 - 97,1	23.53	6,8 - 49,9	1.19	0,91 - 1,57	0.37	0,11 - 1,21
≤700	91.38	81,0 - 97,1	17.65	3,8 - 43,4	1.11	0,88 - 1,40	0.49	0,13 - 1,84
≤1642	93.1	83,3 - 98,1	17.65	3,8 - 43,4	1.13	0,90 - 1,42	0.39	0,097 - 1,58
≤1789	93.1	83,3 - 98,1	11.76	1,5 - 36,4	1.06	0,88 - 1,27	0.59	0,12 - 2,93
≤2386	94.83	85,6 - 98,9	11.76	1,5 - 36,4	1.07	0,89 - 1,29	0.44	0,080 - 2,42
≤2557	96.55	88,1 - 99,6	11.76	1,5 - 36,4	1.09	0,91 - 1,31	0.29	0,045 - 1,93
≤3783	96.55	88,1 - 99,6	5.88	0,1 - 28,7	1.03	0,90 - 1,17	0.59	0,057 - 6,08
≤4899	98.28	90,8 - 100,0	5.88	0,1 - 28,7	1.04	0,92 - 1,18	0.29	0,019 - 4,44
≤9068	98.28	90,8 - 100,0	0	0,0 - 19,5	0.98	0,95 - 1,02		
≤16705	100	93,8 - 100,0	0	0,0 - 19,5	1	1,00 - 1,00		

Monday, September 22, 2025 16:57 - MedCalc® version 23.3.7

6F

ROC curve

Variable	Peptid_11
Classification variable	status

Sample size	75
Positive group [a]	58 (77,33%)
Negative group [b]	17 (22,67%)

status = 1

[b] status = 0

Disease prevalence (%)	unknown

Area under the ROC curve (AUC)

Area under the ROC curve (AUC)	0.579
Standard Error [a]	0.0745
95% Confidence interval [b]	0,459 to 0,692
z statistic	1.062
Significance level P (Area=0.5)	0.2884

[a] DeLong et al., 1988

[b] Binomial exact

Youden index

Youden index J	0.289
Associated criterion	>-836
Sensitivity	46.55
Specificity	82.35

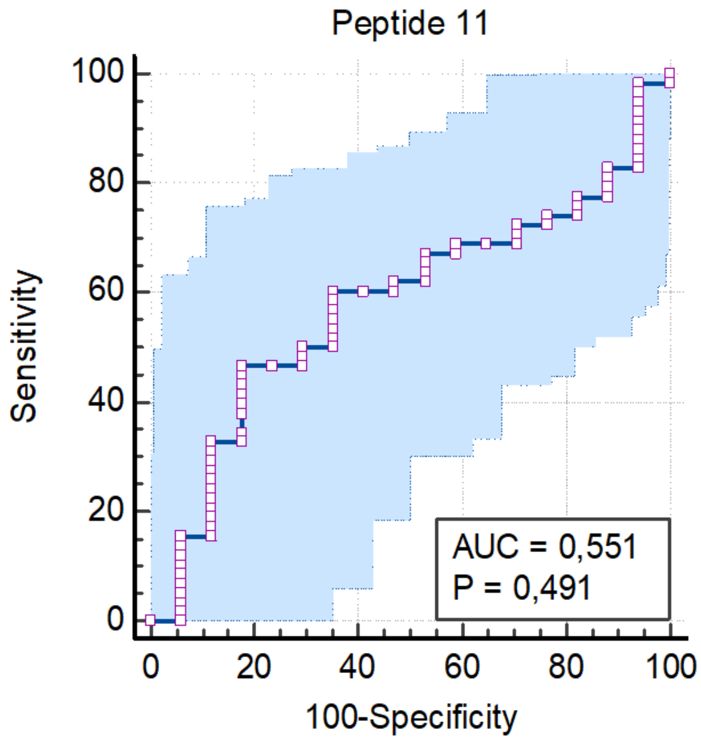

Criterion	Sensitivity	95% CI	Specificity	95% CI	+LR	95% CI	-LR	95% CI
≥-19059	100	93,8 - 100,0	0	0,0 - 19,5	1	1,00 - 1,00		
>-19059	98.28	90,8 - 100,0	0	0,0 - 19,5	0.98	0,95 - 1,02		
>-9801	98.28	90,8 - 100,0	5.88	0,1 - 28,7	1.04	0,92 - 1,18	0.29	0,019 - 4,44
>-3822	96.55	88,1 - 99,6	5.88	0,1 - 28,7	1.03	0,90 - 1,17	0.59	0,057 - 6,08
>-3777	94.83	85,6 - 98,9	5.88	0,1 - 28,7	1.01	0,88 - 1,15	0.88	0,098 - 7,92
>-3660	93.1	83,3 - 98,1	5.88	0,1 - 28,7	0.99	0,86 - 1,14	1.17	0,14 - 9,80
>-3359	91.38	81,0 - 97,1	5.88	0,1 - 28,7	0.97	0,84 - 1,12	1.47	0,18 - 11,71
>-3034	89.66	78,8 - 96,1	5.88	0,1 - 28,7	0.95	0,82 - 1,10	1.76	0,23 - 13,62
>-2407	87.93	76,7 - 95,0	5.88	0,1 - 28,7	0.93	0,80 - 1,09	2.05	0,27 - 15,53
>-2203	86.21	74,6 - 93,9	5.88	0,1 - 28,7	0.92	0,78 - 1,07	2.34	0,32 - 17,45
>-1928	84.48	72,6 - 92,7	5.88	0,1 - 28,7	0.9	0,76 - 1,06	2.64	0,36 - 19,38
>-1926	82.76	70,6 - 91,4	5.88	0,1 - 28,7	0.88	0,74 - 1,04	2.93	0,40 - 21,30
>-1773	82.76	70,6 - 91,4	11.76	1,5 - 36,4	0.94	0,76 - 1,16	1.47	0,35 - 6,06
>-1744	81.03	68,6 - 90,1	11.76	1,5 - 36,4	0.92	0,74 - 1,14	1.61	0,40 - 6,58
>-1573	79.31	66,6 - 88,8	11.76	1,5 - 36,4	0.9	0,72 - 1,12	1.76	0,44 - 7,10
>-1555	77.59	64,7 - 87,5	11.76	1,5 - 36,4	0.88	0,70 - 1,10	1.91	0,48 - 7,63
>-1539	77.59	64,7 - 87,5	17.65	3,8 - 43,4	0.94	0,73 - 1,22	1.27	0,41 - 3,94
>-1534	75.86	62,8 - 86,1	17.65	3,8 - 43,4	0.92	0,71 - 1,20	1.37	0,44 - 4,21
>-1499	74.14	61,0 - 84,7	17.65	3,8 - 43,4	0.9	0,69 - 1,18	1.47	0,48 - 4,47
>-1454	74.14	61,0 - 84,7	23.53	6,8 - 49,9	0.97	0,72 - 1,31	1.1	0,42 - 2,87
>-1451	72.41	59,1 - 83,3	23.53	6,8 - 49,9	0.95	0,70 - 1,29	1.17	0,45 - 3,04
>-1435	72.41	59,1 - 83,3	29.41	10,3 - 56,0	1.03	0,73 - 1,45	0.94	0,40 - 2,19
>-1429	70.69	57,3 - 81,9	29.41	10,3 - 56,0	1	0,71 - 1,42	1	0,43 - 2,30

>-1376	68.97	55,5 - 80,5	29.41	10,3 - 56,0	0.98	0,69 - 1,39	1.06	0,46 - 2,42
>-1356	68.97	55,5 - 80,5	35.29	14,2 - 61,7	1.07	0,72 - 1,58	0.88	0,42 - 1,86
>-1354	68.97	55,5 - 80,5	41.18	18,4 - 67,1	1.17	0,76 - 1,81	0.75	0,38 - 1,50
>-1342	67.24	53,7 - 79,0	41.18	18,4 - 67,1	1.14	0,74 - 1,77	0.8	0,40 - 1,57
>-1328	67.24	53,7 - 79,0	47.06	23,0 - 72,2	1.27	0,78 - 2,06	0.7	0,37 - 1,30
>-1319	65.52	51,9 - 77,5	47.06	23,0 - 72,2	1.24	0,76 - 2,01	0.73	0,40 - 1,36
>-1284	63.79	50,1 - 76,0	47.06	23,0 - 72,2	1.2	0,74 - 1,96	0.77	0,42 - 1,41
>-1278	62.07	48,4 - 74,5	47.06	23,0 - 72,2	1.17	0,72 - 1,92	0.81	0,44 - 1,47
>-1268	62.07	48,4 - 74,5	52.94	27,8 - 77,0	1.32	0,77 - 2,27	0.72	0,41 - 1,25
>-1236	60.34	46,6 - 73,0	52.94	27,8 - 77,0	1.28	0,74 - 2,21	0.75	0,43 - 1,30
>-1182	60.34	46,6 - 73,0	58.82	32,9 - 81,6	1.47	0,80 - 2,68	0.67	0,41 - 1,12
>-1121	60.34	46,6 - 73,0	64.71	38,3 - 85,8	1.71	0,87 - 3,36	0.61	0,38 - 0,98
>-1095	58.62	44,9 - 71,4	64.71	38,3 - 85,8	1.66	0,84 - 3,28	0.64	0,40 - 1,02
>-1024	56.9	43,2 - 69,8	64.71	38,3 - 85,8	1.61	0,82 - 3,19	0.67	0,42 - 1,05
>-991	55.17	41,5 - 68,3	64.71	38,3 - 85,8	1.56	0,79 - 3,10	0.69	0,44 - 1,09
>-955	53.45	39,9 - 66,7	64.71	38,3 - 85,8	1.51	0,76 - 3,01	0.72	0,46 - 1,12
>-941	51.72	38,2 - 65,0	64.71	38,3 - 85,8	1.47	0,74 - 2,92	0.75	0,48 - 1,16
>-917	50	36,6 - 63,4	64.71	38,3 - 85,8	1.42	0,71 - 2,83	0.77	0,50 - 1,19
>-904	50	36,6 - 63,4	70.59	44,0 - 89,7	1.7	0,78 - 3,71	0.71	0,47 - 1,06
>-892	48.28	35,0 - 61,8	70.59	44,0 - 89,7	1.64	0,75 - 3,59	0.73	0,49 - 1,09
>-862	46.55	33,3 - 60,1	70.59	44,0 - 89,7	1.58	0,72 - 3,47	0.76	0,51 - 1,12
>-837	46.55	33,3 - 60,1	76.47	50,1 - 93,2	1.98	0,80 - 4,87	0.7	0,49 - 1,00
>-836	46.55	33,3 - 60,1	82.35	56,6 - 96,2	2.64	0,91 - 7,64	0.65	0,47 - 0,90
>-750	44.83	31,7 - 58,5	82.35	56,6 - 96,2	2.54	0,87 - 7,38	0.67	0,49 - 0,92
>-726	43.1	30,2 - 56,8	82.35	56,6 - 96,2	2.44	0,84 - 7,11	0.69	0,50 - 0,95
>-643	41.38	28,6 - 55,1	82.35	56,6 - 96,2	2.34	0,80 - 6,85	0.71	0,52 - 0,97

>-573	39.66	27,0 - 53,4	82.35	56,6 - 96,2	2.25	0,77 - 6,58	0.73	0,54 - 0,99
>-536	37.93	25,5 - 51,6	82.35	56,6 - 96,2	2.15	0,73 - 6,32	0.75	0,56 - 1,02
>-501	34.48	22,5 - 48,1	82.35	56,6 - 96,2	1.95	0,66 - 5,79	0.8	0,60 - 1,06
>-439	32.76	21,0 - 46,3	82.35	56,6 - 96,2	1.86	0,62 - 5,53	0.82	0,61 - 1,08
>-388	32.76	21,0 - 46,3	88.24	63,6 - 98,5	2.78	0,72 - 10,77	0.76	0,59 - 0,98
>-386	31.03	19,5 - 44,5	88.24	63,6 - 98,5	2.64	0,68 - 10,25	0.78	0,61 - 1,00
>-233	29.31	18,1 - 42,7	88.24	63,6 - 98,5	2.49	0,64 - 9,72	0.8	0,63 - 1,02
>-218	27.59	16,7 - 40,9	88.24	63,6 - 98,5	2.34	0,60 - 9,20	0.82	0,65 - 1,04
>-63	25.86	15,3 - 39,0	88.24	63,6 - 98,5	2.2	0,56 - 8,68	0.84	0,67 - 1,06
>-30	24.14	13,9 - 37,2	88.24	63,6 - 98,5	2.05	0,52 - 8,15	0.86	0,69 - 1,08
>220	22.41	12,5 - 35,3	88.24	63,6 - 98,5	1.91	0,48 - 7,63	0.88	0,70 - 1,10
>293	20.69	11,2 - 33,4	88.24	63,6 - 98,5	1.76	0,44 - 7,10	0.9	0,72 - 1,12
>309	18.97	9,9 - 31,4	88.24	63,6 - 98,5	1.61	0,40 - 6,58	0.92	0,74 - 1,14
>397	17.24	8,6 - 29,4	88.24	63,6 - 98,5	1.47	0,35 - 6,06	0.94	0,76 - 1,16
>652	15.52	7,3 - 27,4	88.24	63,6 - 98,5	1.32	0,31 - 5,53	0.96	0,78 - 1,18
>741	15.52	7,3 - 27,4	94.12	71,3 - 99,9	2.64	0,36 - 19,38	0.9	0,76 - 1,06
>789	13.79	6,1 - 25,4	94.12	71,3 - 99,9	2.34	0,32 - 17,45	0.92	0,78 - 1,07
>1261	12.07	5,0 - 23,3	94.12	71,3 - 99,9	2.05	0,27 - 15,53	0.93	0,80 - 1,09
>1619	10.34	3,9 - 21,2	94.12	71,3 - 99,9	1.76	0,23 - 13,62	0.95	0,82 - 1,10
>1866	8.62	2,9 - 19,0	94.12	71,3 - 99,9	1.47	0,18 - 11,71	0.97	0,84 - 1,12
>4683	6.9	1,9 - 16,7	94.12	71,3 - 99,9	1.17	0,14 - 9,80	0.99	0,86 - 1,14
>6750	5.17	1,1 - 14,4	94.12	71,3 - 99,9	0.88	0,098 - 7,92	1.01	0,88 - 1,15
>7141	3.45	0,4 - 11,9	94.12	71,3 - 99,9	0.59	0,057 - 6,08	1.03	0,90 - 1,17
>9176	1.72	0,04 - 9,2	94.12	71,3 - 99,9	0.29	0,019 - 4,44	1.04	0,92 - 1,18
>16994	0	0,0 - 6,2	94.12	71,3 - 99,9	0		1.06	0,94 - 1,20
>19585	0	0,0 - 6,2	100	80,5 - 100,0			1	1,00 - 1,00

Monday, September 22, 2025 16:57 - MedCalc® version 23.3.7

6G

ROC curve

Variable	Peptid_13
Classification variable	status

Sample size	75
Positive group [a]	58 (77,33%)
Negative group [b]	17 (22,67%)

status = 1

[b] status = 0

Disease prevalence (%)	unknown

Area under the ROC curve (AUC)

Area under the ROC curve (AUC)	0.552
Standard Error [a]	0.0735
95% Confidence interval [b]	0,432 to 0,667
z statistic	0.704
Significance level P (Area=0.5)	0.4813

[a] DeLong et al., 1988

[b] Binomial exact

Youden index

Youden index J	0.1998
Associated criterion	>2574
Sensitivity	25.86
Specificity	94.12

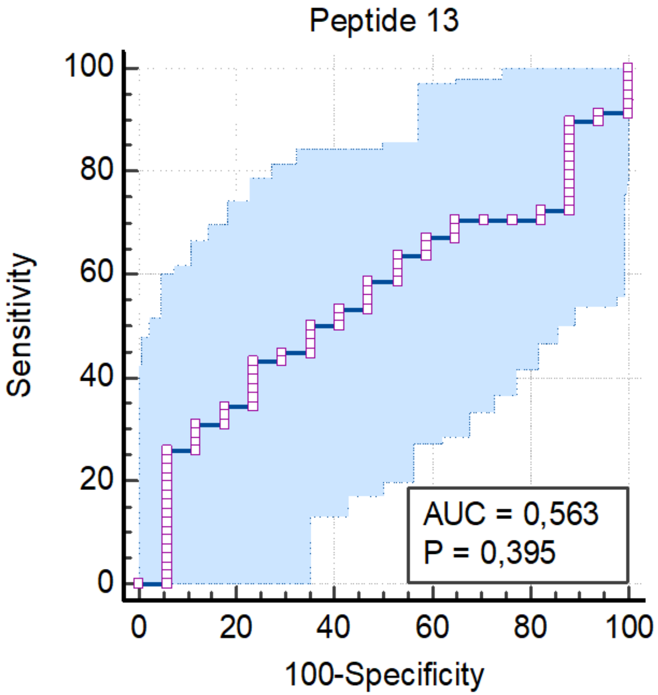

Criterion	Sensitivity	95% CI	Specificity	95% CI	+LR	95% CI	-LR	95% CI
≥-8759	100	93,8 - 100,0	0	0,0 - 19,5	1	1,00 - 1,00		
>-8759	98.28	90,8 - 100,0	0	0,0 - 19,5	0.98	0,95 - 1,02		
>-5886	96.55	88,1 - 99,6	0	0,0 - 19,5	0.97	0,92 - 1,01		
>-5835	94.83	85,6 - 98,9	0	0,0 - 19,5	0.95	0,89 - 1,01		
>-4695	93.1	83,3 - 98,1	0	0,0 - 19,5	0.93	0,87 - 1,00		
>-2536	91.38	81,0 - 97,1	0	0,0 - 19,5	0.91	0,84 - 0,99		
>-2415	91.38	81,0 - 97,1	5.88	0,1 - 28,7	0.97	0,84 - 1,12	1.47	0,18 - 11,71
>-2005	89.66	78,8 - 96,1	5.88	0,1 - 28,7	0.95	0,82 - 1,10	1.76	0,23 - 13,62
>-1958	89.66	78,8 - 96,1	11.76	1,5 - 36,4	1.02	0,84 - 1,23	0.88	0,19 - 3,97
>-1931	87.93	76,7 - 95,0	11.76	1,5 - 36,4	1	0,82 - 1,21	1.03	0,23 - 4,49
>-1771	86.21	74,6 - 93,9	11.76	1,5 - 36,4	0.98	0,80 - 1,20	1.17	0,27 - 5,01
>-1696	84.48	72,6 - 92,7	11.76	1,5 - 36,4	0.96	0,78 - 1,18	1.32	0,31 - 5,53
>-1680	82.76	70,6 - 91,4	11.76	1,5 - 36,4	0.94	0,76 - 1,16	1.47	0,35 - 6,06
>-1557	81.03	68,6 - 90,1	11.76	1,5 - 36,4	0.92	0,74 - 1,14	1.61	0,40 - 6,58
>-1472	79.31	66,6 - 88,8	11.76	1,5 - 36,4	0.9	0,72 - 1,12	1.76	0,44 - 7,10
>-1389	77.59	64,7 - 87,5	11.76	1,5 - 36,4	0.88	0,70 - 1,10	1.91	0,48 - 7,63
>-1329	75.86	62,8 - 86,1	11.76	1,5 - 36,4	0.86	0,69 - 1,08	2.05	0,52 - 8,15
>-1279	74.14	61,0 - 84,7	11.76	1,5 - 36,4	0.84	0,67 - 1,06	2.2	0,56 - 8,68
>-1267	72.41	59,1 - 83,3	11.76	1,5 - 36,4	0.82	0,65 - 1,04	2.34	0,60 - 9,20
>-1221	72.41	59,1 - 83,3	17.65	3,8 - 43,4	0.88	0,67 - 1,15	1.56	0,52 - 4,74
>-1208	70.69	57,3 - 81,9	17.65	3,8 - 43,4	0.86	0,65 - 1,13	1.66	0,55 - 5,00
>-1079	70.69	57,3 - 81,9	23.53	6,8 - 49,9	0.92	0,68 - 1,26	1.25	0,48 - 3,21
>-1059	70.69	57,3 - 81,9	29.41	10,3 - 56,0	1	0,71 - 1,42	1	0,43 - 2,30
>-1036	70.69	57,3 - 81,9	35.29	14,2 - 61,7	1.09	0,74 - 1,61	0.83	0,39 - 1,77

>-1031	68.97	55,5 - 80,5	35.29	14,2 - 61,7	1.07	0,72 - 1,58	0.88	0,42 - 1,86
>-1005	67.24	53,7 - 79,0	35.29	14,2 - 61,7	1.04	0,70 - 1,54	0.93	0,44 - 1,95
>-881	67.24	53,7 - 79,0	41.18	18,4 - 67,1	1.14	0,74 - 1,77	0.8	0,40 - 1,57
>-863	65.52	51,9 - 77,5	41.18	18,4 - 67,1	1.11	0,72 - 1,73	0.84	0,43 - 1,64
>-742	63.79	50,1 - 76,0	41.18	18,4 - 67,1	1.08	0,70 - 1,69	0.88	0,45 - 1,71
>-701	63.79	50,1 - 76,0	47.06	23,0 - 72,2	1.2	0,74 - 1,96	0.77	0,42 - 1,41
>-631	62.07	48,4 - 74,5	47.06	23,0 - 72,2	1.17	0,72 - 1,92	0.81	0,44 - 1,47
>-625	60.34	46,6 - 73,0	47.06	23,0 - 72,2	1.14	0,70 - 1,87	0.84	0,46 - 1,53
>-574	58.62	44,9 - 71,4	47.06	23,0 - 72,2	1.11	0,67 - 1,82	0.88	0,49 - 1,59
>-508	58.62	44,9 - 71,4	52.94	27,8 - 77,0	1.25	0,72 - 2,16	0.78	0,45 - 1,35
>-491	56.9	43,2 - 69,8	52.94	27,8 - 77,0	1.21	0,70 - 2,10	0.81	0,48 - 1,39
>-392	55.17	41,5 - 68,3	52.94	27,8 - 77,0	1.17	0,67 - 2,04	0.85	0,50 - 1,44
>-330	53.45	39,9 - 66,7	52.94	27,8 - 77,0	1.14	0,65 - 1,99	0.88	0,52 - 1,49
>-279	53.45	39,9 - 66,7	58.82	32,9 - 81,6	1.3	0,70 - 2,41	0.79	0,49 - 1,28
>-238	51.72	38,2 - 65,0	58.82	32,9 - 81,6	1.26	0,68 - 2,34	0.82	0,51 - 1,32
>-183	50	36,6 - 63,4	58.82	32,9 - 81,6	1.21	0,65 - 2,27	0.85	0,53 - 1,37
>30	50	36,6 - 63,4	64.71	38,3 - 85,8	1.42	0,71 - 2,83	0.77	0,50 - 1,19
>184	48.28	35,0 - 61,8	64.71	38,3 - 85,8	1.37	0,68 - 2,75	0.8	0,52 - 1,23
>262	46.55	33,3 - 60,1	64.71	38,3 - 85,8	1.32	0,65 - 2,66	0.83	0,54 - 1,26
>403	44.83	31,7 - 58,5	64.71	38,3 - 85,8	1.27	0,63 - 2,57	0.85	0,56 - 1,30
>406	44.83	31,7 - 58,5	70.59	44,0 - 89,7	1.52	0,69 - 3,36	0.78	0,53 - 1,15
>543	43.1	30,2 - 56,8	70.59	44,0 - 89,7	1.47	0,66 - 3,24	0.81	0,55 - 1,18
>556	43.1	30,2 - 56,8	76.47	50,1 - 93,2	1.83	0,74 - 4,54	0.74	0,53 - 1,05
>571	41.38	28,6 - 55,1	76.47	50,1 - 93,2	1.76	0,71 - 4,37	0.77	0,55 - 1,08
>794	39.66	27,0 - 53,4	76.47	50,1 - 93,2	1.69	0,68 - 4,20	0.79	0,56 - 1,10

>804	37.93	25,5 - 51,6	76.47	50,1 - 93,2	1.61	0,64 - 4,04	0.81	0,58 - 1,13
>874	36.21	24,0 - 49,9	76.47	50,1 - 93,2	1.54	0,61 - 3,87	0.83	0,60 - 1,16
>974	34.48	22,5 - 48,1	76.47	50,1 - 93,2	1.47	0,58 - 3,71	0.86	0,62 - 1,18
>1092	34.48	22,5 - 48,1	82.35	56,6 - 96,2	1.95	0,66 - 5,79	0.8	0,60 - 1,06
>1233	32.76	21,0 - 46,3	82.35	56,6 - 96,2	1.86	0,62 - 5,53	0.82	0,61 - 1,08
>1252	31.03	19,5 - 44,5	82.35	56,6 - 96,2	1.76	0,59 - 5,26	0.84	0,63 - 1,11
>1311	31.03	19,5 - 44,5	88.24	63,6 - 98,5	2.64	0,68 - 10,25	0.78	0,61 - 1,00
>1851	29.31	18,1 - 42,7	88.24	63,6 - 98,5	2.49	0,64 - 9,72	0.8	0,63 - 1,02
>2389	27.59	16,7 - 40,9	88.24	63,6 - 98,5	2.34	0,60 - 9,20	0.82	0,65 - 1,04
>2465	25.86	15,3 - 39,0	88.24	63,6 - 98,5	2.2	0,56 - 8,68	0.84	0,67 - 1,06
>2574	25.86	15,3 - 39,0	94.12	71,3 - 99,9	4.4	0,63 - 30,93	0.79	0,65 - 0,96
>2775	24.14	13,9 - 37,2	94.12	71,3 - 99,9	4.1	0,58 - 29,00	0.81	0,67 - 0,97
>3303	22.41	12,5 - 35,3	94.12	71,3 - 99,9	3.81	0,54 - 27,07	0.82	0,69 - 0,99
>3585	20.69	11,2 - 33,4	94.12	71,3 - 99,9	3.52	0,49 - 25,15	0.84	0,71 - 1,01
>4610	18.97	9,9 - 31,4	94.12	71,3 - 99,9	3.22	0,45 - 23,22	0.86	0,72 - 1,02
>4970	17.24	8,6 - 29,4	94.12	71,3 - 99,9	2.93	0,40 - 21,30	0.88	0,74 - 1,04
>5166	15.52	7,3 - 27,4	94.12	71,3 - 99,9	2.64	0,36 - 19,38	0.9	0,76 - 1,06
>5248	13.79	6,1 - 25,4	94.12	71,3 - 99,9	2.34	0,32 - 17,45	0.92	0,78 - 1,07
>5255	12.07	5,0 - 23,3	94.12	71,3 - 99,9	2.05	0,27 - 15,53	0.93	0,80 - 1,09
>7783	10.34	3,9 - 21,2	94.12	71,3 - 99,9	1.76	0,23 - 13,62	0.95	0,82 - 1,10
>12272	8.62	2,9 - 19,0	94.12	71,3 - 99,9	1.47	0,18 - 11,71	0.97	0,84 - 1,12
>15384	6.9	1,9 - 16,7	94.12	71,3 - 99,9	1.17	0,14 - 9,80	0.99	0,86 - 1,14
>17384	5.17	1,1 - 14,4	94.12	71,3 - 99,9	0.88	0,098 - 7,92	1.01	0,88 - 1,15
>18754	3.45	0,4 - 11,9	94.12	71,3 - 99,9	0.59	0,057 - 6,08	1.03	0,90 - 1,17
>18814	1.72	0,04 - 9,2	94.12	71,3 - 99,9	0.29	0,019 - 4,44	1.04	0,92 - 1,18
>19645	0	0,0 - 6,2	94.12	71,3 - 99,9	0		1.06	0,94 - 1,20
>21512	0	0,0 - 6,2	100	80,5 - 100,0			1	1,00 - 1,00

Monday, September 22, 2025 16:58 - MedCalc® version 23.3.7

6H

ROC curve

Variable	Peptid_16
Classification variable	status

Sample size	75
Positive group [a]	58 (77,33%)
Negative group [b]	17 (22,67%)

status = 1
[b] status = 0

Disease prevalence (%)	unknown

Area under the ROC curve (AUC)

Area under the ROC curve (AUC)	0.645
Standard Error [a]	0.0678
95% Confidence interval [b]	0,526 to 0,752
z statistic	2.13
Significance level P (Area=0.5)	0.0332

[a] DeLong et al., 1988
[b] Binomial exact

Youden index

Youden index J	0.3996
Associated criterion	≤-1823
Sensitivity	51.72
Specificity	88.24

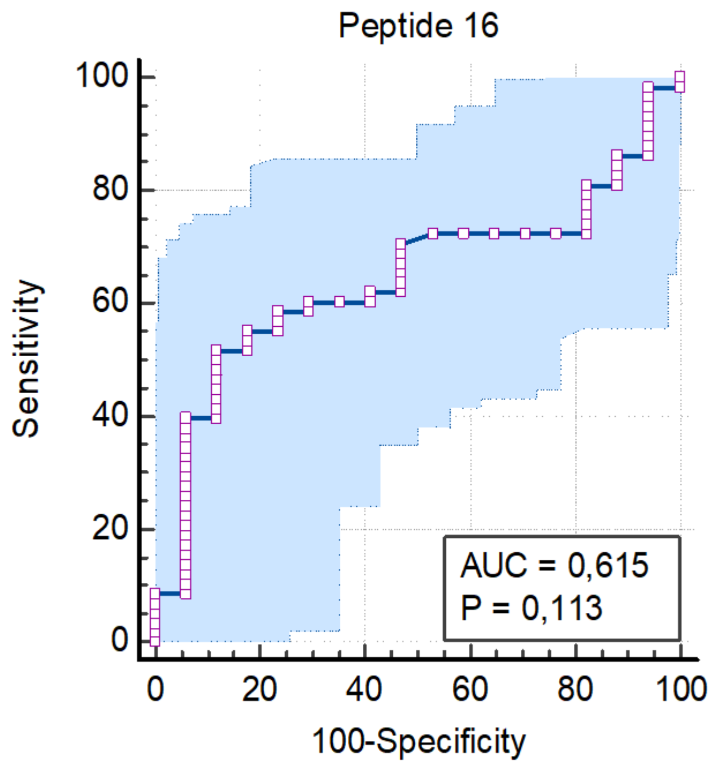

Criterion	Sensitivity	95% CI	Specificity	95% CI	+LR	95% CI	-LR	95% CI
<-14497	0	0,0 - 6,2	100	80,5 - 100,0			1	1,00 - 1,00
≤-14497	1.72	0,04 - 9,2	100	80,5 - 100,0			0.98	0,95 - 1,02
≤-7419	3.45	0,4 - 11,9	100	80,5 - 100,0			0.97	0,92 - 1,01
≤-6573	5.17	1,1 - 14,4	100	80,5 - 100,0			0.95	0,89 - 1,01
≤-6155	6.9	1,9 - 16,7	100	80,5 - 100,0			0.93	0,87 - 1,00
≤-5400	8.62	2,9 - 19,0	100	80,5 - 100,0			0.91	0,84 - 0,99
≤-5049	8.62	2,9 - 19,0	94.12	71,3 - 99,9	1.47	0,18 - 11,71	0.97	0,84 - 1,12
≤-5040	10.34	3,9 - 21,2	94.12	71,3 - 99,9	1.76	0,23 - 13,62	0.95	0,82 - 1,10
≤-4716	12.07	5,0 - 23,3	94.12	71,3 - 99,9	2.05	0,27 - 15,53	0.93	0,80 - 1,09
≤-4070	13.79	6,1 - 25,4	94.12	71,3 - 99,9	2.34	0,32 - 17,45	0.92	0,78 - 1,07
≤-3901	15.52	7,3 - 27,4	94.12	71,3 - 99,9	2.64	0,36 - 19,38	0.9	0,76 - 1,06
≤-3209	17.24	8,6 - 29,4	94.12	71,3 - 99,9	2.93	0,40 - 21,30	0.88	0,74 - 1,04
≤-2930	18.97	9,9 - 31,4	94.12	71,3 - 99,9	3.22	0,45 - 23,22	0.86	0,72 - 1,02
≤-2817	20.69	11,2 - 33,4	94.12	71,3 - 99,9	3.52	0,49 - 25,15	0.84	0,71 - 1,01
≤-2647	22.41	12,5 - 35,3	94.12	71,3 - 99,9	3.81	0,54 - 27,07	0.82	0,69 - 0,99
≤-2621	24.14	13,9 - 37,2	94.12	71,3 - 99,9	4.1	0,58 - 29,00	0.81	0,67 - 0,97
≤-2586	25.86	15,3 - 39,0	94.12	71,3 - 99,9	4.4	0,63 - 30,93	0.79	0,65 - 0,96
≤-2476	27.59	16,7 - 40,9	94.12	71,3 - 99,9	4.69	0,67 - 32,85	0.77	0,63 - 0,94
≤-2444	29.31	18,1 - 42,7	94.12	71,3 - 99,9	4.98	0,71 - 34,78	0.75	0,61 - 0,92
≤-2379	31.03	19,5 - 44,5	94.12	71,3 - 99,9	5.28	0,76 - 36,71	0.73	0,59 - 0,90
≤-2319	32.76	21,0 - 46,3	94.12	71,3 - 99,9	5.57	0,80 - 38,63	0.71	0,58 - 0,89
≤-2270	34.48	22,5 - 48,1	94.12	71,3 - 99,9	5.86	0,85 - 40,56	0.7	0,56 - 0,87
≤-2197	36.21	24,0 - 49,9	94.12	71,3 - 99,9	6.16	0,89 - 42,49	0.68	0,54 - 0,85
≤-2165	37.93	25,5 - 51,6	94.12	71,3 - 99,9	6.45	0,94 - 44,42	0.66	0,52 - 0,83

≤-2158	39.66	27,0 - 53,4	94.12	71,3 - 99,9	6.74	0,98 - 46,34	0.64	0,50 - 0,82
≤-2114	39.66	27,0 - 53,4	88.24	63,6 - 98,5	3.37	0,88 - 12,87	0.68	0,52 - 0,90
≤-2085	41.38	28,6 - 55,1	88.24	63,6 - 98,5	3.52	0,92 - 13,40	0.66	0,50 - 0,88
≤-2000	43.1	30,2 - 56,8	88.24	63,6 - 98,5	3.66	0,96 - 13,92	0.64	0,49 - 0,86
≤-1932	44.83	31,7 - 58,5	88.24	63,6 - 98,5	3.81	1,00 - 14,45	0.63	0,47 - 0,84
≤-1879	46.55	33,3 - 60,1	88.24	63,6 - 98,5	3.96	1,05 - 14,97	0.61	0,45 - 0,81
≤-1862	48.28	35,0 - 61,8	88.24	63,6 - 98,5	4.1	1,09 - 15,50	0.59	0,43 - 0,79
≤-1855	50	36,6 - 63,4	88.24	63,6 - 98,5	4.25	1,13 - 16,02	0.57	0,42 - 0,77
≤-1823	51.72	38,2 - 65,0	88.24	63,6 - 98,5	4.4	1,17 - 16,55	0.55	0,40 - 0,75
≤-1770	51.72	38,2 - 65,0	82.35	56,6 - 96,2	2.93	1,02 - 8,43	0.59	0,41 - 0,83
≤-1709	53.45	39,9 - 66,7	82.35	56,6 - 96,2	3.03	1,05 - 8,70	0.57	0,40 - 0,80
≤-1704	55.17	41,5 - 68,3	82.35	56,6 - 96,2	3.13	1,09 - 8,96	0.54	0,38 - 0,78
≤-1691	55.17	41,5 - 68,3	76.47	50,1 - 93,2	2.34	0,97 - 5,70	0.59	0,40 - 0,86
≤-1614	56.9	43,2 - 69,8	76.47	50,1 - 93,2	2.42	1,00 - 5,86	0.56	0,38 - 0,84
≤-1588	58.62	44,9 - 71,4	76.47	50,1 - 93,2	2.49	1,03 - 6,03	0.54	0,36 - 0,81
≤-1568	58.62	44,9 - 71,4	70.59	44,0 - 89,7	1.99	0,93 - 4,29	0.59	0,38 - 0,90
≤-1555	60.34	46,6 - 73,0	70.59	44,0 - 89,7	2.05	0,95 - 4,41	0.56	0,36 - 0,87
≤-1521	60.34	46,6 - 73,0	64.71	38,3 - 85,8	1.71	0,87 - 3,36	0.61	0,38 - 0,98
≤-1508	60.34	46,6 - 73,0	58.82	32,9 - 81,6	1.47	0,80 - 2,68	0.67	0,41 - 1,12
≤-1496	62.07	48,4 - 74,5	58.82	32,9 - 81,6	1.51	0,83 - 2,75	0.64	0,38 - 1,08
≤-1449	62.07	48,4 - 74,5	52.94	27,8 - 77,0	1.32	0,77 - 2,27	0.72	0,41 - 1,25
≤-1439	63.79	50,1 - 76,0	52.94	27,8 - 77,0	1.36	0,79 - 2,33	0.68	0,39 - 1,20
≤-1433	65.52	51,9 - 77,5	52.94	27,8 - 77,0	1.39	0,81 - 2,38	0.65	0,37 - 1,15
≤-1411	67.24	53,7 - 79,0	52.94	27,8 - 77,0	1.43	0,84 - 2,44	0.62	0,35 - 1,11
≤-1382	68.97	55,5 - 80,5	52.94	27,8 - 77,0	1.47	0,86 - 2,50	0.59	0,32 - 1,06

≤-1348	70.69	57,3 - 81,9	52.94	27,8 - 77,0	1.5	0,88 - 2,55	0.55	0,30 - 1,01
≤-1329	72.41	59,1 - 83,3	47.06	23,0 - 72,2	1.37	0,85 - 2,20	0.59	0,30 - 1,13
≤-1300	72.41	59,1 - 83,3	41.18	18,4 - 67,1	1.23	0,80 - 1,89	0.67	0,33 - 1,36
≤-1289	72.41	59,1 - 83,3	35.29	14,2 - 61,7	1.12	0,76 - 1,65	0.78	0,36 - 1,68
≤-1247	72.41	59,1 - 83,3	29.41	10,3 - 56,0	1.03	0,73 - 1,45	0.94	0,40 - 2,19
≤-1239	72.41	59,1 - 83,3	23.53	6,8 - 49,9	0.95	0,70 - 1,29	1.17	0,45 - 3,04
≤-1234	72.41	59,1 - 83,3	17.65	3,8 - 43,4	0.88	0,67 - 1,15	1.56	0,52 - 4,74
≤-1229	74.14	61,0 - 84,7	17.65	3,8 - 43,4	0.9	0,69 - 1,18	1.47	0,48 - 4,47
≤-1225	75.86	62,8 - 86,1	17.65	3,8 - 43,4	0.92	0,71 - 1,20	1.37	0,44 - 4,21
≤-1093	77.59	64,7 - 87,5	17.65	3,8 - 43,4	0.94	0,73 - 1,22	1.27	0,41 - 3,94
≤-1058	79.31	66,6 - 88,8	17.65	3,8 - 43,4	0.96	0,75 - 1,24	1.17	0,37 - 3,68
≤-1055	81.03	68,6 - 90,1	17.65	3,8 - 43,4	0.98	0,76 - 1,27	1.07	0,34 - 3,42
≤-1019	81.03	68,6 - 90,1	11.76	1,5 - 36,4	0.92	0,74 - 1,14	1.61	0,40 - 6,58
≤-982	82.76	70,6 - 91,4	11.76	1,5 - 36,4	0.94	0,76 - 1,16	1.47	0,35 - 6,06
≤-967	84.48	72,6 - 92,7	11.76	1,5 - 36,4	0.96	0,78 - 1,18	1.32	0,31 - 5,53
≤-955	86.21	74,6 - 93,9	11.76	1,5 - 36,4	0.98	0,80 - 1,20	1.17	0,27 - 5,01
≤-951	86.21	74,6 - 93,9	5.88	0,1 - 28,7	0.92	0,78 - 1,07	2.34	0,32 - 17,45
≤-821	87.93	76,7 - 95,0	5.88	0,1 - 28,7	0.93	0,80 - 1,09	2.05	0,27 - 15,53
≤-767	89.66	78,8 - 96,1	5.88	0,1 - 28,7	0.95	0,82 - 1,10	1.76	0,23 - 13,62
≤-614	91.38	81,0 - 97,1	5.88	0,1 - 28,7	0.97	0,84 - 1,12	1.47	0,18 - 11,71
≤-418	93.1	83,3 - 98,1	5.88	0,1 - 28,7	0.99	0,86 - 1,14	1.17	0,14 - 9,80
≤74	94.83	85,6 - 98,9	5.88	0,1 - 28,7	1.01	0,88 - 1,15	0.88	0,098 - 7,92
≤663	96.55	88,1 - 99,6	5.88	0,1 - 28,7	1.03	0,90 - 1,17	0.59	0,057 - 6,08
≤2606	98.28	90,8 - 100,0	5.88	0,1 - 28,7	1.04	0,92 - 1,18	0.29	0,019 - 4,44
≤2725	98.28	90,8 - 100,0	0	0,0 - 19,5	0.98	0,95 - 1,02		
≤17136	100	93,8 - 100,0	0	0,0 - 19,5	1	1,00 - 1,00		

Monday, September 22, 2025 16:58 - MedCalc® version 23.3.7

6I

ROC curve

Variable	Peptid_17
Classification variable	status

Sample size	75
Positive group [a]	58 (77,33%)
Negative group [b]	17 (22,67%)

status = 1
[b] status = 0

Disease prevalence (%)	unknown

Area under the ROC curve (AUC)

Area under the ROC curve (AUC)	0.529
Standard Error [a]	0.0706
95% Confidence interval [b]	0,411 to 0,646
z statistic	0.416
Significance level P (Area=0.5)	0.6772

[a] DeLong et al., 1988
[b] Binomial exact

Youden index

Youden index J	0.2343
Associated criterion	>-1982
Sensitivity	70.69
Specificity	5.88

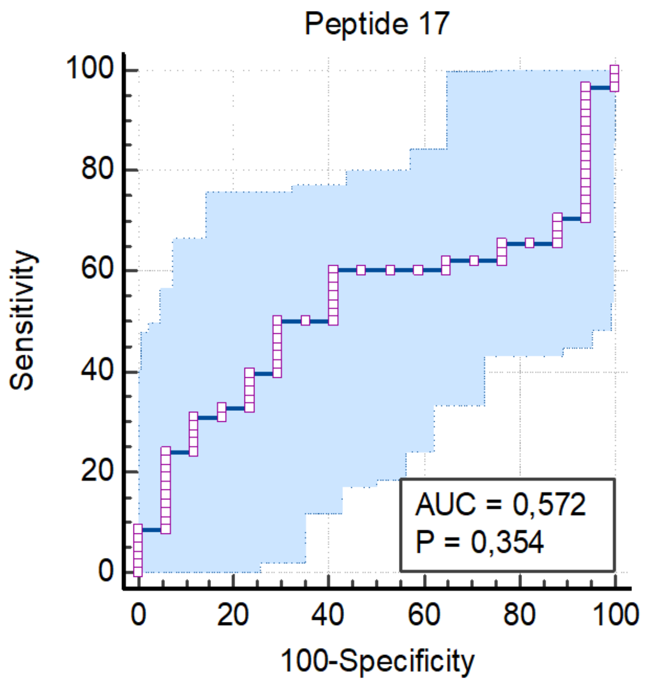

Criterion	Sensitivity	95% CI	Specificity	95% CI	+LR	95% CI	-LR	95% CI
≥-13658	100	93,8 - 100,0	0	0,0 - 19,5	1	1,00 - 1,00		
>-13658	98.28	90,8 - 100,0	0	0,0 - 19,5	0.98	0,95 - 1,02		
>-7795	96.55	88,1 - 99,6	0	0,0 - 19,5	0.97	0,92 - 1,01		
>-6614	96.55	88,1 - 99,6	5.88	0,1 - 28,7	1.03	0,90 - 1,17	0.59	0,057 - 6,08
>-4648	94.83	85,6 - 98,9	5.88	0,1 - 28,7	1.01	0,88 - 1,15	0.88	0,098 - 7,92
>-3426	93.1	83,3 - 98,1	5.88	0,1 - 28,7	0.99	0,86 - 1,14	1.17	0,14 - 9,80
>-3202	91.38	81,0 - 97,1	5.88	0,1 - 28,7	0.97	0,84 - 1,12	1.47	0,18 - 11,71
>-2819	89.66	78,8 - 96,1	5.88	0,1 - 28,7	0.95	0,82 - 1,10	1.76	0,23 - 13,62
>-2681	87.93	76,7 - 95,0	5.88	0,1 - 28,7	0.93	0,80 - 1,09	2.05	0,27 - 15,53
>-2564	86.21	74,6 - 93,9	5.88	0,1 - 28,7	0.92	0,78 - 1,07	2.34	0,32 - 17,45
>-2428	84.48	72,6 - 92,7	5.88	0,1 - 28,7	0.9	0,76 - 1,06	2.64	0,36 - 19,38
>-2373	82.76	70,6 - 91,4	5.88	0,1 - 28,7	0.88	0,74 - 1,04	2.93	0,40 - 21,30
>-2359	81.03	68,6 - 90,1	5.88	0,1 - 28,7	0.86	0,72 - 1,02	3.22	0,45 - 23,22
>-2224	79.31	66,6 - 88,8	5.88	0,1 - 28,7	0.84	0,71 - 1,01	3.52	0,49 - 25,15
>-2217	77.59	64,7 - 87,5	5.88	0,1 - 28,7	0.82	0,69 - 0,99	3.81	0,54 - 27,07
>-2122	75.86	62,8 - 86,1	5.88	0,1 - 28,7	0.81	0,67 - 0,97	4.1	0,58 - 29,00
>-2112	74.14	61,0 - 84,7	5.88	0,1 - 28,7	0.79	0,65 - 0,96	4.4	0,63 - 30,93
>-2026	72.41	59,1 - 83,3	5.88	0,1 - 28,7	0.77	0,63 - 0,94	4.69	0,67 - 32,85
>-1982	70.69	57,3 - 81,9	5.88	0,1 - 28,7	0.75	0,61 - 0,92	4.98	0,71 - 34,78
>-1928	70.69	57,3 - 81,9	11.76	1,5 - 36,4	0.8	0,63 - 1,02	2.49	0,64 - 9,72
>-1874	68.97	55,5 - 80,5	11.76	1,5 - 36,4	0.78	0,61 - 1,00	2.64	0,68 - 10,25
>-1813	67.24	53,7 - 79,0	11.76	1,5 - 36,4	0.76	0,59 - 0,98	2.78	0,72 - 10,77

>-1674	65.52	51,9 - 77,5	11.76	1,5 - 36,4	0.74	0,58 - 0,96	2.93	0,76 - 11,30
>-1594	65.52	51,9 - 77,5	17.65	3,8 - 43,4	0.8	0,60 - 1,06	1.95	0,66 - 5,79
>-1501	65.52	51,9 - 77,5	23.53	6,8 - 49,9	0.86	0,62 - 1,18	1.47	0,58 - 3,71
>-1472	63.79	50,1 - 76,0	23.53	6,8 - 49,9	0.83	0,60 - 1,16	1.54	0,61 - 3,87
>-1452	62.07	48,4 - 74,5	23.53	6,8 - 49,9	0.81	0,58 - 1,13	1.61	0,64 - 4,04
>-1351	62.07	48,4 - 74,5	29.41	10,3 - 56,0	0.88	0,61 - 1,27	1.29	0,58 - 2,89
>-1323	62.07	48,4 - 74,5	35.29	14,2 - 61,7	0.96	0,64 - 1,44	1.07	0,52 - 2,21
>-1307	60.34	46,6 - 73,0	35.29	14,2 - 61,7	0.93	0,62 - 1,40	1.12	0,55 - 2,30
>-1267	60.34	46,6 - 73,0	41.18	18,4 - 67,1	1.03	0,65 - 1,61	0.96	0,50 - 1,85
>-1217	60.34	46,6 - 73,0	47.06	23,0 - 72,2	1.14	0,70 - 1,87	0.84	0,46 - 1,53
>-1083	60.34	46,6 - 73,0	52.94	27,8 - 77,0	1.28	0,74 - 2,21	0.75	0,43 - 1,30
>-974	60.34	46,6 - 73,0	58.82	32,9 - 81,6	1.47	0,80 - 2,68	0.67	0,41 - 1,12
>-935	58.62	44,9 - 71,4	58.82	32,9 - 81,6	1.42	0,78 - 2,61	0.7	0,43 - 1,16
>-891	56.9	43,2 - 69,8	58.82	32,9 - 81,6	1.38	0,75 - 2,54	0.73	0,45 - 1,20
>-880	55.17	41,5 - 68,3	58.82	32,9 - 81,6	1.34	0,73 - 2,48	0.76	0,47 - 1,24
>-862	53.45	39,9 - 66,7	58.82	32,9 - 81,6	1.3	0,70 - 2,41	0.79	0,49 - 1,28
>-840	51.72	38,2 - 65,0	58.82	32,9 - 81,6	1.26	0,68 - 2,34	0.82	0,51 - 1,32
>-816	50	36,6 - 63,4	58.82	32,9 - 81,6	1.21	0,65 - 2,27	0.85	0,53 - 1,37
>-760	50	36,6 - 63,4	64.71	38,3 - 85,8	1.42	0,71 - 2,83	0.77	0,50 - 1,19
>-520	50	36,6 - 63,4	70.59	44,0 - 89,7	1.7	0,78 - 3,71	0.71	0,47 - 1,06
>-409	48.28	35,0 - 61,8	70.59	44,0 - 89,7	1.64	0,75 - 3,59	0.73	0,49 - 1,09
>-209	46.55	33,3 - 60,1	70.59	44,0 - 89,7	1.58	0,72 - 3,47	0.76	0,51 - 1,12
>2	44.83	31,7 - 58,5	70.59	44,0 - 89,7	1.52	0,69 - 3,36	0.78	0,53 - 1,15
>155	43.1	30,2 - 56,8	70.59	44,0 - 89,7	1.47	0,66 - 3,24	0.81	0,55 - 1,18
>306	41.38	28,6 - 55,1	70.59	44,0 - 89,7	1.41	0,63 - 3,12	0.83	0,57 - 1,21

>311	39.66	27,0 - 53,4	70.59	44,0 - 89,7	1.35	0,60 - 3,01	0.85	0,59 - 1,24
>338	39.66	27,0 - 53,4	76.47	50,1 - 93,2	1.69	0,68 - 4,20	0.79	0,56 - 1,10
>413	37.93	25,5 - 51,6	76.47	50,1 - 93,2	1.61	0,64 - 4,04	0.81	0,58 - 1,13
>840	36.21	24,0 - 49,9	76.47	50,1 - 93,2	1.54	0,61 - 3,87	0.83	0,60 - 1,16
>935	34.48	22,5 - 48,1	76.47	50,1 - 93,2	1.47	0,58 - 3,71	0.86	0,62 - 1,18
>1675	32.76	21,0 - 46,3	76.47	50,1 - 93,2	1.39	0,55 - 3,54	0.88	0,64 - 1,21
>1982	32.76	21,0 - 46,3	82.35	56,6 - 96,2	1.86	0,62 - 5,53	0.82	0,61 - 1,08
>2008	31.03	19,5 - 44,5	82.35	56,6 - 96,2	1.76	0,59 - 5,26	0.84	0,63 - 1,11
>2125	31.03	19,5 - 44,5	88.24	63,6 - 98,5	2.64	0,68 - 10,25	0.78	0,61 - 1,00
>2201	29.31	18,1 - 42,7	88.24	63,6 - 98,5	2.49	0,64 - 9,72	0.8	0,63 - 1,02
>2243	27.59	16,7 - 40,9	88.24	63,6 - 98,5	2.34	0,60 - 9,20	0.82	0,65 - 1,04
>2453	25.86	15,3 - 39,0	88.24	63,6 - 98,5	2.2	0,56 - 8,68	0.84	0,67 - 1,06
>2571	24.14	13,9 - 37,2	88.24	63,6 - 98,5	2.05	0,52 - 8,15	0.86	0,69 - 1,08
>2680	24.14	13,9 - 37,2	94.12	71,3 - 99,9	4.1	0,58 - 29,00	0.81	0,67 - 0,97
>2725	22.41	12,5 - 35,3	94.12	71,3 - 99,9	3.81	0,54 - 27,07	0.82	0,69 - 0,99
>2871	20.69	11,2 - 33,4	94.12	71,3 - 99,9	3.52	0,49 - 25,15	0.84	0,71 - 1,01
>2911	18.97	9,9 - 31,4	94.12	71,3 - 99,9	3.22	0,45 - 23,22	0.86	0,72 - 1,02
>3055	17.24	8,6 - 29,4	94.12	71,3 - 99,9	2.93	0,40 - 21,30	0.88	0,74 - 1,04
>3066	15.52	7,3 - 27,4	94.12	71,3 - 99,9	2.64	0,36 - 19,38	0.9	0,76 - 1,06
>3163	13.79	6,1 - 25,4	94.12	71,3 - 99,9	2.34	0,32 - 17,45	0.92	0,78 - 1,07
>3441	12.07	5,0 - 23,3	94.12	71,3 - 99,9	2.05	0,27 - 15,53	0.93	0,80 - 1,09
>3602	10.34	3,9 - 21,2	94.12	71,3 - 99,9	1.76	0,23 - 13,62	0.95	0,82 - 1,10
>3613	8.62	2,9 - 19,0	94.12	71,3 - 99,9	1.47	0,18 - 11,71	0.97	0,84 - 1,12
>4373	8.62	2,9 - 19,0	100	80,5 - 100,0			0.91	0,84 - 0,99
>6267	6.9	1,9 - 16,7	100	80,5 - 100,0			0.93	0,87 - 1,00
>7320	5.17	1,1 - 14,4	100	80,5 - 100,0			0.95	0,89 - 1,01
>8762	3.45	0,4 - 11,9	100	80,5 - 100,0			0.97	0,92 - 1,01
>9079	1.72	0,04 - 9,2	100	80,5 - 100,0			0.98	0,95 - 1,02
>9547	0	0,0 - 6,2	100	80,5 - 100,0			1	1,00 - 1,00

Monday, September 22, 2025 16:59 - MedCalc® version 23.3.7

6J

ROC curve

Variable	Peptid_19
Classification variable	status

Sample size	75
Positive group [a]	58 (77,33%)
Negative group [b]	17 (22,67%)

status = 1
[b] status = 0

Disease prevalence (%)	unknown

Area under the ROC curve (AUC)

Area under the ROC curve (AUC)	0.555
Standard Error [a]	0.0669
95% Confidence interval [b]	0,436 to 0,670
z statistic	0.826
Significance level P (Area=0.5)	0.4086

[a] DeLong et al., 1988
[b] Binomial exact

Youden index

Youden index J	0.3793
Associated criterion	>-1099
Sensitivity	37.93
Specificity	100

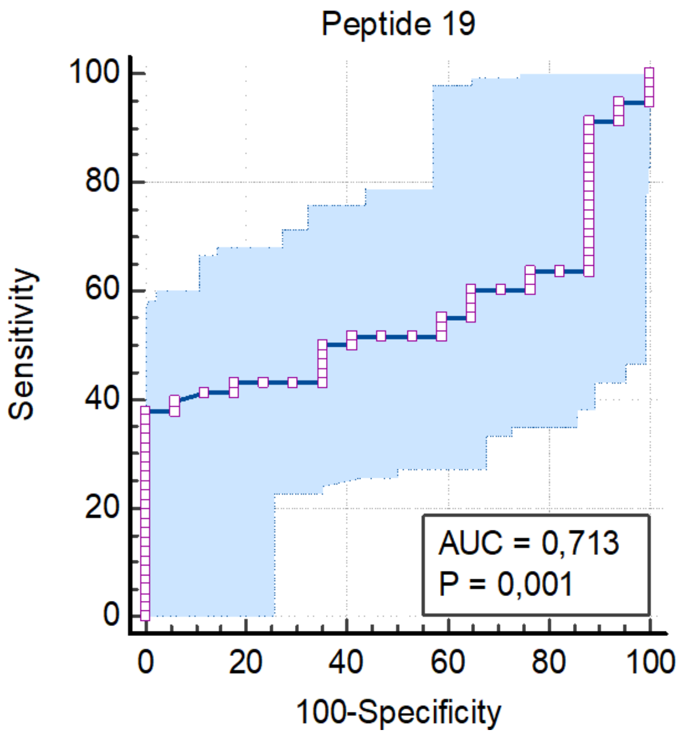

Criterion	Sensitivity	95% CI	Specificity	95% CI	+LR	95% CI	-LR	95% CI
≥-10555	100	93,8 - 100,0	0	0,0 - 19,5	1	1,00 - 1,00		
>-10555	98.28	90,8 - 100,0	0	0,0 - 19,5	0.98	0,95 - 1,02		
>-9455	96.55	88,1 - 99,6	0	0,0 - 19,5	0.97	0,92 - 1,01		
>-8695	94.83	85,6 - 98,9	0	0,0 - 19,5	0.95	0,89 - 1,01		
>-8193	94.83	85,6 - 98,9	5.88	0,1 - 28,7	1.01	0,88 - 1,15	0.88	0,098 - 7,92
>-6509	93.1	83,3 - 98,1	5.88	0,1 - 28,7	0.99	0,86 - 1,14	1.17	0,14 - 9,80
>-5342	91.38	81,0 - 97,1	5.88	0,1 - 28,7	0.97	0,84 - 1,12	1.47	0,18 - 11,71
>-3867	91.38	81,0 - 97,1	11.76	1,5 - 36,4	1.04	0,86 - 1,25	0.73	0,16 - 3,45
>-3501	89.66	78,8 - 96,1	11.76	1,5 - 36,4	1.02	0,84 - 1,23	0.88	0,19 - 3,97
>-3278	87.93	76,7 - 95,0	11.76	1,5 - 36,4	1	0,82 - 1,21	1.03	0,23 - 4,49
>-3047	86.21	74,6 - 93,9	11.76	1,5 - 36,4	0.98	0,80 - 1,20	1.17	0,27 - 5,01
>-2972	84.48	72,6 - 92,7	11.76	1,5 - 36,4	0.96	0,78 - 1,18	1.32	0,31 - 5,53
>-2957	82.76	70,6 - 91,4	11.76	1,5 - 36,4	0.94	0,76 - 1,16	1.47	0,35 - 6,06
>-2940	81.03	68,6 - 90,1	11.76	1,5 - 36,4	0.92	0,74 - 1,14	1.61	0,40 - 6,58
>-2925	79.31	66,6 - 88,8	11.76	1,5 - 36,4	0.9	0,72 - 1,12	1.76	0,44 - 7,10
>-2906	77.59	64,7 - 87,5	11.76	1,5 - 36,4	0.88	0,70 - 1,10	1.91	0,48 - 7,63
>-2676	75.86	62,8 - 86,1	11.76	1,5 - 36,4	0.86	0,69 - 1,08	2.05	0,52 - 8,15
>-2528	74.14	61,0 - 84,7	11.76	1,5 - 36,4	0.84	0,67 - 1,06	2.2	0,56 - 8,68
>-2522	72.41	59,1 - 83,3	11.76	1,5 - 36,4	0.82	0,65 - 1,04	2.34	0,60 - 9,20
>-2459	70.69	57,3 - 81,9	11.76	1,5 - 36,4	0.8	0,63 - 1,02	2.49	0,64 - 9,72
>-2342	68.97	55,5 - 80,5	11.76	1,5 - 36,4	0.78	0,61 - 1,00	2.64	0,68 - 10,25
>-2236	67.24	53,7 - 79,0	11.76	1,5 - 36,4	0.76	0,59 - 0,98	2.78	0,72 - 10,77
>-2224	65.52	51,9 - 77,5	11.76	1,5 - 36,4	0.74	0,58 - 0,96	2.93	0,76 - 11,30
>-2214	63.79	50,1 - 76,0	11.76	1,5 - 36,4	0.72	0,56 - 0,94	3.08	0,80 - 11,82

>-2191	63.79	50,1 - 76,0	17.65	3,8 - 43,4	0.77	0,58 - 1,04	2.05	0,70 - 6,06
>-2087	63.79	50,1 - 76,0	23.53	6,8 - 49,9	0.83	0,60 - 1,16	1.54	0,61 - 3,87
>-1976	62.07	48,4 - 74,5	23.53	6,8 - 49,9	0.81	0,58 - 1,13	1.61	0,64 - 4,04
>-1912	60.34	46,6 - 73,0	23.53	6,8 - 49,9	0.79	0,56 - 1,10	1.69	0,68 - 4,20
>-1877	60.34	46,6 - 73,0	29.41	10,3 - 56,0	0.85	0,59 - 1,24	1.35	0,60 - 3,01
>-1875	60.34	46,6 - 73,0	35.29	14,2 - 61,7	0.93	0,62 - 1,40	1.12	0,55 - 2,30
>-1835	58.62	44,9 - 71,4	35.29	14,2 - 61,7	0.91	0,60 - 1,37	1.17	0,57 - 2,39
>-1826	56.9	43,2 - 69,8	35.29	14,2 - 61,7	0.88	0,58 - 1,33	1.22	0,60 - 2,48
>-1778	55.17	41,5 - 68,3	35.29	14,2 - 61,7	0.85	0,56 - 1,30	1.27	0,63 - 2,57
>-1759	55.17	41,5 - 68,3	41.18	18,4 - 67,1	0.94	0,59 - 1,49	1.09	0,58 - 2,06
>-1682	53.45	39,9 - 66,7	41.18	18,4 - 67,1	0.91	0,57 - 1,45	1.13	0,60 - 2,13
>-1633	51.72	38,2 - 65,0	41.18	18,4 - 67,1	0.88	0,55 - 1,41	1.17	0,63 - 2,20
>-1613	51.72	38,2 - 65,0	47.06	23,0 - 72,2	0.98	0,59 - 1,63	1.03	0,58 - 1,81
>-1588	51.72	38,2 - 65,0	52.94	27,8 - 77,0	1.1	0,63 - 1,93	0.91	0,54 - 1,54
>-1573	51.72	38,2 - 65,0	58.82	32,9 - 81,6	1.26	0,68 - 2,34	0.82	0,51 - 1,32
>-1571	50	36,6 - 63,4	58.82	32,9 - 81,6	1.21	0,65 - 2,27	0.85	0,53 - 1,37
>-1556	50	36,6 - 63,4	64.71	38,3 - 85,8	1.42	0,71 - 2,83	0.77	0,50 - 1,19
>-1521	48.28	35,0 - 61,8	64.71	38,3 - 85,8	1.37	0,68 - 2,75	0.8	0,52 - 1,23
>-1490	46.55	33,3 - 60,1	64.71	38,3 - 85,8	1.32	0,65 - 2,66	0.83	0,54 - 1,26
>-1460	44.83	31,7 - 58,5	64.71	38,3 - 85,8	1.27	0,63 - 2,57	0.85	0,56 - 1,30
>-1452	43.1	30,2 - 56,8	64.71	38,3 - 85,8	1.22	0,60 - 2,48	0.88	0,58 - 1,33
>-1424	43.1	30,2 - 56,8	70.59	44,0 - 89,7	1.47	0,66 - 3,24	0.81	0,55 - 1,18
>-1386	43.1	30,2 - 56,8	76.47	50,1 - 93,2	1.83	0,74 - 4,54	0.74	0,53 - 1,05
>-1337	43.1	30,2 - 56,8	82.35	56,6 - 96,2	2.44	0,84 - 7,11	0.69	0,50 - 0,95
>-1327	41.38	28,6 - 55,1	82.35	56,6 - 96,2	2.34	0,80 - 6,85	0.71	0,52 - 0,97

>-1314	41.38	28,6 - 55,1	88.24	63,6 - 98,5	3.52	0,92 - 13,40	0.66	0,50 - 0,88
>-1282	39.66	27,0 - 53,4	94.12	71,3 - 99,9	6.74	0,98 - 46,34	0.64	0,50 - 0,82
>-1122	37.93	25,5 - 51,6	94.12	71,3 - 99,9	6.45	0,94 - 44,42	0.66	0,52 - 0,83
>-1099	37.93	25,5 - 51,6	100	80,5 - 100,0			0.62	0,51 - 0,76
>-967	36.21	24,0 - 49,9	100	80,5 - 100,0			0.64	0,53 - 0,77
>-906	34.48	22,5 - 48,1	100	80,5 - 100,0			0.66	0,54 - 0,79
>-853	32.76	21,0 - 46,3	100	80,5 - 100,0			0.67	0,56 - 0,80
>-783	31.03	19,5 - 44,5	100	80,5 - 100,0			0.69	0,58 - 0,82
>-732	29.31	18,1 - 42,7	100	80,5 - 100,0			0.71	0,60 - 0,83
>-654	27.59	16,7 - 40,9	100	80,5 - 100,0			0.72	0,62 - 0,85
>-289	25.86	15,3 - 39,0	100	80,5 - 100,0			0.74	0,64 - 0,86
>-249	24.14	13,9 - 37,2	100	80,5 - 100,0			0.76	0,66 - 0,88
>-242	22.41	12,5 - 35,3	100	80,5 - 100,0			0.78	0,68 - 0,89
>-180	20.69	11,2 - 33,4	100	80,5 - 100,0			0.79	0,70 - 0,90
>-58	18.97	9,9 - 31,4	100	80,5 - 100,0			0.81	0,72 - 0,92
>23	17.24	8,6 - 29,4	100	80,5 - 100,0			0.83	0,74 - 0,93
>173	15.52	7,3 - 27,4	100	80,5 - 100,0			0.84	0,76 - 0,94
>385	13.79	6,1 - 25,4	100	80,5 - 100,0			0.86	0,78 - 0,96
>837	12.07	5,0 - 23,3	100	80,5 - 100,0			0.88	0,80 - 0,97
>1017	10.34	3,9 - 21,2	100	80,5 - 100,0			0.9	0,82 - 0,98
>1316	8.62	2,9 - 19,0	100	80,5 - 100,0			0.91	0,84 - 0,99
>1380	6.9	1,9 - 16,7	100	80,5 - 100,0			0.93	0,87 - 1,00
>1837	5.17	1,1 - 14,4	100	80,5 - 100,0			0.95	0,89 - 1,01
>2044	3.45	0,4 - 11,9	100	80,5 - 100,0			0.97	0,92 - 1,01
>2924	1.72	0,04 - 9,2	100	80,5 - 100,0			0.98	0,95 - 1,02
>3510	0	0,0 - 6,2	100	80,5 - 100,0			1	1,00 - 1,00

Monday, September 22, 2025 17:00 - MedCalc® version 23.3.7

6K

ROC curve

Variable	Peptid_20
Classification variable	status

Sample size	75
Positive group [a]	58 (77,33%)
Negative group [b]	17 (22,67%)

status = 1
[b] status = 0

Disease prevalence (%)	unknown

Area under the ROC curve (AUC)

Area under the ROC curve (AUC)	0.52
Standard Error [a]	0.0663
95% Confidence interval [b]	0,401 to 0,637
z statistic	0.298
Significance level P (Area=0.5)	0.7656

[a] DeLong et al., 1988
[b] Binomial exact

Youden index

Youden index J	0.3966
Associated criterion	>-964
Sensitivity	39.66
Specificity	100

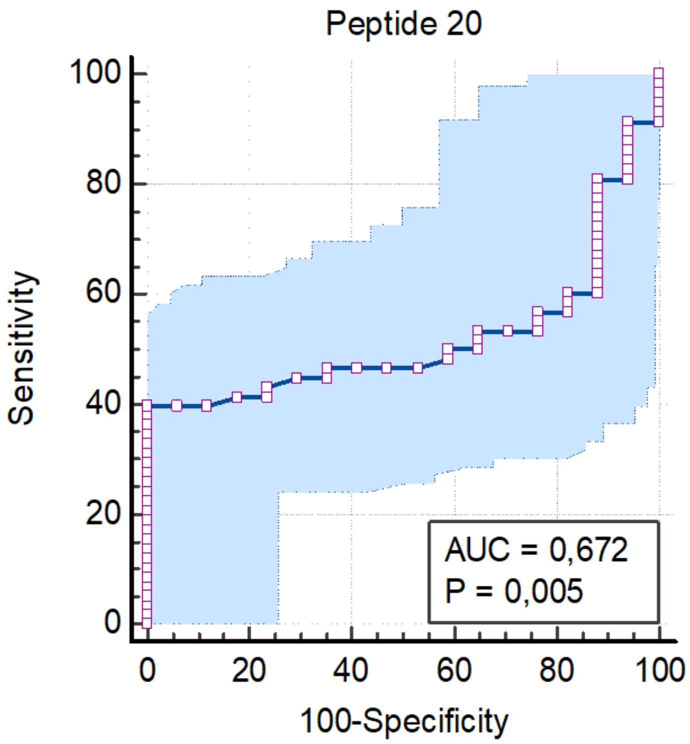

Criterion	Sensitivity	95% CI	Specificity	95% CI	+LR	95% CI	-LR	95% CI
≥-10537	100	93,8 - 100,0	0	0,0 - 19,5	1	1,00 - 1,00		
>-10537	98.28	90,8 - 100,0	0	0,0 - 19,5	0.98	0,95 - 1,02		
>-5477	96.55	88,1 - 99,6	0	0,0 - 19,5	0.97	0,92 - 1,01		
>-4726	94.83	85,6 - 98,9	0	0,0 - 19,5	0.95	0,89 - 1,01		
>-4718	93.1	83,3 - 98,1	0	0,0 - 19,5	0.93	0,87 - 1,00		
>-4463	91.38	81,0 - 97,1	0	0,0 - 19,5	0.91	0,84 - 0,99		
>-3732	91.38	81,0 - 97,1	5.88	0,1 - 28,7	0.97	0,84 - 1,12	1.47	0,18 - 11,71
>-2727	89.66	78,8 - 96,1	5.88	0,1 - 28,7	0.95	0,82 - 1,10	1.76	0,23 - 13,62
>-2708	87.93	76,7 - 95,0	5.88	0,1 - 28,7	0.93	0,80 - 1,09	2.05	0,27 - 15,53
>-2437	86.21	74,6 - 93,9	5.88	0,1 - 28,7	0.92	0,78 - 1,07	2.34	0,32 - 17,45
>-2164	84.48	72,6 - 92,7	5.88	0,1 - 28,7	0.9	0,76 - 1,06	2.64	0,36 - 19,38
>-2115	82.76	70,6 - 91,4	5.88	0,1 - 28,7	0.88	0,74 - 1,04	2.93	0,40 - 21,30
>-2106	81.03	68,6 - 90,1	5.88	0,1 - 28,7	0.86	0,72 - 1,02	3.22	0,45 - 23,22
>-2071	81.03	68,6 - 90,1	11.76	1,5 - 36,4	0.92	0,74 - 1,14	1.61	0,40 - 6,58
>-1855	79.31	66,6 - 88,8	11.76	1,5 - 36,4	0.9	0,72 - 1,12	1.76	0,44 - 7,10
>-1852	77.59	64,7 - 87,5	11.76	1,5 - 36,4	0.88	0,70 - 1,10	1.91	0,48 - 7,63
>-1824	75.86	62,8 - 86,1	11.76	1,5 - 36,4	0.86	0,69 - 1,08	2.05	0,52 - 8,15
>-1816	74.14	61,0 - 84,7	11.76	1,5 - 36,4	0.84	0,67 - 1,06	2.2	0,56 - 8,68
>-1785	72.41	59,1 - 83,3	11.76	1,5 - 36,4	0.82	0,65 - 1,04	2.34	0,60 - 9,20
>-1760	70.69	57,3 - 81,9	11.76	1,5 - 36,4	0.8	0,63 - 1,02	2.49	0,64 - 9,72
>-1759	68.97	55,5 - 80,5	11.76	1,5 - 36,4	0.78	0,61 - 1,00	2.64	0,68 - 10,25
>-1733	67.24	53,7 - 79,0	11.76	1,5 - 36,4	0.76	0,59 - 0,98	2.78	0,72 - 10,77
>-1729	65.52	51,9 - 77,5	11.76	1,5 - 36,4	0.74	0,58 - 0,96	2.93	0,76 - 11,30
>-1717	63.79	50,1 - 76,0	11.76	1,5 - 36,4	0.72	0,56 - 0,94	3.08	0,80 - 11,82

frontiers

>-1639	62.07	48,4 - 74,5	11.76	1,5 - 36,4	0.7	0,54 - 0,92	3.22	0,84 - 12,35
>-1632	60.34	46,6 - 73,0	11.76	1,5 - 36,4	0.68	0,52 - 0,90	3.37	0,88 - 12,87
>-1626	60.34	46,6 - 73,0	17.65	3,8 - 43,4	0.73	0,54 - 0,99	2.25	0,77 - 6,58
>-1513	58.62	44,9 - 71,4	17.65	3,8 - 43,4	0.71	0,52 - 0,97	2.34	0,80 - 6,85
>-1505	56.9	43,2 - 69,8	17.65	3,8 - 43,4	0.69	0,50 - 0,95	2.44	0,84 - 7,11
>-1501	56.9	43,2 - 69,8	23.53	6,8 - 49,9	0.74	0,53 - 1,05	1.83	0,74 - 4,54
>-1476	55.17	41,5 - 68,3	23.53	6,8 - 49,9	0.72	0,51 - 1,03	1.91	0,77 - 4,70
>-1453	53.45	39,9 - 66,7	23.53	6,8 - 49,9	0.7	0,49 - 1,00	1.98	0,80 - 4,87
>-1426	53.45	39,9 - 66,7	29.41	10,3 - 56,0	0.76	0,51 - 1,12	1.58	0,72 - 3,47
>-1418	53.45	39,9 - 66,7	35.29	14,2 - 61,7	0.83	0,54 - 1,26	1.32	0,65 - 2,66
>-1383	51.72	38,2 - 65,0	35.29	14,2 - 61,7	0.8	0,52 - 1,23	1.37	0,68 - 2,75
>-1362	50	36,6 - 63,4	35.29	14,2 - 61,7	0.77	0,50 - 1,19	1.42	0,71 - 2,83
>-1325	50	36,6 - 63,4	41.18	18,4 - 67,1	0.85	0,53 - 1,37	1.21	0,65 - 2,27
>-1306	48.28	35,0 - 61,8	41.18	18,4 - 67,1	0.82	0,51 - 1,32	1.26	0,68 - 2,34
>-1294	46.55	33,3 - 60,1	47.06	23,0 - 72,2	0.88	0,52 - 1,49	1.14	0,65 - 1,99
>-1266	46.55	33,3 - 60,1	52.94	27,8 - 77,0	0.99	0,56 - 1,76	1.01	0,61 - 1,68
>-1240	46.55	33,3 - 60,1	58.82	32,9 - 81,6	1.13	0,60 - 2,13	0.91	0,57 - 1,45
>-1226	46.55	33,3 - 60,1	64.71	38,3 - 85,8	1.32	0,65 - 2,66	0.83	0,54 - 1,26
>-1225	44.83	31,7 - 58,5	64.71	38,3 - 85,8	1.27	0,63 - 2,57	0.85	0,56 - 1,30
>-1187	44.83	31,7 - 58,5	70.59	44,0 - 89,7	1.52	0,69 - 3,36	0.78	0,53 - 1,15
>-1161	43.1	30,2 - 56,8	76.47	50,1 - 93,2	1.83	0,74 - 4,54	0.74	0,53 - 1,05
>-1128	41.38	28,6 - 55,1	76.47	50,1 - 93,2	1.76	0,71 - 4,37	0.77	0,55 - 1,08
>-1078	41.38	28,6 - 55,1	82.35	56,6 - 96,2	2.34	0,80 - 6,85	0.71	0,52 - 0,97
>-1064	39.66	27,0 - 53,4	88.24	63,6 - 98,5	3.37	0,88 - 12,87	0.68	0,52 - 0,90
>-1045	39.66	27,0 - 53,4	94.12	71,3 - 99,9	6.74	0,98 - 46,34	0.64	0,50 - 0,82

>-964	39.66	27,0 - 53,4	100	80,5 - 100,0			0.6	0,49 - 0,74
>-963	37.93	25,5 - 51,6	100	80,5 - 100,0			0.62	0,51 - 0,76
>-705	36.21	24,0 - 49,9	100	80,5 - 100,0			0.64	0,53 - 0,77
>-604	34.48	22,5 - 48,1	100	80,5 - 100,0			0.66	0,54 - 0,79
>-556	32.76	21,0 - 46,3	100	80,5 - 100,0			0.67	0,56 - 0,80
>-539	31.03	19,5 - 44,5	100	80,5 - 100,0			0.69	0,58 - 0,82
>-187	29.31	18,1 - 42,7	100	80,5 - 100,0			0.71	0,60 - 0,83
>-163	27.59	16,7 - 40,9	100	80,5 - 100,0			0.72	0,62 - 0,85
>-151	25.86	15,3 - 39,0	100	80,5 - 100,0			0.74	0,64 - 0,86
>-141	24.14	13,9 - 37,2	100	80,5 - 100,0			0.76	0,66 - 0,88
>164	22.41	12,5 - 35,3	100	80,5 - 100,0			0.78	0,68 - 0,89
>197	20.69	11,2 - 33,4	100	80,5 - 100,0			0.79	0,70 - 0,90
>458	18.97	9,9 - 31,4	100	80,5 - 100,0			0.81	0,72 - 0,92
>467	17.24	8,6 - 29,4	100	80,5 - 100,0			0.83	0,74 - 0,93
>497	15.52	7,3 - 27,4	100	80,5 - 100,0			0.84	0,76 - 0,94
>757	13.79	6,1 - 25,4	100	80,5 - 100,0			0.86	0,78 - 0,96
>1162	12.07	5,0 - 23,3	100	80,5 - 100,0			0.88	0,80 - 0,97
>1494	10.34	3,9 - 21,2	100	80,5 - 100,0			0.9	0,82 - 0,98
>1701	8.62	2,9 - 19,0	100	80,5 - 100,0			0.91	0,84 - 0,99
>2708	6.9	1,9 - 16,7	100	80,5 - 100,0			0.93	0,87 - 1,00
>4359	5.17	1,1 - 14,4	100	80,5 - 100,0			0.95	0,89 - 1,01
>10850	3.45	0,4 - 11,9	100	80,5 - 100,0			0.97	0,92 - 1,01
>11871	1.72	0,04 - 9,2	100	80,5 - 100,0			0.98	0,95 - 1,02
>13647	0	0,0 - 6,2	100	80,5 - 100,0			1	1,00 - 1,00

Monday, September 22, 2025 17:01 - MedCalc® version 23.3.7

6L

ROC curve

Variable	Peptid_21
Classification variable	status

Sample size	75
Positive group [a]	58 (77,33%)
Negative group [b]	17 (22,67%)

status = 1
[b] status = 0

Disease prevalence (%)	unknown

Area under the ROC curve (AUC)

Area under the ROC curve (AUC)	0.617
Standard Error [a]	0.0677
95% Confidence interval [b]	0,497 to 0,727
z statistic	1.722
Significance level P (Area=0.5)	0.085

[a] DeLong et al., 1988
[b] Binomial exact

Youden index

Youden index J	0.3276
Associated criterion	>28
Sensitivity	32.76
Specificity	100

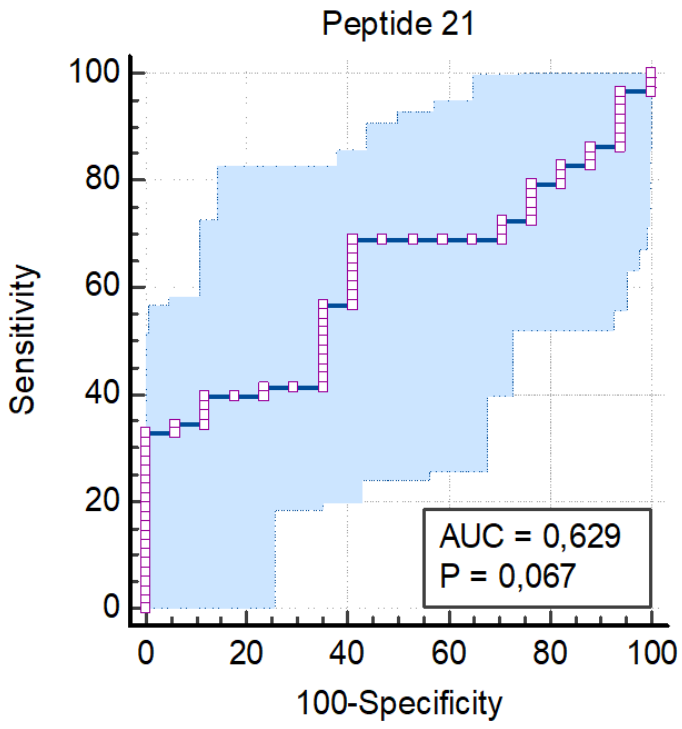

Criterion	Sensitivity	95% CI	Specificity	95% CI	+LR	95% CI	-LR	95% CI
≥-5164	100	93,8 - 100,0	0	0,0 - 19,5	1	1,00 - 1,00		
>-5164	98.28	90,8 - 100,0	0	0,0 - 19,5	0.98	0,95 - 1,02		
>-4433	96.55	88,1 - 99,6	0	0,0 - 19,5	0.97	0,92 - 1,01		
>-3540	96.55	88,1 - 99,6	5.88	0,1 - 28,7	1.03	0,90 - 1,17	0.59	0,057 - 6,08
>-3319	94.83	85,6 - 98,9	5.88	0,1 - 28,7	1.01	0,88 - 1,15	0.88	0,098 - 7,92
>-2137	93.1	83,3 - 98,1	5.88	0,1 - 28,7	0.99	0,86 - 1,14	1.17	0,14 - 9,80
>-1917	91.38	81,0 - 97,1	5.88	0,1 - 28,7	0.97	0,84 - 1,12	1.47	0,18 - 11,71
>-1781	89.66	78,8 - 96,1	5.88	0,1 - 28,7	0.95	0,82 - 1,10	1.76	0,23 - 13,62
>-1590	87.93	76,7 - 95,0	5.88	0,1 - 28,7	0.93	0,80 - 1,09	2.05	0,27 - 15,53
>-1564	86.21	74,6 - 93,9	5.88	0,1 - 28,7	0.92	0,78 - 1,07	2.34	0,32 - 17,45
>-1485	86.21	74,6 - 93,9	11.76	1,5 - 36,4	0.98	0,80 - 1,20	1.17	0,27 - 5,01
>-1360	84.48	72,6 - 92,7	11.76	1,5 - 36,4	0.96	0,78 - 1,18	1.32	0,31 - 5,53
>-1359	82.76	70,6 - 91,4	11.76	1,5 - 36,4	0.94	0,76 - 1,16	1.47	0,35 - 6,06
>-1309	82.76	70,6 - 91,4	17.65	3,8 - 43,4	1	0,78 - 1,29	0.98	0,30 - 3,15
>-1289	81.03	68,6 - 90,1	17.65	3,8 - 43,4	0.98	0,76 - 1,27	1.07	0,34 - 3,42
>-1214	79.31	66,6 - 88,8	17.65	3,8 - 43,4	0.96	0,75 - 1,24	1.17	0,37 - 3,68
>-1201	79.31	66,6 - 88,8	23.53	6,8 - 49,9	1.04	0,77 - 1,39	0.88	0,33 - 2,38
>-1199	77.59	64,7 - 87,5	23.53	6,8 - 49,9	1.01	0,75 - 1,37	0.95	0,36 - 2,54
>-1176	75.86	62,8 - 86,1	23.53	6,8 - 49,9	0.99	0,73 - 1,34	1.03	0,39 - 2,71
>-1157	74.14	61,0 - 84,7	23.53	6,8 - 49,9	0.97	0,72 - 1,31	1.1	0,42 - 2,87
>-1131	72.41	59,1 - 83,3	23.53	6,8 - 49,9	0.95	0,70 - 1,29	1.17	0,45 - 3,04
>-1123	72.41	59,1 - 83,3	29.41	10,3 - 56,0	1.03	0,73 - 1,45	0.94	0,40 - 2,19
>-1118	70.69	57,3 - 81,9	29.41	10,3 - 56,0	1	0,71 - 1,42	1	0,43 - 2,30
>-1107	68.97	55,5 - 80,5	29.41	10,3 - 56,0	0.98	0,69 - 1,39	1.06	0,46 - 2,42

>-1106	68.97	55,5 - 80,5	35.29	14,2 - 61,7	1.07	0,72 - 1,58	0.88	0,42 - 1,86
>-1097	68.97	55,5 - 80,5	41.18	18,4 - 67,1	1.17	0,76 - 1,81	0.75	0,38 - 1,50
>-1093	68.97	55,5 - 80,5	47.06	23,0 - 72,2	1.3	0,81 - 2,11	0.66	0,35 - 1,24
>-1059	68.97	55,5 - 80,5	52.94	27,8 - 77,0	1.47	0,86 - 2,50	0.59	0,32 - 1,06
>-1022	68.97	55,5 - 80,5	58.82	32,9 - 81,6	1.67	0,92 - 3,03	0.53	0,30 - 0,92
>-1020	67.24	53,7 - 79,0	58.82	32,9 - 81,6	1.63	0,90 - 2,96	0.56	0,32 - 0,96
>-1014	65.52	51,9 - 77,5	58.82	32,9 - 81,6	1.59	0,87 - 2,89	0.59	0,34 - 1,00
>-1008	63.79	50,1 - 76,0	58.82	32,9 - 81,6	1.55	0,85 - 2,82	0.62	0,36 - 1,04
>-1007	62.07	48,4 - 74,5	58.82	32,9 - 81,6	1.51	0,83 - 2,75	0.64	0,38 - 1,08
>-979	60.34	46,6 - 73,0	58.82	32,9 - 81,6	1.47	0,80 - 2,68	0.67	0,41 - 1,12
>-933	58.62	44,9 - 71,4	58.82	32,9 - 81,6	1.42	0,78 - 2,61	0.7	0,43 - 1,16
>-914	56.9	43,2 - 69,8	58.82	32,9 - 81,6	1.38	0,75 - 2,54	0.73	0,45 - 1,20
>-913	56.9	43,2 - 69,8	64.71	38,3 - 85,8	1.61	0,82 - 3,19	0.67	0,42 - 1,05
>-853	55.17	41,5 - 68,3	64.71	38,3 - 85,8	1.56	0,79 - 3,10	0.69	0,44 - 1,09
>-848	53.45	39,9 - 66,7	64.71	38,3 - 85,8	1.51	0,76 - 3,01	0.72	0,46 - 1,12
>-791	51.72	38,2 - 65,0	64.71	38,3 - 85,8	1.47	0,74 - 2,92	0.75	0,48 - 1,16
>-739	50	36,6 - 63,4	64.71	38,3 - 85,8	1.42	0,71 - 2,83	0.77	0,50 - 1,19
>-709	48.28	35,0 - 61,8	64.71	38,3 - 85,8	1.37	0,68 - 2,75	0.8	0,52 - 1,23
>-700	46.55	33,3 - 60,1	64.71	38,3 - 85,8	1.32	0,65 - 2,66	0.83	0,54 - 1,26
>-576	44.83	31,7 - 58,5	64.71	38,3 - 85,8	1.27	0,63 - 2,57	0.85	0,56 - 1,30
>-575	43.1	30,2 - 56,8	64.71	38,3 - 85,8	1.22	0,60 - 2,48	0.88	0,58 - 1,33
>-524	41.38	28,6 - 55,1	64.71	38,3 - 85,8	1.17	0,57 - 2,39	0.91	0,60 - 1,37
>-507	41.38	28,6 - 55,1	70.59	44,0 - 89,7	1.41	0,63 - 3,12	0.83	0,57 - 1,21
>-458	41.38	28,6 - 55,1	76.47	50,1 - 93,2	1.76	0,71 - 4,37	0.77	0,55 - 1,08
>-444	39.66	27,0 - 53,4	76.47	50,1 - 93,2	1.69	0,68 - 4,20	0.79	0,56 - 1,10

>-325	39.66	27,0 - 53,4	82.35	56,6 - 96,2	2.25	0,77 - 6,58	0.73	0,54 - 0,99
>-319	39.66	27,0 - 53,4	88.24	63,6 - 98,5	3.37	0,88 - 12,87	0.68	0,52 - 0,90
>-273	37.93	25,5 - 51,6	88.24	63,6 - 98,5	3.22	0,84 - 12,35	0.7	0,54 - 0,92
>-266	36.21	24,0 - 49,9	88.24	63,6 - 98,5	3.08	0,80 - 11,82	0.72	0,56 - 0,94
>-159	34.48	22,5 - 48,1	88.24	63,6 - 98,5	2.93	0,76 - 11,30	0.74	0,58 - 0,96
>-106	34.48	22,5 - 48,1	94.12	71,3 - 99,9	5.86	0,85 - 40,56	0.7	0,56 - 0,87
>-87	32.76	21,0 - 46,3	94.12	71,3 - 99,9	5.57	0,80 - 38,63	0.71	0,58 - 0,89
>28	32.76	21,0 - 46,3	100	80,5 - 100,0			0.67	0,56 - 0,80
>62	31.03	19,5 - 44,5	100	80,5 - 100,0			0.69	0,58 - 0,82
>216	29.31	18,1 - 42,7	100	80,5 - 100,0			0.71	0,60 - 0,83
>399	27.59	16,7 - 40,9	100	80,5 - 100,0			0.72	0,62 - 0,85
>877	25.86	15,3 - 39,0	100	80,5 - 100,0			0.74	0,64 - 0,86
>1009	24.14	13,9 - 37,2	100	80,5 - 100,0			0.76	0,66 - 0,88
>1407	22.41	12,5 - 35,3	100	80,5 - 100,0			0.78	0,68 - 0,89
>1536	20.69	11,2 - 33,4	100	80,5 - 100,0			0.79	0,70 - 0,90
>1568	18.97	9,9 - 31,4	100	80,5 - 100,0			0.81	0,72 - 0,92
>1774	17.24	8,6 - 29,4	100	80,5 - 100,0			0.83	0,74 - 0,93
>1923	15.52	7,3 - 27,4	100	80,5 - 100,0			0.84	0,76 - 0,94
>2119	13.79	6,1 - 25,4	100	80,5 - 100,0			0.86	0,78 - 0,96
>2486	12.07	5,0 - 23,3	100	80,5 - 100,0			0.88	0,80 - 0,97
>3347	10.34	3,9 - 21,2	100	80,5 - 100,0			0.9	0,82 - 0,98
>3570	8.62	2,9 - 19,0	100	80,5 - 100,0			0.91	0,84 - 0,99
>4516	6.9	1,9 - 16,7	100	80,5 - 100,0			0.93	0,87 - 1,00
>5287	5.17	1,1 - 14,4	100	80,5 - 100,0			0.95	0,89 - 1,01
>6457	3.45	0,4 - 11,9	100	80,5 - 100,0			0.97	0,92 - 1,01
>7093	1.72	0,04 - 9,2	100	80,5 - 100,0			0.98	0,95 - 1,02
>15178	0	0,0 - 6,2	100	80,5 - 100,0			1	1,00 - 1,00

Monday, September 22, 2025 17:06 - MedCalc® version 23.3.7

6M

frontiers

ROC curve

Variable	Peptid_22
Classification variable	status

Sample size	75
Positive group [a]	58 (77,33%)
Negative group [b]	17 (22,67%)

status = 1
[b] status = 0

Disease prevalence (%)	unknown

Area under the ROC curve (AUC)

Area under the ROC curve (AUC)	0.568
Standard Error [a]	0.0809
95% Confidence interval [b]	0,448 to 0,682
z statistic	0.84
Significance level P (Area=0.5)	0.4012

[a] DeLong et al., 1988
[b] Binomial exact

Youden index

Youden index J	0.2363
Associated criterion	>-647
Sensitivity	70.69
Specificity	52.94

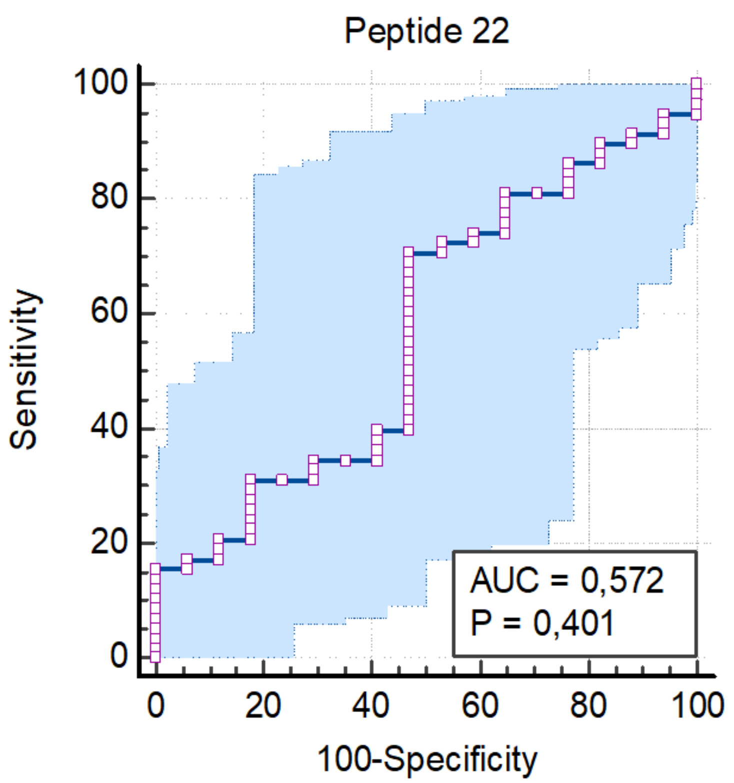

Criterion	Sensitivity	95% CI	Specificity	95% CI	+LR	95% CI	-LR	95% CI
≥-10640	100	93,8 - 100,0	0	0,0 - 19,5	1	1,00 - 1,00		
>-10640	98.28	90,8 - 100,0	0	0,0 - 19,5	0.98	0,95 - 1,02		
>-10238	96.55	88,1 - 99,6	0	0,0 - 19,5	0.97	0,92 - 1,01		
>-2303	94.83	85,6 - 98,9	0	0,0 - 19,5	0.95	0,89 - 1,01		
>-2285	94.83	85,6 - 98,9	5.88	0,1 - 28,7	1.01	0,88 - 1,15	0.88	0,098 - 7,92
>-1753	93.1	83,3 - 98,1	5.88	0,1 - 28,7	0.99	0,86 - 1,14	1.17	0,14 - 9,80
>-1496	91.38	81,0 - 97,1	5.88	0,1 - 28,7	0.97	0,84 - 1,12	1.47	0,18 - 11,71
>-1405	91.38	81,0 - 97,1	11.76	1,5 - 36,4	1.04	0,86 - 1,25	0.73	0,16 - 3,45
>-1289	89.66	78,8 - 96,1	11.76	1,5 - 36,4	1.02	0,84 - 1,23	0.88	0,19 - 3,97
>-1274	89.66	78,8 - 96,1	17.65	3,8 - 43,4	1.09	0,86 - 1,38	0.59	0,16 - 2,10
>-1227	87.93	76,7 - 95,0	17.65	3,8 - 43,4	1.07	0,84 - 1,36	0.68	0,20 - 2,36
>-1139	86.21	74,6 - 93,9	17.65	3,8 - 43,4	1.05	0,82 - 1,33	0.78	0,23 - 2,63
>-1135	86.21	74,6 - 93,9	23.53	6,8 - 49,9	1.13	0,85 - 1,50	0.59	0,20 - 1,71
>-1130	84.48	72,6 - 92,7	23.53	6,8 - 49,9	1.1	0,83 - 1,47	0.66	0,23 - 1,88
>-1125	82.76	70,6 - 91,4	23.53	6,8 - 49,9	1.08	0,81 - 1,44	0.73	0,26 - 2,04
>-1077	81.03	68,6 - 90,1	23.53	6,8 - 49,9	1.06	0,79 - 1,42	0.81	0,29 - 2,21
>-1049	81.03	68,6 - 90,1	29.41	10,3 - 56,0	1.15	0,82 - 1,60	0.64	0,26 - 1,60
>-1037	81.03	68,6 - 90,1	35.29	14,2 - 61,7	1.25	0,86 - 1,82	0.54	0,23 - 1,24
>-1035	79.31	66,6 - 88,8	35.29	14,2 - 61,7	1.23	0,84 - 1,78	0.59	0,26 - 1,33
>-1023	77.59	64,7 - 87,5	35.29	14,2 - 61,7	1.2	0,82 - 1,75	0.64	0,28 - 1,42
>-1018	75.86	62,8 - 86,1	35.29	14,2 - 61,7	1.17	0,80 - 1,71	0.68	0,31 - 1,51
>-973	74.14	61,0 - 84,7	35.29	14,2 - 61,7	1.15	0,78 - 1,68	0.73	0,34 - 1,59
>-971	74.14	61,0 - 84,7	41.18	18,4 - 67,1	1.26	0,82 - 1,93	0.63	0,31 - 1,29
>-964	72.41	59,1 - 83,3	41.18	18,4 - 67,1	1.23	0,80 - 1,89	0.67	0,33 - 1,36

>-815	72.41	59,1 - 83,3	47.06	23,0 - 72,2	1.37	0,85 - 2,20	0.59	0,30 - 1,13
>-696	70.69	57,3 - 81,9	47.06	23,0 - 72,2	1.34	0,83 - 2,15	0.62	0,33 - 1,19
>-647	70.69	57,3 - 81,9	52.94	27,8 - 77,0	1.5	0,88 - 2,55	0.55	0,30 - 1,01
>-594	68.97	55,5 - 80,5	52.94	27,8 - 77,0	1.47	0,86 - 2,50	0.59	0,32 - 1,06
>-548	67.24	53,7 - 79,0	52.94	27,8 - 77,0	1.43	0,84 - 2,44	0.62	0,35 - 1,11
>-463	65.52	51,9 - 77,5	52.94	27,8 - 77,0	1.39	0,81 - 2,38	0.65	0,37 - 1,15
>-453	63.79	50,1 - 76,0	52.94	27,8 - 77,0	1.36	0,79 - 2,33	0.68	0,39 - 1,20
>-358	62.07	48,4 - 74,5	52.94	27,8 - 77,0	1.32	0,77 - 2,27	0.72	0,41 - 1,25
>-258	60.34	46,6 - 73,0	52.94	27,8 - 77,0	1.28	0,74 - 2,21	0.75	0,43 - 1,30
>-190	58.62	44,9 - 71,4	52.94	27,8 - 77,0	1.25	0,72 - 2,16	0.78	0,45 - 1,35
>-150	56.9	43,2 - 69,8	52.94	27,8 - 77,0	1.21	0,70 - 2,10	0.81	0,48 - 1,39
>-116	55.17	41,5 - 68,3	52.94	27,8 - 77,0	1.17	0,67 - 2,04	0.85	0,50 - 1,44
>-80	53.45	39,9 - 66,7	52.94	27,8 - 77,0	1.14	0,65 - 1,99	0.88	0,52 - 1,49
>-54	51.72	38,2 - 65,0	52.94	27,8 - 77,0	1.1	0,63 - 1,93	0.91	0,54 - 1,54
>-53	50	36,6 - 63,4	52.94	27,8 - 77,0	1.06	0,60 - 1,87	0.94	0,56 - 1,58
>-41	48.28	35,0 - 61,8	52.94	27,8 - 77,0	1.03	0,58 - 1,81	0.98	0,59 - 1,63
>-31	46.55	33,3 - 60,1	52.94	27,8 - 77,0	0.99	0,56 - 1,76	1.01	0,61 - 1,68
>-22	44.83	31,7 - 58,5	52.94	27,8 - 77,0	0.95	0,53 - 1,70	1.04	0,63 - 1,73
>6	43.1	30,2 - 56,8	52.94	27,8 - 77,0	0.92	0,51 - 1,64	1.07	0,65 - 1,77
>24	41.38	28,6 - 55,1	52.94	27,8 - 77,0	0.88	0,49 - 1,59	1.11	0,67 - 1,82
>112	39.66	27,0 - 53,4	52.94	27,8 - 77,0	0.84	0,46 - 1,53	1.14	0,70 - 1,87
>182	39.66	27,0 - 53,4	58.82	32,9 - 81,6	0.96	0,50 - 1,85	1.03	0,65 - 1,61
>213	37.93	25,5 - 51,6	58.82	32,9 - 81,6	0.92	0,48 - 1,78	1.06	0,68 - 1,65
>240	36.21	24,0 - 49,9	58.82	32,9 - 81,6	0.88	0,45 - 1,71	1.08	0,70 - 1,69
>409	34.48	22,5 - 48,1	58.82	32,9 - 81,6	0.84	0,43 - 1,64	1.11	0,72 - 1,73

>460	34.48	22,5 - 48,1	64.71	38,3 - 85,8	0.98	0,47 - 2,04	1.01	0,68 - 1,51
>507	34.48	22,5 - 48,1	70.59	44,0 - 89,7	1.17	0,52 - 2,66	0.93	0,65 - 1,33
>603	32.76	21,0 - 46,3	70.59	44,0 - 89,7	1.11	0,49 - 2,54	0.95	0,67 - 1,36
>749	31.03	19,5 - 44,5	70.59	44,0 - 89,7	1.06	0,46 - 2,42	0.98	0,69 - 1,39
>996	31.03	19,5 - 44,5	76.47	50,1 - 93,2	1.32	0,52 - 3,37	0.9	0,66 - 1,24
>1042	31.03	19,5 - 44,5	82.35	56,6 - 96,2	1.76	0,59 - 5,26	0.84	0,63 - 1,11
>1163	29.31	18,1 - 42,7	82.35	56,6 - 96,2	1.66	0,55 - 5,00	0.86	0,65 - 1,13
>1580	27.59	16,7 - 40,9	82.35	56,6 - 96,2	1.56	0,52 - 4,74	0.88	0,67 - 1,15
>1673	25.86	15,3 - 39,0	82.35	56,6 - 96,2	1.47	0,48 - 4,47	0.9	0,69 - 1,18
>2275	24.14	13,9 - 37,2	82.35	56,6 - 96,2	1.37	0,44 - 4,21	0.92	0,71 - 1,20
>2674	22.41	12,5 - 35,3	82.35	56,6 - 96,2	1.27	0,41 - 3,94	0.94	0,73 - 1,22
>2703	20.69	11,2 - 33,4	82.35	56,6 - 96,2	1.17	0,37 - 3,68	0.96	0,75 - 1,24
>2978	20.69	11,2 - 33,4	88.24	63,6 - 98,5	1.76	0,44 - 7,10	0.9	0,72 - 1,12
>2983	18.97	9,9 - 31,4	88.24	63,6 - 98,5	1.61	0,40 - 6,58	0.92	0,74 - 1,14
>3328	17.24	8,6 - 29,4	88.24	63,6 - 98,5	1.47	0,35 - 6,06	0.94	0,76 - 1,16
>4370	17.24	8,6 - 29,4	94.12	71,3 - 99,9	2.93	0,40 - 21,30	0.88	0,74 - 1,04
>4429	15.52	7,3 - 27,4	94.12	71,3 - 99,9	2.64	0,36 - 19,38	0.9	0,76 - 1,06
>4666	15.52	7,3 - 27,4	100	80,5 - 100,0			0.84	0,76 - 0,94
>5189	13.79	6,1 - 25,4	100	80,5 - 100,0			0.86	0,78 - 0,96
>6001	12.07	5,0 - 23,3	100	80,5 - 100,0			0.88	0,80 - 0,97
>6565	10.34	3,9 - 21,2	100	80,5 - 100,0			0.9	0,82 - 0,98
>7075	8.62	2,9 - 19,0	100	80,5 - 100,0			0.91	0,84 - 0,99
>10235	6.9	1,9 - 16,7	100	80,5 - 100,0			0.93	0,87 - 1,00
>11042	5.17	1,1 - 14,4	100	80,5 - 100,0			0.95	0,89 - 1,01
>12137	3.45	0,4 - 11,9	100	80,5 - 100,0			0.97	0,92 - 1,01
>13389	1.72	0,04 - 9,2	100	80,5 - 100,0			0.98	0,95 - 1,02
>46459	0	0,0 - 6,2	100	80,5 - 100,0			1	1,00 - 1,00

Monday, September 22, 2025 17:07 - MedCalc® version 23.3.7

6N

ROC curve

Variable	Peptid_25
Classification variable	status

Sample size	75
Positive group [a]	58 (77,33%)
Negative group [b]	17 (22,67%)

status = 1
[b] status = 0

Disease prevalence (%)	unknown

Area under the ROC curve (AUC)

Area under the ROC curve (AUC)	0.69
Standard Error [a]	0.0714
95% Confidence interval [b]	0,572 to 0,792
z statistic	2.659
Significance level P (Area=0.5)	0.0078

[a] DeLong et al., 1988
[b] Binomial exact

Youden index

Youden index J	0.5666
Associated criterion	≤-1112
Sensitivity	68.42
Specificity	88.24

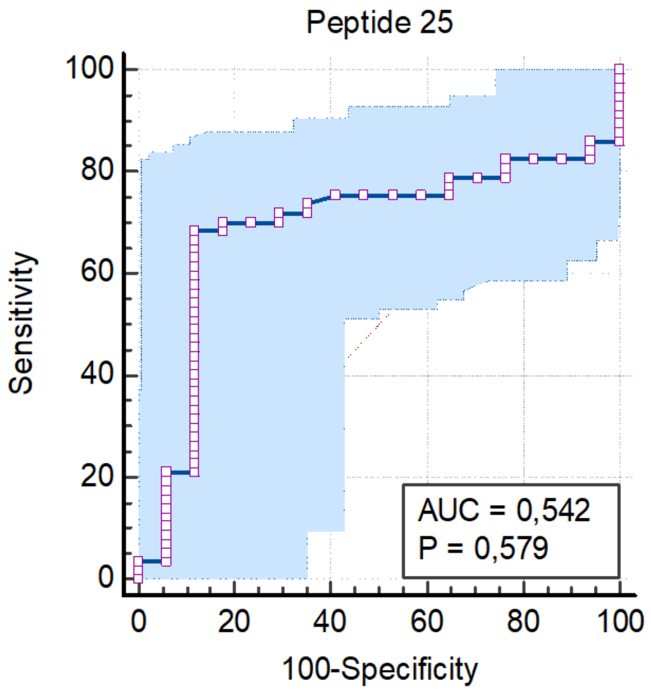

frontiers

Criterion	Sensitivity	95% CI	Specificity	95% CI	+LR	95% CI	-LR	95% CI
<-8977	0	0,0 - 6,3	100	80,5 - 100,0			1	1,00 - 1,00
≤-8977	1.75	0,04 - 9,4	100	80,5 - 100,0			0.98	0,95 - 1,02
≤-6479	3.51	0,4 - 12,1	100	80,5 - 100,0			0.96	0,92 - 1,01
≤-4446	3.51	0,4 - 12,1	94.12	71,3 - 99,9	0.6	0,058 - 6,18	1.03	0,90 - 1,17
≤-3856	5.26	1,1 - 14,6	94.12	71,3 - 99,9	0.89	0,099 - 8,05	1.01	0,88 - 1,15
≤-3781	7.02	1,9 - 17,0	94.12	71,3 - 99,9	1.19	0,14 - 9,97	0.99	0,86 - 1,13
≤-3689	8.77	2,9 - 19,3	94.12	71,3 - 99,9	1.49	0,19 - 11,91	0.97	0,84 - 1,12
≤-3418	10.53	4,0 - 21,5	94.12	71,3 - 99,9	1.79	0,23 - 13,85	0.95	0,82 - 1,10
≤-3131	12.28	5,1 - 23,7	94.12	71,3 - 99,9	2.09	0,28 - 15,80	0.93	0,80 - 1,09
≤-3045	14.04	6,3 - 25,8	94.12	71,3 - 99,9	2.39	0,32 - 17,76	0.91	0,78 - 1,07
≤-2538	15.79	7,5 - 27,9	94.12	71,3 - 99,9	2.68	0,37 - 19,71	0.89	0,76 - 1,05
≤-2224	17.54	8,7 - 29,9	94.12	71,3 - 99,9	2.98	0,41 - 21,67	0.88	0,74 - 1,04
≤-2168	19.3	10,0 - 31,9	94.12	71,3 - 99,9	3.28	0,46 - 23,62	0.86	0,72 - 1,02
≤-2090	21.05	11,4 - 33,9	94.12	71,3 - 99,9	3.58	0,50 - 25,58	0.84	0,70 - 1,00
≤-1892	21.05	11,4 - 33,9	88.24	63,6 - 98,5	1.79	0,44 - 7,22	0.89	0,72 - 1,11
≤-1811	22.81	12,7 - 35,8	88.24	63,6 - 98,5	1.94	0,48 - 7,76	0.87	0,70 - 1,09
≤-1747	24.56	14,1 - 37,8	88.24	63,6 - 98,5	2.09	0,53 - 8,29	0.85	0,68 - 1,07
≤-1631	26.32	15,5 - 39,7	88.24	63,6 - 98,5	2.24	0,57 - 8,82	0.84	0,66 - 1,05
≤-1594	28.07	17,0 - 41,5	88.24	63,6 - 98,5	2.39	0,61 - 9,36	0.82	0,64 - 1,03
≤-1581	29.82	18,4 - 43,4	88.24	63,6 - 98,5	2.54	0,65 - 9,89	0.8	0,62 - 1,01
≤-1539	31.58	19,9 - 45,2	88.24	63,6 - 98,5	2.68	0,69 - 10,42	0.78	0,61 - 0,99
≤-1507	33.33	21,4 - 47,1	88.24	63,6 - 98,5	2.83	0,73 - 10,96	0.76	0,59 - 0,97
≤-1496	35.09	22,9 - 48,9	88.24	63,6 - 98,5	2.98	0,77 - 11,49	0.74	0,57 - 0,95
≤-1493	36.84	24,4 - 50,7	88.24	63,6 - 98,5	3.13	0,82 - 12,03	0.72	0,55 - 0,93

≤-1490	38.6	26,0 - 52,4	88.24	63,6 - 98,5	3.28	0,86 - 12,56	0.7	0,53 - 0,91
≤-1486	40.35	27,6 - 54,2	88.24	63,6 - 98,5	3.43	0,90 - 13,09	0.68	0,51 - 0,89
≤-1431	42.11	29,1 - 55,9	88.24	63,6 - 98,5	3.58	0,94 - 13,63	0.66	0,50 - 0,87
≤-1429	43.86	30,7 - 57,6	88.24	63,6 - 98,5	3.73	0,98 - 14,16	0.64	0,48 - 0,85
≤-1426	45.61	32,4 - 59,3	88.24	63,6 - 98,5	3.88	1,02 - 14,69	0.62	0,46 - 0,83
≤-1402	47.37	34,0 - 61,0	88.24	63,6 - 98,5	4.03	1,06 - 15,23	0.6	0,44 - 0,81
≤-1398	49.12	35,6 - 62,7	88.24	63,6 - 98,5	4.18	1,11 - 15,76	0.58	0,42 - 0,79
≤-1394	50.88	37,3 - 64,4	88.24	63,6 - 98,5	4.32	1,15 - 16,30	0.56	0,41 - 0,76
≤-1385	52.63	39,0 - 66,0	88.24	63,6 - 98,5	4.47	1,19 - 16,83	0.54	0,39 - 0,74
≤-1352	54.39	40,7 - 67,6	88.24	63,6 - 98,5	4.62	1,23 - 17,36	0.52	0,37 - 0,72
≤-1320	56.14	42,4 - 69,3	88.24	63,6 - 98,5	4.77	1,27 - 17,90	0.5	0,35 - 0,70
≤-1316	57.89	44,1 - 70,9	88.24	63,6 - 98,5	4.92	1,31 - 18,43	0.48	0,34 - 0,68
≤-1312	59.65	45,8 - 72,4	88.24	63,6 - 98,5	5.07	1,36 - 18,97	0.46	0,32 - 0,66
≤-1239	61.4	47,6 - 74,0	88.24	63,6 - 98,5	5.22	1,40 - 19,50	0.44	0,30 - 0,63
≤-1214	63.16	49,3 - 75,6	88.24	63,6 - 98,5	5.37	1,44 - 20,03	0.42	0,29 - 0,61
≤-1209	64.91	51,1 - 77,1	88.24	63,6 - 98,5	5.52	1,48 - 20,57	0.4	0,27 - 0,59
≤-1178	66.67	52,9 - 78,6	88.24	63,6 - 98,5	5.67	1,52 - 21,10	0.38	0,25 - 0,57
≤-1112	68.42	54,8 - 80,1	88.24	63,6 - 98,5	5.82	1,56 - 21,64	0.36	0,24 - 0,54
≤-1058	68.42	54,8 - 80,1	82.35	56,6 - 96,2	3.88	1,37 - 10,99	0.38	0,25 - 0,60
≤-1009	70.18	56,6 - 81,6	82.35	56,6 - 96,2	3.98	1,40 - 11,26	0.36	0,23 - 0,57
≤-1005	70.18	56,6 - 81,6	76.47	50,1 - 93,2	2.98	1,25 - 7,14	0.39	0,24 - 0,63
≤-873	70.18	56,6 - 81,6	70.59	44,0 - 89,7	2.39	1,12 - 5,08	0.42	0,26 - 0,70
≤-857	71.93	58,5 - 83,0	70.59	44,0 - 89,7	2.45	1,15 - 5,20	0.4	0,24 - 0,67
≤-833	71.93	58,5 - 83,0	64.71	38,3 - 85,8	2.04	1,05 - 3,96	0.43	0,25 - 0,75
≤-807	73.68	60,3 - 84,5	64.71	38,3 - 85,8	2.09	1,08 - 4,05	0.41	0,23 - 0,71

≤-795	75.44	62,2 - 85,9	58.82	32,9 - 81,6	1.83	1,02 - 3,30	0.42	0,23 - 0,76
≤-757	75.44	62,2 - 85,9	52.94	27,8 - 77,0	1.6	0,95 - 2,71	0.46	0,24 - 0,88
≤-727	75.44	62,2 - 85,9	47.06	23,0 - 72,2	1.42	0,89 - 2,28	0.52	0,26 - 1,03
≤-698	75.44	62,2 - 85,9	41.18	18,4 - 67,1	1.28	0,84 - 1,96	0.6	0,29 - 1,24
≤-598	75.44	62,2 - 85,9	35.29	14,2 - 61,7	1.17	0,80 - 1,71	0.7	0,32 - 1,53
≤-593	77.19	64,2 - 87,3	35.29	14,2 - 61,7	1.19	0,82 - 1,74	0.65	0,29 - 1,44
≤-590	78.95	66,1 - 88,6	35.29	14,2 - 61,7	1.22	0,84 - 1,78	0.6	0,26 - 1,35
≤-538	78.95	66,1 - 88,6	29.41	10,3 - 56,0	1.12	0,80 - 1,56	0.72	0,29 - 1,75
≤-375	78.95	66,1 - 88,6	23.53	6,8 - 49,9	1.03	0,77 - 1,39	0.89	0,33 - 2,42
≤-300	80.7	68,1 - 90,0	23.53	6,8 - 49,9	1.06	0,79 - 1,41	0.82	0,30 - 2,25
≤-251	82.46	70,1 - 91,3	23.53	6,8 - 49,9	1.08	0,81 - 1,44	0.75	0,27 - 2,08
≤-1,507283091	82.46	70,1 - 91,3	17.65	3,8 - 43,4	1	0,78 - 1,29	0.99	0,31 - 3,21
≤9,336975416	82.46	70,1 - 91,3	11.76	1,5 - 36,4	0.93	0,76 - 1,15	1.49	0,36 - 6,16
≤15,45848823	82.46	70,1 - 91,3	5.88	0,1 - 28,7	0.88	0,74 - 1,04	2.98	0,41 - 21,67
≤25	84.21	72,1 - 92,5	5.88	0,1 - 28,7	0.89	0,76 - 1,05	2.68	0,37 - 19,71
≤617	85.96	74,2 - 93,7	5.88	0,1 - 28,7	0.91	0,78 - 1,07	2.39	0,32 - 17,76
≤655	85.96	74,2 - 93,7	0	0,0 - 19,5	0.86	0,77 - 0,95		
≤1477	87.72	76,3 - 94,9	0	0,0 - 19,5	0.88	0,80 - 0,97		
≤2611	89.47	78,5 - 96,0	0	0,0 - 19,5	0.89	0,82 - 0,98		
≤4077	91.23	80,7 - 97,1	0	0,0 - 19,5	0.91	0,84 - 0,99		
≤5110	92.98	83,0 - 98,1	0	0,0 - 19,5	0.93	0,87 - 1,00		
≤5470	94.74	85,4 - 98,9	0	0,0 - 19,5	0.95	0,89 - 1,01		
≤14933	96.49	87,9 - 99,6	0	0,0 - 19,5	0.96	0,92 - 1,01		
≤21697	98.25	90,6 - 100,0	0	0,0 - 19,5	0.98	0,95 - 1,02		
≤35319	100	93,7 - 100,0	0	0,0 - 19,5	1	1,00 - 1,00		

Monday, September 22, 2025 17:08 - MedCalc® version 23.3.7

6O

ROC curve

Variable	ELISA
Classification variable	status

Sample size	75
Positive group [a]	58 (77,33%)
Negative group [b]	17 (22,67%)

status = 1

[b] status = 0

Disease prevalence (%)	unknown

Area under the ROC curve (AUC)

Area under the ROC curve (AUC)	1
Standard Error [a]	0
95% Confidence interval [b]	0,952 to 1,000
Significance level P (Area=0.5)	<0,0001

[a] DeLong et al., 1988

[b] Binomial exact

Youden index

Youden index J	1
Associated criterion	>7,63
Sensitivity	100
Specificity	100

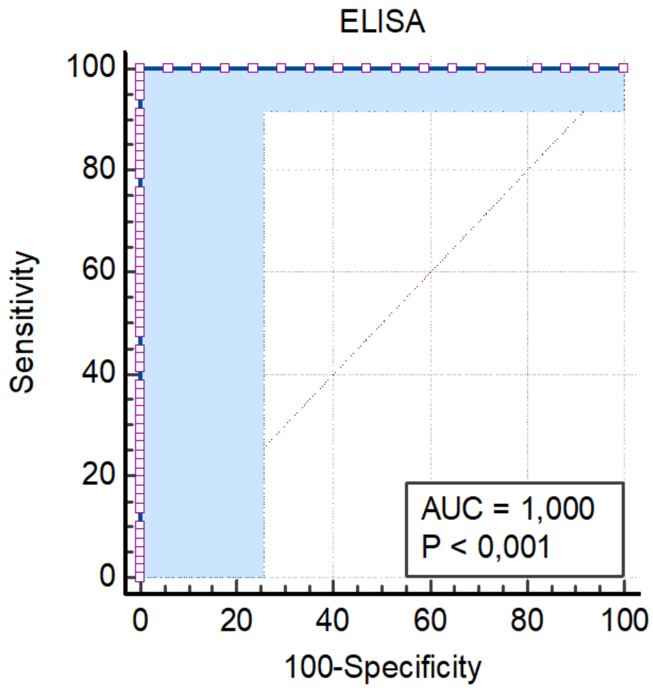

Criterion	Sensitivity	95% CI	Specificity	95% CI	+LR	95% CI	-LR	95% CI
≥-0,04	100	93,8 - 100,0	0	0,0 - 19,5	1	1,00 - 1,00		
>-0,04	100	93,8 - 100,0	5.88	0,1 - 28,7	1.06	0,94 - 1,20	0	
>0,62	100	93,8 - 100,0	11.76	1,5 - 36,4	1.13	0,95 - 1,35	0	
>0,66	100	93,8 - 100,0	17.65	3,8 - 43,4	1.21	0,97 - 1,51	0	
>0,71	100	93,8 - 100,0	29.41	10,3 - 56,0	1.42	1,04 - 1,93	0	
>1,18	100	93,8 - 100,0	35.29	14,2 - 61,7	1.55	1,09 - 2,20	0	
>1,19	100	93,8 - 100,0	41.18	18,4 - 67,1	1.7	1,14 - 2,53	0	
>1,28	100	93,8 - 100,0	47.06	23,0 - 72,2	1.89	1,21 - 2,96	0	
>1,68	100	93,8 - 100,0	52.94	27,8 - 77,0	2.12	1,28 - 3,52	0	
>1,94	100	93,8 - 100,0	58.82	32,9 - 81,6	2.43	1,38 - 4,29	0	
>2,34	100	93,8 - 100,0	64.71	38,3 - 85,8	2.83	1,49 - 5,39	0	
>2,43	100	93,8 - 100,0	70.59	44,0 - 89,7	3.4	1,63 - 7,10	0	
>3,17	100	93,8 - 100,0	76.47	50,1 - 93,2	4.25	1,80 - 10,01	0	
>3,48	100	93,8 - 100,0	82.35	56,6 - 96,2	5.67	2,03 - 15,82	0	
>3,66	100	93,8 - 100,0	88.24	63,6 - 98,5	8.5	2,31 - 31,25	0	
>6,66	100	93,8 - 100,0	94.12	71,3 - 99,9	17	2,54 - 113,83	0	
>7,63	100	93,8 - 100,0	100	80,5 - 100,0			0	
>20,63	98.28	90,8 - 100,0	100	80,5 - 100,0			0.017	0,0025 - 0,12
>36,61	96.55	88,1 - 99,6	100	80,5 - 100,0			0.034	0,0088 - 0,13
>48,13	94.83	85,6 - 98,9	100	80,5 - 100,0			0.052	0,017 - 0,16
>53,65	91.38	81,0 - 97,1	100	80,5 - 100,0			0.086	0,037 - 0,20
>55,76	89.66	78,8 - 96,1	100	80,5 - 100,0			0.1	0,048 - 0,22

>55,97	87.93	76,7 - 95,0	100	80,5 - 100,0			0.12	0,060 - 0,24
>56,59	86.21	74,6 - 93,9	100	80,5 - 100,0			0.14	0,072 - 0,26
>57,22	84.48	72,6 - 92,7	100	80,5 - 100,0			0.16	0,085 - 0,28
>62,56	82.76	70,6 - 91,4	100	80,5 - 100,0			0.17	0,098 - 0,30
>63,19	81.03	68,6 - 90,1	100	80,5 - 100,0			0.19	0,11 - 0,32
>65,89	79.31	66,6 - 88,8	100	80,5 - 100,0			0.21	0,13 - 0,34
>67,69	75.86	62,8 - 86,1	100	80,5 - 100,0			0.24	0,15 - 0,38
>67,83	74.14	61,0 - 84,7	100	80,5 - 100,0			0.26	0,17 - 0,40
>69,85	72.41	59,1 - 83,3	100	80,5 - 100,0			0.28	0,18 - 0,42
>70,73	70.69	57,3 - 81,9	100	80,5 - 100,0			0.29	0,20 - 0,44
>71,17	68.97	55,5 - 80,5	100	80,5 - 100,0			0.31	0,21 - 0,46
>73,25	67.24	53,7 - 79,0	100	80,5 - 100,0			0.33	0,23 - 0,47
>74,71	65.52	51,9 - 77,5	100	80,5 - 100,0			0.34	0,24 - 0,49
>75,93	63.79	50,1 - 76,0	100	80,5 - 100,0			0.36	0,26 - 0,51
>76,51	62.07	48,4 - 74,5	100	80,5 - 100,0			0.38	0,27 - 0,53
>77,2	60.34	46,6 - 73,0	100	80,5 - 100,0			0.4	0,29 - 0,54
>77,34	58.62	44,9 - 71,4	100	80,5 - 100,0			0.41	0,30 - 0,56
>86,39	56.9	43,2 - 69,8	100	80,5 - 100,0			0.43	0,32 - 0,58
>86,53	55.17	41,5 - 68,3	100	80,5 - 100,0			0.45	0,34 - 0,60
>87,77	53.45	39,9 - 66,7	100	80,5 - 100,0			0.47	0,35 - 0,61
>93,1	51.72	38,2 - 65,0	100	80,5 - 100,0			0.48	0,37 - 0,63
>93,35	50	36,6 - 63,4	100	80,5 - 100,0			0.5	0,39 - 0,65
>98,37	48.28	35,0 - 61,8	100	80,5 - 100,0			0.52	0,40 - 0,66
>98,95	44.83	31,7 - 58,5	100	80,5 - 100,0			0.55	0,44 - 0,70
>100,1	43.1	30,2 - 56,8	100	80,5 - 100,0			0.57	0,45 - 0,71

>100,45	41.38	28,6 - 55,1	100	80,5 - 100,0			0.59	0,47 - 0,73
>101,49	37.93	25,5 - 51,6	100	80,5 - 100,0			0.62	0,51 - 0,76
>106,38	36.21	24,0 - 49,9	100	80,5 - 100,0			0.64	0,53 - 0,77
>109,61	34.48	22,5 - 48,1	100	80,5 - 100,0			0.66	0,54 - 0,79
>111	32.76	21,0 - 46,3	100	80,5 - 100,0			0.67	0,56 - 0,80
>112,6	31.03	19,5 - 44,5	100	80,5 - 100,0			0.69	0,58 - 0,82
>112,8	29.31	18,1 - 42,7	100	80,5 - 100,0			0.71	0,60 - 0,83
>114,61	27.59	16,7 - 40,9	100	80,5 - 100,0			0.72	0,62 - 0,85
>119,54	25.86	15,3 - 39,0	100	80,5 - 100,0			0.74	0,64 - 0,86
>119,6	24.14	13,9 - 37,2	100	80,5 - 100,0			0.76	0,66 - 0,88
>120,92	22.41	12,5 - 35,3	100	80,5 - 100,0			0.78	0,68 - 0,89
>121,57	20.69	11,2 - 33,4	100	80,5 - 100,0			0.79	0,70 - 0,90
>123,13	18.97	9,9 - 31,4	100	80,5 - 100,0			0.81	0,72 - 0,92
>126,98	17.24	8,6 - 29,4	100	80,5 - 100,0			0.83	0,74 - 0,93
>129	15.52	7,3 - 27,4	100	80,5 - 100,0			0.84	0,76 - 0,94
>131,5	13.79	6,1 - 25,4	100	80,5 - 100,0			0.86	0,78 - 0,96
>134,27	10.34	3,9 - 21,2	100	80,5 - 100,0			0.9	0,82 - 0,98
>136,7	8.62	2,9 - 19,0	100	80,5 - 100,0			0.91	0,84 - 0,99
>143,45	6.9	1,9 - 16,7	100	80,5 - 100,0			0.93	0,87 - 1,00
>159,79	5.17	1,1 - 14,4	100	80,5 - 100,0			0.95	0,89 - 1,01
>165,73	3.45	0,4 - 11,9	100	80,5 - 100,0			0.97	0,92 - 1,01
>183,56	1.72	0,04 - 9,2	100	80,5 - 100,0			0.98	0,95 - 1,02
>211,72	0	0,0 - 6,2	100	80,5 - 100,0			1	1,00 - 1,00

Monday, September 22, 2025 17:08 - MedCalc® version 23.3.7

6P

**Variable 1**	ELISA
**Variable 2**	Multi6_sum
**Classification variable**	status

**Sample size**		74
**Positive group :**	**status = 1**	59
**Negative group :**	**status = 0**	15

	**AUC**	**SE [a]**	**95% CI [b]**
ELISA	0,973	0,0193	0,906 to 0,997
Multi6_sum	0,831	0,0629	0,726 to 0,908

[a] DeLong et al., 1988

[b] Binomial exact

Pairwise comparison of ROC curves

ELISA ~ Multi6_sum

**Difference between areas**	0,142
**Standard Error[c]**	0,0642
**95% Confidence Interval**	0,0160 to 0,268
**z statistic**	2,209
**Significance level**	P = 0,0272

[c] DeLong et al., 1988

MedCalc® version 14.8.1

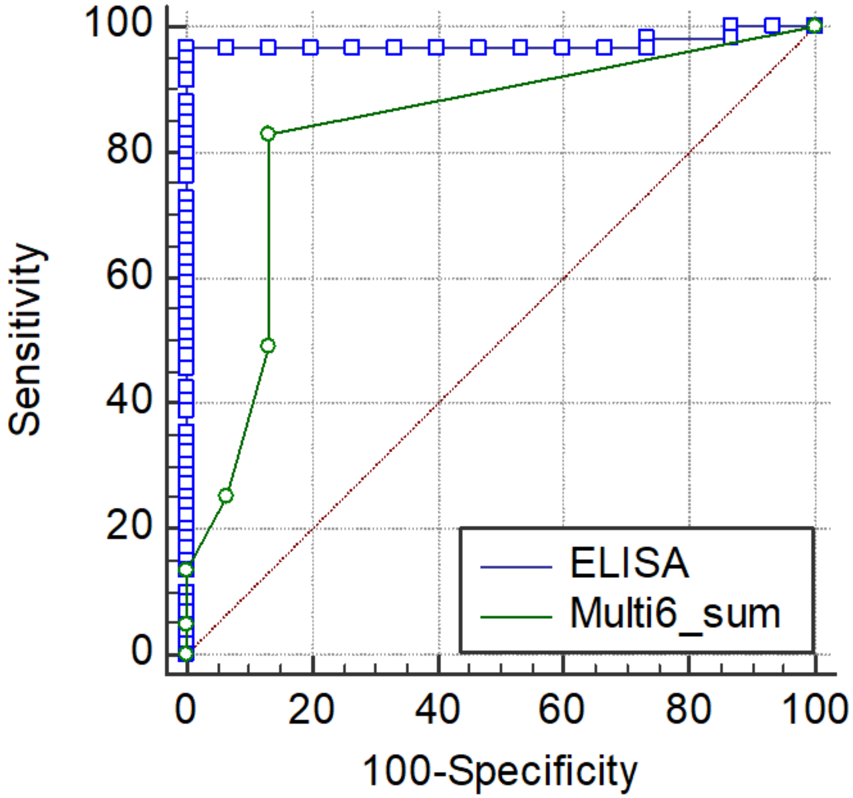

6Q

Variable 1	ELISA		
Variable 2	peptide_19		
Variable 3	peptide_25		
Classification variable	status		
Sample size		74	
Positive group :	status = 1	59	
Negative group :	status = 0	15	

	AUC	SE [a]	95% CI [b]
ELISA	0,973	0,0193	0,906 to 0,997
peptide_19	0,598	0,0667	0,478 to 0,711
peptide_25	0,664	0,0751	0,545 to 0,770

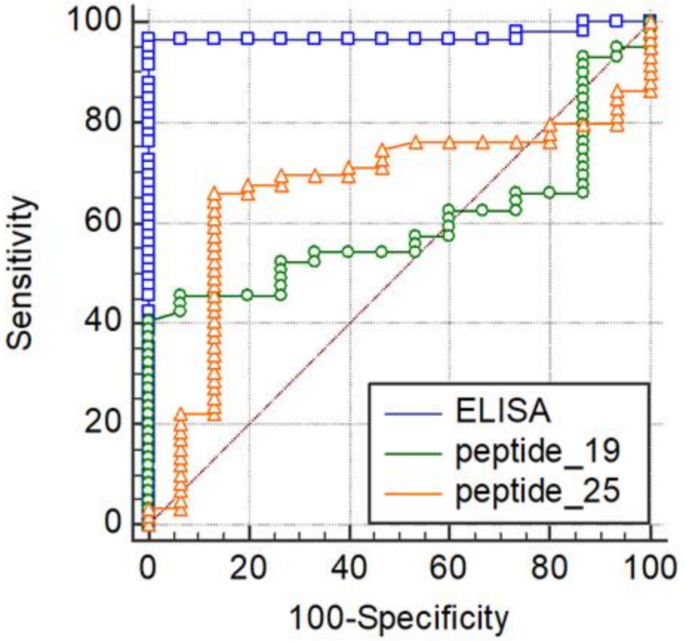

6R

Pairwise comparison of ROC curves

**ELISA ~ peptide_19**	
Difference between areas	0,375
Standard Error[c]	0,0719
95% Confidence Interval	0,234 to 0,515
z statistic	5,213
Significance level	P < 0,0001
**ELISA ~ peptide_25**	
Difference between areas	0,309
Standard Error[c]	0,0774
95% Confidence Interval	0,157 to 0,461
z statistic	3,995
Significance level	P = 0,0001
**peptide_19 ~ peptide_25**	
Difference between areas	0,0655
Standard Error[c]	0,115
95% Confidence Interval	-0,159 to 0,290
z statistic	0,571
Significance level	P = 0,5680

a DeLong et al., 1988
b Binomial exact
c DeLong et al., 1988

MedCalc® version 14.8.1

Variable 1	ELISA			
Variable 2	peptide_4			
Variable 3	peptide_5			
Classification variable	status			
Sample size			74	
Positive group :	status = 1		59	
Negative group :	status = 0		15	
		AUC	SE [a]	95% CI [b]
ELISA		0,973	0,0193	0,906 to 0,997
peptide_4		0,598	0,0762	0,418 to 0,655
peptide_5		0,664	0,0748	0,493 to 0,725

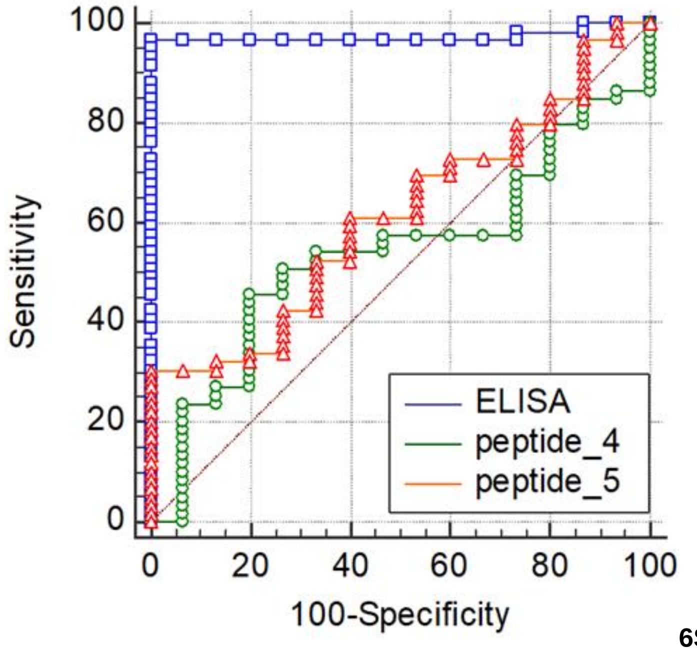

6S

Pairwise comparison of ROC curves

**ELISA ~ peptide_4**	
Difference between areas	0,435
Standard Error[c]	0,0798
95% Confidence Interval	0,279 to 0,591
z statistic	5,452
Significance level	P < 0,0001
**ELISA ~ peptide_5**	
Difference between areas	0,359
Standard Error[c]	0,0785
95% Confidence Interval	0,205 to 0,513
z statistic	4,576
Significance level	P = 0,0001
**peptide_4 ~ peptide_5**	
Difference between areas	0,0757
Standard Error[c]	0,107
95% Confidence Interval	-0,134 to 0,286
z statistic	0,707
Significance level	P = 0,4796

a DeLong et al., 1988
b Binomial exact
c DeLong et al., 1988

MedCalc® version 14.8.1

Variable 1	ELISA			
Variable 2	Peptide6			
Variable 3	peptide_11			
Classification variable	status			
Sample size			74	
Positive group :	status = 1		59	
Negative group :	status = 0		15	
		AUC	SE [a]	95% CI [b]
ELISA		0,973	0,0193	0,906 to 0,997
peptide_4		0,714	0,0640	0,597 to 0,813
peptide_5		0,616	0,0705	0,495 to 0,727

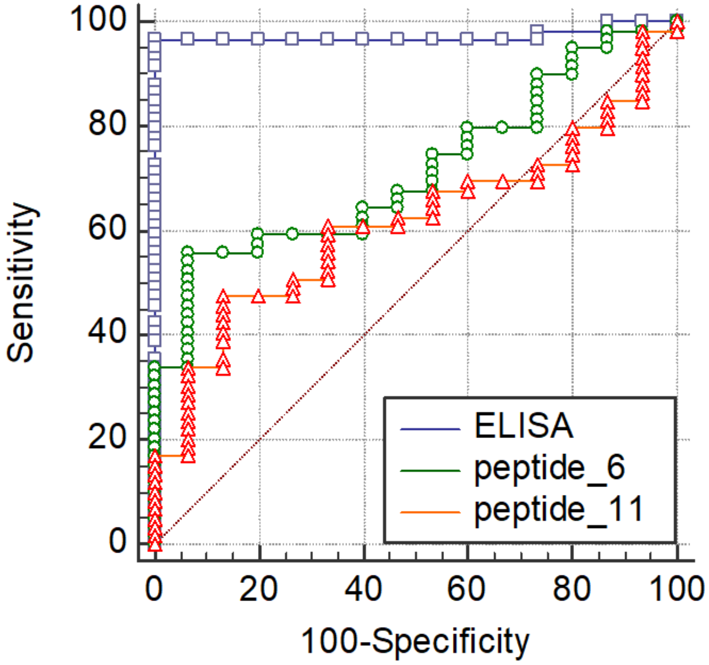

6T

Pairwise comparison of ROC curves

**ELISA ~ peptide_6**	
Difference between areas	0,259
Standard Error[c]	0,0659
95% Confidence Interval	0,130 to 0,388
z statistic	3,924
Significance level	P < 0,0001
**ELISA ~ peptide_11**	
Difference between areas	0,357
Standard Error[c]	0,0729
95% Confidence Interval	0,214 to 0,500
z statistic	4,897
Significance level	P = 0,0001
**peptide_6 ~ peptide_11**	
Difference between areas	0,0983
Standard Error[c]	0,0789
95% Confidence Interval	-0,0563 to 0,253
z statistic	1,246
Significance level	P = 0,2128

a DeLong et al., 1988
b Binomial exact
c DeLong et al., 1988

MedCalc® version 14.8.1

Figure S6: Diagnostic performance of individual peptide antigens and a six-peptide combination (Multi6_sum) in comparison to a commercial ELISA. Receiver operating characteristics (ROC) curves illustrating the diagnostic performance of six individual peptide antigens and their combination (Multi6_sum) using 74 individual sheep serum samples. Samples were classified as Q fever-positive based on commercial ELISA results (S/P%>40), qPCR confirmation of the infection and/or herd status. Score for the combination of six peptides (4; 5; 6; 11; 19 and 25) reflects the number of reactive peptides per serum sample (range 0-6). Vaccinated animals and samples with missing values were excluded from this analysis. Sensitivity (%) is plotted against 100-specificity to evaluate the ability of each peptide to discriminate between Q fever-positive and negative samples.
